# Transformative Impact of Nanocarrier‐Mediated Drug Delivery: Overcoming Biological Barriers and Expanding Therapeutic Horizons

**DOI:** 10.1002/smsc.202400280

**Published:** 2024-09-17

**Authors:** Minhye Kim, Myeongyeon Shin, Yaping Zhao, Mrinmoy Ghosh, Young‐Ok Son

**Affiliations:** ^1^ Interdisciplinary Graduate Program in Advanced Convergence Technology and Science Jeju National University Jeju‐si Jeju Special Self‐Governing Province 63243 Republic of Korea; ^2^ Department of Animal Biotechnology Faculty of Biotechnology College of Applied Life Sciences Jeju National University Jeju‐si Jeju Special Self‐Governing Province 63243 Republic of Korea; ^3^ School of Chemistry and Chemical Engineering Frontiers Science Center for Transformative Molecules Shanghai Jiao Tong University Shanghai 200240 P. R. China; ^4^ Bio‐Health Materials Core‐Facility Center Jeju National University Jeju‐si 63243 Republic of Korea; ^5^ Practical Translational Research Center Jeju National University Jeju‐si 63243 Republic of Korea

**Keywords:** hybrid nanocarriers, liposomes, nanocarriers, nanoemulsions, polymeric

## Abstract

Advancing therapeutic progress is centered on developing drug delivery systems (DDS) that control therapeutic molecule release, ensuring precise targeting and optimal concentrations. Targeted DDS enhances treatment efficacy and minimizes off‐target effects, but struggles with drug degradation. Over the last three decades, nanopharmaceuticals have evolved from laboratory concepts into clinical products, highlighting the profound impact of nanotechnology in medicine. Despite advancements, the effective delivery of therapeutics remains challenging because of biological barriers. Nanocarriers offer a solution with a small size, high surface‐to‐volume ratios, and customizable properties. These systems address physiological and biological challenges, such as shear stress, protein adsorption, and quick clearance. They allow targeted delivery to specific tissues, improve treatment outcomes, and reduce adverse effects. Nanocarriers exhibit controlled release, decreased degradation, and enhanced efficacy. Their size facilitates cell membrane penetration and intracellular delivery. Surface modifications increase affinity for specific cell types, allowing precise treatment delivery. This study also elucidates the potential integration of artificial intelligence with nanoscience to innovate future nanocarrier systems.

## Graphical abstract

The development of drug delivery systems is revolutionizing therapeutic progress by ensuring precise targeting and optimal drug concentrations. Nanocarriers, with their customizable properties and controlled release, overcome biological barriers to improve treatment outcomes. This study explores the potential of integrating artificial intelligence with nanotechnology, offering a glimpse into the future of nanopharmaceuticals.

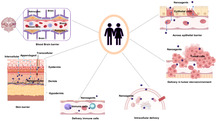

## 1 Introduction

Drug development and delivery are intricate processes requiring substantial investment in time, resources, and expertise. Exhaustive research efforts have been commenced to identify potential drug targets and delve into the intricate biological mechanisms underlying diseases.^[^ ^1^, ^2^ ^]^ The primary challenge in drug delivery is addressing medication side effects, which can frequently cause damage to healthy cells and gastrointestinal (GI) issues. Conventional drug delivery methods face significant challenges, such as limited bioavailability, rapid clearance from the body, and nonspecific distribution, all of which reduce treatment efficacy and increase the risk of adverse effects. For instance, these methods often struggle to maintain therapeutic drug levels within the body due to poor absorption and rapid elimination, leading to suboptimal therapeutic outcomes. Additionally, nonspecific drug distribution can harm healthy tissues along with the targeted disease site.

Advanced solutions like nanocarriers offer promising alternatives. These systems can be engineered for triggered release in response to specific stimuli within diseased tissue—such as changes in pH, redox state, or temperature—enabling more precise drug targeting. Despite these advances, effectively delivering therapeutics to target tissues remains challenging due to biological barriers such as shear pressure, protein adsorption, and rapid clearance. Overcoming these hurdles is essential for achieving successful biodistribution and drug delivery. Consequently, there is a strong demand for innovative approaches like nanocarriers that enhance drug stability, improve targeting precision, and extend circulation time, leading to more effective and safer treatments.^[^ ^3^, ^4^, ^5^, ^6^, ^7^, ^8^ ^]^

Efforts to advance therapeutic progress include the development of drug delivery systems (DDS) capable of sustaining the release of therapeutic molecules.^[^ ^9^, ^10^, ^11^ ^]^ Compared to conventional systems, nano‐DDS offer significant advantages in enhancing therapeutic efficacy.^[^ ^12^, ^13^, ^14^, ^15^ ^]^ These systems improve the pharmacokinetics and pharmacodynamics of encapsulated drugs, ensuring better stability and effectiveness.^[^ ^16^, ^17^, ^18^ ^]^ Recent advancements in nanotechnology, particularly innovative nanomaterials, have introduced promising approaches for diagnosing and treating major diseases^[^ ^18^, ^19^ ^]^ Nanocarriers, typically smaller than 100 nm, have revolutionized the drug delivery field by boosting drug uptake in target tissues, such as tumors, while minimizing off‐target damage.^[^ ^20^ ^]^ In 2021, nanomedicines captured a significant market share of $17 billion within the $203.4 billion pharmaceutical industry. With an anticipated compound annual growth rate (CAGR) of 13.4%, this market is expected to reach $433.9 billion.^[^ ^21^, ^22^ ^]^ Researchers are also exploring polymer–drug conjugates, including those based on polyethylene glycol (PEG) and polymeric micelles, to enhance drug delivery's effectiveness and safety.^[^ ^8^, ^23^, ^24^ ^]^

Since the early 21st century, nanocarriers have evolved from agricultural use to a diverse range of medical applications, underscoring their importance as a key enabling technology. Nanopharmaceuticals have moved from laboratory proofs of concept to commercially available products, improving the safety and efficacy of drug therapies.^[^ ^25^ ^]^ Nanopharmaceuticals have undergone significant evolution, transitioning from early proof‐of‐concept demonstrations in laboratory settings to becoming commercially available products in clinical practice.^[^ ^26^, ^27^, ^28^ ^]^ This evolution has led to substantial improvements in the safety and therapeutic efficacy of encapsulated drugs, making these advanced formulations more effective and reliable for patient care. Emerging technologies to develop nanocarriers, such as nanorobots, when combined with biodegradable polymers, lipid‐based carriers, dendrimers, and micelles nanosystems, hold promise for enhancing drug penetration into diseased tissues.^[^ ^29^, ^30^ ^]^ These sophisticated platforms enable the precise targeting of therapeutic materials to specific tissues and optimize treatment outcomes while minimizing adverse effects. They possess unique properties, including controlled release, reduced degradation, and increased efficacy. Moreover, their small size enables them to traverse cell membranes and deliver materials to the intracellular compartments. Surface modifications further enhance the affinity of nanocarriers for specific cell surfaces, facilitating the precise delivery of multiple treatments to organs or cells.

This review highlights advances in nanomedicine that could enhance the clinical translation of precision medicines, focusing on improving patient‐specific therapeutic responses. Emphasis is placed on using biomaterials and biomedical engineering innovations to overcome biological barriers and patient variability. The review also addresses the biological constraints that have limited the widespread success of nanocarrier applications and critically evaluates rational nanoparticle (NP) designs aimed at overcoming these challenges. Additionally, it explores the potential of combining artificial intelligence (AI) and nanoscience, particularly in modeling methodologies for nanocarriers in drug delivery. These developments are crucial as they pave the way for the clinical translation of nanocarrier‐based precision treatments in medicine, immunology, and in vivo gene editing.

## 2 Organic Nanocarriers

**Figure 1:**
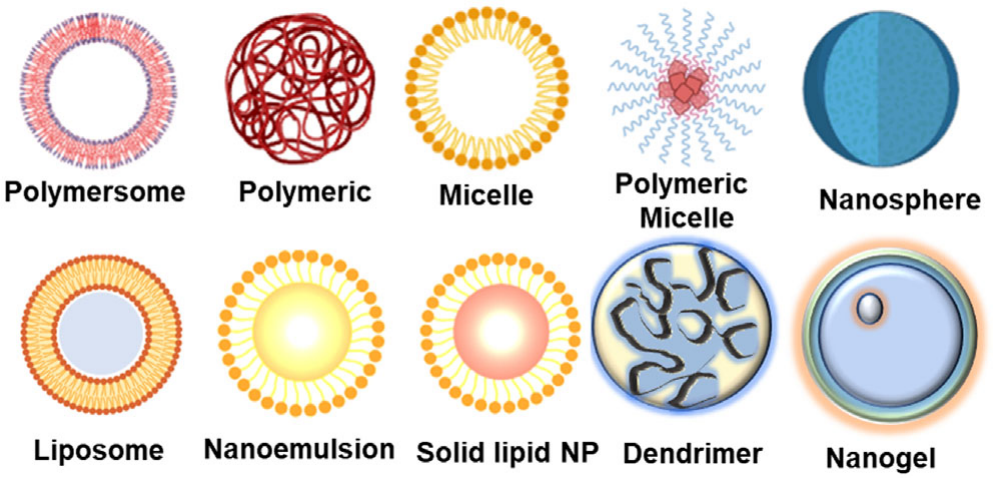
Common biopolymers used in organic nanocarriers. This figure illustrates the diverse array of common biopolymers utilized in the formulation of organic nanocarriers for drug delivery applications. Biopolymers play a critical role in enhancing the stability, biocompatibility, and functionality of nanocarrier systems. Several types of biopolymers, including polymeric NPs, lipid‐based carriers, dendrimers, nanoemulsions, and nanomicelles are highlighted. Each biopolymer offers unique characteristics and functionalities that contribute to their effectiveness in drug delivery. Created with BioRender.com.

Organic nanobased carrier systems constitute a versatile category of solid particles comprising organic compounds, such as lipids and polymers with diameters ranging from 10 to 1 mm.^[^ ^31^, ^32^, ^33^, ^34^ ^]^ Using biopolymers to fabricate these nanocarriers confers several benefits. The synthesis of organic nanocarriers from biopolymers is relatively easy and facilitates efficient production processes. Additionally, these materials exhibit well‐defined functional abilities, allowing their surface properties to be customized for specific applications, such as drug targeting or imaging. Moreover, organic carriers, generally derived from biopolymers, are known for their high biocompatibility within the host organism, minimizing adverse reactions and enabling safe administration in biological systems. They typically demonstrate exceptional stability, ensuring encapsulated cargo preservation during storage and transportation. This stability is essential for maintaining the effectiveness of drugs and the integrity of imaging agents. Biodegradability stands out as another significant advantage of organic carrier fabrication from biopolymers.^[^ ^35^, ^36^ ^]^ As these materials degrade over time, they are metabolized or eliminated from the body, thereby mitigating the risks of long‐term accumulation and potential toxicity. Various common biopolymers have been employed in organic nanocarrier formulation, including polymers, lipid‐based, dendrimers, nanoemulsions, and nanomicelles (**Figure** **1**). Each of these nanocarrier systems possesses unique characteristics and functionalities that make them suitable for various biomedical applications, including drug delivery, imaging, and diagnostics.

### 2.1 Polymeric Nanocarrier

The use of polymers in the formulation of nanocarriers has significantly enhanced their therapeutic effectiveness by enabling site‐specific targeting of these drugs while minimizing their side effects. Polymers have been employed, either alone or in combination with inorganic nanomaterials, to produce multifunctional DDSs.^[^ ^37^ ^]^ Block copolymers, when used in the preparation of drug‐loaded nanocarriers or for surface modification, enhance the interaction between NPs and target tissues, resulting in improved biodistribution, prolonged circulation time, and enhanced uptake.^[^ ^7^, ^38^, ^39^ ^]^ Polymeric nanocarriers are amphiphilic block copolymers with varying hydrophobicity that can be classified into natural and synthetic categories. They can be synthesized using nanoprecipitation or the double‐emulsion method. Given their core–shell structure, they can encapsulate hydrophobic drugs for slow‐release delivery, thereby extending the circulation time within the body. Moreover, the polymeric nanocarrier surfaces can be functionalized with ligands for targeted drug delivery, rendering them highly effective carriers with increased biological activity and bioavailability.^[^ ^40^ ^]^

One of the key advantages of polymeric nanocarriers is their ability to encapsulate various cargo types, including small molecules, nucleic acids (such as DNA and RNA), and proteins (such as peptides and antibodies). This versatility makes them suitable for delivering different types of therapeutics, including chemotherapeutic agents, gene therapies, and biologics. Nanocarriers can achieve drug‐loading percentages ranging from 0.5% to 20% (w/w), depending on the specific agents, the nature of the cargo, and the formulation process. This range allows for the efficient encapsulation of therapeutics while maintaining the stability and integrity of the NPs.^[^ ^7^, ^14^, ^41^, ^42^, ^43^ ^]^

Natural polymeric macromolecules, such as chitosan, alginates, dextran, gelatin, and collagen derivatives, have found extensive applications as nanocarrier materials for treating neuroinflammation.^[^ ^40^ ^]^ Chitosan, which is derived from crustacean exoskeletons and fungal cell walls, exhibits neuroprotective effects in conditions, such as Alzheimer's disease, by inhibiting various pathological factors. Similarly, alginate extracted from marine brown algae and its derivatives effectively modulate neuroinflammatory responses. Synthetic polymers, including polyesters, polyacrylates, and polycaprolactones, offer controllable synthesis conditions and relatively low‐toxicity profiles compared with natural molecules. Polymeric surface modification strategies have been employed to enhance the half life of drugs in blood circulation and improve controlled drug release over extended periods. Notably, poly‐lactic*‐co*‐glycolic acid (PLGA), FDA approved for human use, is one of the most extensively studied polymers due to its excellent biocompatibility and biodegradability. Innovative polymeric nanocarrier systems, such as PLGA‐PEG conjugated with specific peptides and loaded with therapeutic agents, such as curcumin or epigallocatechin‐3‐gallate, have shown promising results in various neurological conditions, including AD and temporal lobe epilepsy.^[^ ^44^, ^45^ ^]^ Furthermore, synthetic polymer‐based systems, such as PLGA NPs, loaded with superoxide dismutase or Foxp3 plasmids, have demonstrated neuroprotective effects by reducing apoptosis and inflammatory markers in cerebral ischemic reperfusion injury models.^[^ ^46^, ^47^, ^48^ ^]^

### 2.2 Liposomes‐Based Nanocarriers

Lipids are amphiphilic molecules that possess a dual nature: one end of the molecule is hydrophilic and the other is hydrophobic (water hating). When exposed to water, lipids undergo self‐assembly because of unfavorable interactions between their hydrophobic segments and the solvent.^[^ ^49^, ^50^ ^]^ Self‐assembly often results in the formation of liposomes, which are vesicles comprising an aqueous core surrounded by a lipid bilayer resembling a biological membrane. Liposomes have been extensively used as DDSs to enhance the therapeutic efficacy of new drugs through various mechanisms. Liposomes can encapsulate various cargo types, with drug‐loading percentages typically ranging from 0.5% to 20%. This flexibility allows for the efficient encapsulation of therapeutics while maintaining the stability and integrity of the NPs.^[^ ^51^, ^52^ ^]^ By encapsulating drugs within their lipid bilayers or aqueous cores, liposomes can modify drug absorption, reduce metabolism, prolong the biological half‐life, and mitigate toxicity. This alteration in drug distribution was primarily governed by the properties of the carrier rather than solely by the physicochemical characteristics of the drug substance. Hydrophobic drugs are accommodated within the liposomal bilayer, whereas hydrophilic drugs are trapped within the aqueous core or the bilayer interface. As a result, liposomal formulations have demonstrated enhanced therapeutic efficiency in preclinical models and clinical trials compared to conventional formulations owing to their ability to modulate biodistribution. It can also efficiently encapsulate nucleic acids such as DNA, RNA, and siRNA for gene therapy applications. The lipid bilayers of liposomes protect nucleic acids from degradation by nucleases and facilitate their delivery to target cells for therapeutic gene expression.^[^ ^53^, ^54^ ^]^ Liposomes are biodegradable, biologically inert, and nonimmunogenic lipids that minimize the risk of immune reactions or adverse effects upon administration. Moreover, these compounds exhibit limited toxicity and do not elicit pyrogenic or antigenic responses. These favorable properties, coupled with the ease of surface modification for targeting specific tissues or cells, render liposomes attractive candidates for drug delivery vehicles compared to other systems, such as NPs and microemulsions.^[^ ^55^ ^]^

Furthermore, the modification of liposome‐based nanocarriers offers versatility and advanced capabilities to deliver various biologically active compounds.^[^ ^56^ ^]^ The selection of appropriate preparation methods allows the generation of liposomes with varying sizes, lamellarities, and physicochemical properties, thereby influencing the final amount of the encapsulated drug. The modification of liposomes enables passive or active targeting of specific sites, such as tumors, thus minimizing off‐target effects on nonmalignant cells (**Table** **1**). Studies have demonstrated the efficacy of modified liposomal formulations in enhancing drug therapeutic effects, as evidenced by the increased survival rates in animal models of diseases such as leukemia. “Stealth liposomes” are advanced liposomes that have been stabilized with polyethylene glycol (PEG) to enhance their circulation time in the bloodstream. These liposomes can be further modified to deliver targeted drugs by incorporating monoclonal antibodies, peptides, growth factors, glycoproteins, carbohydrates, or receptor ligands. Additionally, researchers are exploring the use of synthetic polymers like polyvinylpyrrolidone (PVP), polyacrylic acid (PAA), and 1,2‐distearoyl‐sn‐glycero‐3‐phosphatidylethanolamine (DSPE), which are covalently bonded to poly(2‐methyl‐2‐oxazoline) or poly(2‐ethyl‐2‐oxazoline), to further enhance the stability and effectiveness of these liposomal carriers.^[^ ^25^ ^]^

**Table 1:** The modified lipid‐based nanocarriers.

Type	Composition	Function/Application	Advantages	References
Asymmetric oxygen carrier system (AOCS) liposomes	Perfluorocarbon core with a monolayer phospholipid shell	Carries oxygen into the skin, used in oxygenation therapies	Provides sustained oxygen delivery, improves skin vitality	[257]
Bilosomes	Modified niosomes containing bile salts	Oral vaccine delivery; enhances mucosal immunity	Protects antigens from GI degradation, enhances mucosal absorption	[258]
Dendrosomes	Liposomes encapsulating dendrimers	Drug delivery; being investigated for enhanced drug and gene delivery	Enhances drug solubility and stability, potential for targeted delivery	[259]
Ethosomes	Phospholipids and ethanol	Transdermal drug delivery; enhances skin permeation	Efficient in delivering drugs to deeper skin layers	[260]
Lipofectamine	A mixture of cationic lipids (e.g., 2,3‐dioleoyloxy‐N‐[2(sperminecarboxamido)ethyl]‐N,N‐dimethyl‐1‐propaniminium trifluoroacetate) and 1,2‐dioleoyl‐sn‐glycerophosphoethanolamine	Commonly used in gene transfection to deliver RNA or DNA into cells	High transfection efficiency, improves stability of genetic material	[261]
Lipoplexes	Complexes between nucleic acids and cationic lipids	Cytosolic delivery of microRNA (miRNA) in cancer therapy	Effective in gene delivery, enhances cellular uptake of genetic material	[262]
Nanotopes	Nanocarriers with a monolayer phospholipid membrane	Small particle size (20–40 nm) used for better stability in drug delivery	Enhanced stability, small particle size, efficient delivery	[263]
Niosomes	Nonionic surfactant combined with cholesterol	Drug delivery enhances stability and bioavailability	Biocompatible, stable, low cost	[260]
Pharmacosomes	Colloidal vesicles or hexagonal arrangement of colloidal drug dispersions covalently attached to phospholipids	Advanced DDS, improves bioavailability and targeting	High stability, tailored drug release profiles	[264]
Photosomes	Liposomes encapsulating photolysase enzyme extracted from Anacystis nidulans	Incorporated into sun care products to protect and repair sun‐exposed skin	Photoprotective, enhances DNA repair in skin cells	[265]
Phytosomes	Complexes between phospholipids and botanical derivatives (e.g., catechin, quercetin)	Enhances the bioavailability of botanical extracts for therapeutic applications	Improves absorption and bioavailability of plant‐derived compounds	[257]
Spongosomes	Sponge‐like liquid crystalline nanodelivery systems made of amphiphilic lipids and surfactants	DDSs with controlled release properties	High drug loading capacity, controlled release	[266]
Transfersomes	Phospholipids and surfactant	Transdermal drug delivery; improves penetration through the skin	High deformability, enhanced skin penetration	[260]
Ultrasomes	Specialized liposomes encapsulating UV endonuclease enzyme	Used in cosmetics to repair UV‐induced skin damage	Specific for UV damage repair, utilized in high‐end skincare products	[267]
Vesosomes	Liposomes encapsulating smaller liposomes	Drug delivery; allows for multicomponent delivery systems	Capable of delivering multiple drugs simultaneously, provides enhanced protection for encapsulated drugs	[259]
Virosomes	Liposomes reconstituted from viral envelope phospholipids with the nucleocapsid removed	Investigated for vaccine delivery, mimics viral entry into cells	Efficient in delivering antigens to elicit an immune response, used in vaccine development	[268]
Yeast‐based liposomes	Yeast extract combined with liposomes	Cosmetics for skin repair and oxygenation	Natural origin; enhances skin regeneration	[257]

Despite their numerous benefits, liposomes encounter challenges such as degradation, macrophage scavenging, and nonspecific targeting of other tissues. To overcome these limitations, researchers have developed strategies, such as long‐circulation liposomes, actively targeted liposomes, and novel formulations. For example, dopamine‐PEGylated immune‐liposomes have been engineered for Parkinson's disease treatment, demonstrating enhanced brain uptake compared with free dopamine and conventional liposomal formulations.^[^ ^57^ ^]^ Tailoring the physicochemical properties of liposomes, such as utilizing pH‐stable lipids such as dipalmitoyl phosphatidylcholine, has enabled the sustained release of drugs and has exhibited therapeutic effects in neuroinflammatory conditions and ischemia‐reperfusion injuries. These advancements underscore the potential of liposomes as effective carriers for drug delivery, paving the way for innovative therapeutic interventions.^[^ ^57^, ^58^ ^]^

### 2.3 Solid Lipid‐Based Nanocarrier

To overcome the limitations of polymeric nanocarriers, lipid‐based carriers have emerged as promising alternatives, particularly lipophilic pharmaceuticals. Among these lipid nanocarriers, solid lipid NPs (SLNs) have gained considerable attention from formulators worldwide.^[^ ^59^, ^60^ ^]^ Developed over the past decade, SLNs represent a novel generation of submicrometer‐sized lipid emulsions, in which the liquid lipid component is replaced with a solid lipid. This substitution offers distinct advantages to SLNs, including small size, large surface area, high drug‐loading capacity, and interactions between phases at the interfaces, making them attractive for enhancing the performance of pharmaceuticals, nutraceuticals, and other materials. Manufactured from either synthetic or natural lipids, SLNs feature a lipid core that remains in a solid state at both room and body temperatures.^[^ ^61^ ^]^ Compared with cationic liposomes, SLNs are generally considered less toxic and are recognized as safe for human use. These submicrometer colloidal carriers comprise physiological lipids dispersed in water or an aqueous surfactant solution, offering physiological tolerance and superior drug delivery efficiency compared to other lipid‐based nanocarrier formulations.^[^ ^62^, ^63^, ^64^ ^]^

SLNs have garnered significant interest as novel colloidal drug carriers for intravenous applications and have demonstrated superior efficacy in various therapeutic applications. For example, SLNs loaded with curcumin demonstrated enhanced anti‐amyloid‐beta, anti‐inflammatory, and neuroprotective effects compared to traditional curcumin formulations in mouse models of AD. Additionally, sesamol‐loaded SLNs have been found to alleviate oxidative stress and mitigate neuroinflammation and memory deficits in experimental models of neurodegenerative diseases.^[^ ^65^ ^]^

### 2.4 Dendrimers

Dendrimers, a class of highly organized macromolecules synthesized through the repetitive chemical reactions of core structures, have been extensively investigated. These dendrimers serve as highly effective carriers for delivering drugs, genes, and proteins across barriers through mechanisms such as covalent bonding, ion interactions, or adsorption.^[^ ^66^, ^67^, ^68^ ^]^ Their advantages include precisely controlled biodistribution and pharmacokinetics, notable structural and chemical uniformity aiding reproducibility, the ability to interact with various compounds or ligands to enhance solubility and specificity, and multifunctionality due to abundant surface groups. The drug‐loading capacity of dendrimers typically falls within 1%–5%, allowing for efficient encapsulation while maintaining the structural integrity and stability of the dendrimer–drug complexes.^[^ ^69^, ^70^ ^]^ The administration of specific dendrimers in animal models results in transient elevations in liver enzyme levels and leukocyte infiltration.^[^ ^71^, ^72^ ^]^ However, other studies have highlighted the beneficial effects of dendrimers on human health. Dendrimer‐based therapies have demonstrated promise in addressing conditions, such as neuroinflammation and cerebral palsy, by elevating antioxidant levels and mitigating neuroinflammation.^[^ ^73^, ^74^, ^75^ ^]^ Dendritic polyglycerol sulfates have emerged as potent inhibitors of inflammation and complement activation. Studies have indicated that dPGS can disrupt the formation of amyloid‐beta fibrils, decrease the production of neuroinflammatory lipocalin‐2, and normalize impaired neuroglial cells, suggesting potential applications in treating neuroinflammatory conditions and neurotoxicity associated with neurodegenerative diseases, such as AD.^[^ ^76^, ^77^, ^78^, ^79^ ^]^

Poly (amidoamine) dendrimers are used as nanocarriers due to the presence of tertiary amine groups at their branching points. The metal ions introduced into aqueous dendrimer solutions form complexes with these amine groups, which are then reduced to form nanocarriers encapsulated within the dendrimer structure. Various dendrimeric formulations have been employed as nanodrugs for treating diverse diseases, including antiviral formulations containing polylysine dendrimers with sulfonated naphthyl groups, antibacterial agents comprising PPL dendrimers with tertiary alkylammonium groups, and chitosan dendrimer hybrids used for antibacterial purposes. When combined with anti‐inflammatory and antioxidant drugs, advanced poly (amidoamine) dendrimers exhibit the potential to attenuate inflammation in traumatic brain injury by inhibiting proinflammatory cytokines and reducing NF‐κB activation. Similarly, dendrimer‐based N‐acetyl‐L‐cysteine formulations have been shown to increase intracellular antioxidant levels and prevent excitotoxicity in microglia and astrocytes. Dendrimeric formulations incorporated into hydrogels function as crosslinked networks that expand in an aqueous solution. These formulations, which are often modified with polyethylene glycol groups, are useful for cartilage tissue engineering, ocular injury sealing, and targeted drug delivery. Additionally, dendrimers have shown promise in transdermal drug delivery by enhancing solubility and plasma circulation, forming complexes with drugs like nonsteroidal anti‐inflammatory agents to enhance skin permeation and acting as permeation enhancers.^[^ ^66^, ^73^ ^]^

### 2.5 Nanoemulsions

Nanoemulsions are colloidal dispersions composed of two immiscible liquids stabilized by surfactants. Typically, an nanoemulsion (NE) comprises water, oil, and emulsifiers in appropriate proportions. NEs offer several advantageous properties, such as excellent biocompatibility, kinetic stability, cell transport facilitation through paracellular and transcellular pathways, and protection against hydrolysis and enzymatic degradation of residues.^[^ ^80^ ^]^ These versatile systems are adept at encapsulating components dissolved in the dispersed phase. Direct systems such as oil‐in‐water nanoemulsions are suitable for encapsulating hydrophobic components, whereas inverse systems facilitate hydrophilic cargo encapsulation. NEs can be administered via the nasal, ocular, oral, and intravenous routes.

Chitosan‐coated rosmarinic acid nanoemulsions have demonstrated protective effects by inhibiting cellular death and restoring the astrocyte redox state during lipopolysaccharide‐induced neuroinflammation and oxidative stress in astrocytes. Studies demonstrated that siRNA nanoemulsions markedly reduced the levels of TNF‐α, a pro‐inflammatory signaling molecule. Therefore, nanoemulsions encapsulated with TNF‐α siRNA hold promise as potential candidates for treating neuroinflammation.^[^ ^81^ ^]^ Ropinirole, a dopamine agonist used in combination therapy with levodopa for Parkinson's disease, has limitations due to its low bioavailability and short half life. Indeed, modification of the ropinirole NE gel for transdermal delivery has shown improved drug absorption and reduced skin irritation and toxicity compared with ropinirole alone.

### 2.6 Micelles as Nanocarriers

Micelles, which are self‐assembling nanosized colloidal particles characterized by a hydrophobic core and hydrophilic shell, serve as effective pharmaceutical carriers for water‐insoluble drugs, offering several desirable properties. Amphiphilic copolymers, composed of hydrophobic and hydrophilic blocks, are gaining attention as compounds capable of forming micelles. Polymeric micelles, including lipid‐core micelles formed by the conjugation of soluble copolymers with lipids, exhibit high stability, both in vitro and in vivo, along with good biocompatibility. They can solubilize various poorly soluble pharmaceuticals, many of which are undergoing preclinical and clinical trials.^[^ ^82^, ^83^ ^]^

The most commonly used hydrophilic segment in micelles for drug delivery is poly(ethylene glycol) (PEG), which typically has a molecular weight of 2–15 kDa. PEG, known for its high water solubility, nontoxicity, and neutral charge, forms a hydrophilic corona on the micelle surface, minimizing nonspecific interactions with blood components and prolonging circulation time. Besides PEG, other polymers such as poly(N‐vinyl pyrrolidone) (PVP) and poly(N‐isopropyl acrylamide) have also been employed as hydrophilic portions of the micelles. Another type of polymeric micelle utilizes lipids as the hydrophobic core, as exemplified by PEG‐diacyllipid conjugates synthesized by Torchilin's group. These micelles exhibited strong hydrophobic interactions between the double acyl chains, resulting in stable micelles with low critical micelle concentration values. They can effectively solubilize various poorly water‐soluble drugs and demonstrate favorable stability, prolonged circulation in the blood, and tumor accumulation via enhanced permeability and retention.^[^ ^84^ ^]^ Further development of micellar systems composed of PEG–cholic acid conjugates, which exhibit a high loading capacity for drugs, such as paclitaxel. These micelles have demonstrated improved anticancer efficacy and reduced toxicity compared with conventional formulations in preclinical models and clinical trials. Additionally, micellar carriers like PEG2K–cholic acid have shown higher drug loading capacities and sustained drug release profiles, offering promising prospects for drug delivery applications.^[^ ^84^ ^]^ Yokoyama et al. reported that adriamycin‐conjugated polyethylene glycol–poly(aspartic acid) block copolymers (PEG‐P[Asp(ADR)]) formed micelles in aqueous solution and demonstrated significant antitumor activity in vivo.^[^ ^85^ ^]^ However, this study did not analyze the ratio of chemically or physically entrapped adriamycin, and certain amounts of adriamycin derivatives were incorporated into the micelles as impurities. These impurities can cause adverse effects.

Micelles, which are self‐assembling nanosized colloidal particles characterized by a hydrophobic core and hydrophilic shell, serve as effective pharmaceutical carriers for water‐insoluble drugs, offering various desirable properties. Amphiphilic copolymers, composed of hydrophobic and hydrophilic blocks, are gaining attention as compounds capable of forming micelles. Polymeric micelles, including lipid‐core micelles formed by the conjugation of soluble copolymers with lipids, exhibit high stability, both in vitro and in vivo, along with good biocompatibility. They can solubilize various poorly soluble pharmaceuticals, many of which are undergoing preclinical and clinical trials.^[^ ^82^, ^83^ ^]^

The most commonly used hydrophilic segment in micelles for drug delivery is PEG, which typically has a molecular weight of 2–15 kDa. PEG, known for its high water solubility, nontoxicity, and neutral charge, forms a hydrophilic corona on the micelle surface, minimizing nonspecific interactions with blood components and prolonging circulation time. In addition to PEG, other polymers such as PVP and poly(N‐isopropyl acrylamide) have also been employed as hydrophilic portions of the micelles. Another type of polymeric micelle utilizes lipids as the hydrophobic core, as exemplified by PEG–diacyllipid conjugates synthesized by Torchilin's group. These micelles exhibited strong hydrophobic interactions between the double acyl chains, resulting in stable micelles with low critical micelle concentration values. They can effectively solubilize various poorly water‐soluble drugs and demonstrate favorable stability, prolonged circulation in the blood, and tumor accumulation via enhanced permeability and retention.^[^ ^84^ ^]^ Further development of micellar systems composed of PEG‐cholic acid conjugates, which exhibit a high loading capacity for drugs, such as paclitaxel. These micelles have demonstrated improved anticancer efficacy and reduced toxicity compared with conventional formulations in preclinical models and clinical trials. Additionally, micellar carriers like PEG2K‐ cholic acid have shown higher drug loading capacities and sustained drug release profiles, offering promising prospects for drug delivery applications.^[^ ^84^ ^]^ Yokoyama et al. reported that adriamycin‐conjugated polyethylene glycol–poly(aspartic acid) block copolymers (PEG‐P[Asp(ADR)]) formed micelles in aqueous solution and demonstrated significant antitumor activity in vivo.^[^ ^85^ ^]^ However, this study did not analyze the ratio of chemically or physically entrapped adriamycin, and certain amounts of adriamycin derivatives were incorporated into the micelles as impurities. These impurities can cause adverse effects.

## 3 Polymeric Lipid Hybrid Nanocarriers

**Figure 2:**
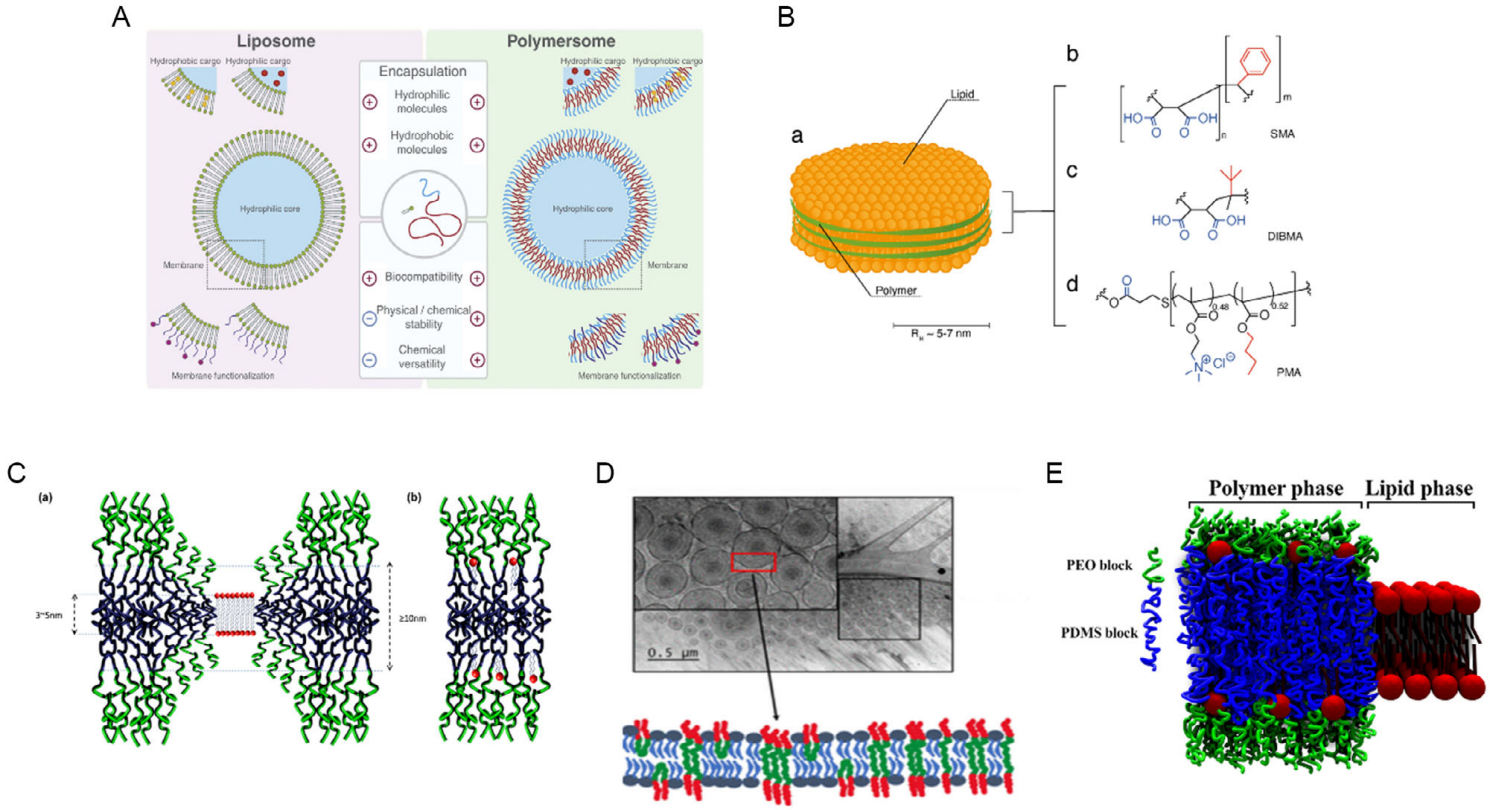
The diagram illustrates the structure of LPNPs, highlighting the interaction between lipids and polymers. A) The structure of a liposome features an aqueous core surrounded by a bilayer membrane. This membrane consists of hydrophobic tails forming the interior of the bilayer, while hydrated hydrophilic heads stabilize the outer and inner surfaces. Polymersomes, which are synthetic counterparts to liposomes, are composed of amphiphilic block copolymer membranes. Although both carriers share many similar properties, polymersomes offer greater versatility and improved stability. Adapted with permission.^[^ ^238^ ^]^ Copyright 2014, Elsevier. B)(a) The illustration shows LPNPs created through interactions with (b–d) different polymers, where the lipids (depicted in yellow) are enveloped by a “belt” of polymer (depicted in green). Different polymers can be used to create this polymer–lipid combination, with each polymer influencing the NP's properties, such as its stability, drug release profile, and interaction with biological systems. Hydrophilic groups are highlighted in blue, and hydrophobic groups are highlighted in red. ^[^ ^239^ ^]^ C) (a) Conformational adaptation at the polymer/lipid domain boundary in hybrid vesicles is expected when domain formation occurs, allowing the components to adjust and integrate within distinct regions. (b) In contrast, the absence of conformational adaptation between the polymer and lipid boundaries results in a homogeneous mixture, where the components are evenly distributed without distinct domain separation.^[^ ^88^ ^]^ D) Hybrid lipid/polymer vesicle systems, which incorporate the lipid 1,2‐dioleoyl‐sn‐glycero‐3‐phosphocholine (DOPC) and amphiphilic triblock copolymers composed of poly(ethylene glycol)–poly(dimethylsiloxane)–poly(ethylene glycol) (PEG–PDMS–PEG), are studied to understand how hydrophilic blocks impact vesicle morphology. The hydrophilic mass fraction of the amphiphilic polymer plays a significant role in influencing the membrane thickness, size polydispersity, and lamellarity of these polymer/lipid hybrid vesicles. Changes in the hydrophilic mass fraction can lead to variations in how the vesicles form, including their structural uniformity and stability, making this a crucial factor in the design and application of such hybrid systems. Adapted with permission.^[^ ^92^ ^]^ Copyright 2022, ACS. E) The schematic view of a hybrid polymer/lipid membrane with lateral phase separation typically shows a membrane where distinct regions or “domains” of polymers and lipids coexist within the same membrane. Adapted with permission.^[^ ^93^ ^]^ Copyright 2022, Elsevier.

The formation of stable hybrid vesicles in polymeric–lipid hybrid nanocarriers is primarily determined by the differences in chemical composition and the size of the hydrophobic segments between polymers and lipids (**Figure** **2**). This hybrid nanocarrier system integrates the essential features of both polymers and lipids into a single, unified structure,^[^ ^86^ ^]^ allowing for the creation of various combinations that effectively address the limitations of individual components within the hybrid framework. Polymers play a crucial role in ensuring sustained drug release, enhancing the architectural stability and durability of the hybrid system. Meanwhile, lipids, with their biomimetic properties, significantly improve the drug‐loading capacity of these carriers. Compared with nanomaterials composed solely of either polymers or lipids, hybrid particles demonstrate superior and prolonged efficacy in vivo.^[^ ^87^ ^]^

Furthermore, the vesicular structures that emerge are influenced by factors such as the polymer‐to‐lipid molar ratio and the thermodynamic phase of the phospholipids. These structures can range from a homogeneous mixture of polymers and lipids to distinct microdomains rich in either polymer or lipid.^[^ ^88^ ^]^ For instance, adjusting the polymer/lipid molar ratio can result in vesicles with varying internal architectures. A higher polymer content may lead to thicker, more stable vesicles, while a higher lipid content might produce more flexible structures with specific biological functionalities.

The thermodynamic phase of the lipid component, often defined by the lipid's main chain transition temperature (*T*_*m*_), is a crucial factor in determining membrane properties. Below *T*_*m*_, lipids exist in a gel‐like state, resulting in more rigid and ordered membrane structures. Above *T*_*m*_, lipids transition to a fluid, liquid‐crystalline state, leading to more flexible and dynamic membranes. In hybrid vesicles, this phase behavior is comparable to that observed in mixed liposomes made from lipids with varying *T*_*m*_ values.^[^ ^89^, ^90^ ^]^

The interaction between polymers and lipids in hybrid vesicles is significantly influenced by the size mismatch between polymer chains and lipid tails. Liposomes typically have a membrane thickness of 3–5 nm, while polymersomes can vary between 5 and 50 nm. When there is a notable size disparity between the polymer and lipid components, it can create high line tension at the interface between lipid‐rich and polymer‐rich regions. This tension arises from the thickness mismatch, which can expose hydrophobic polymer segments to the aqueous environment, increasing the system's overall energy.^[^ ^88^ ^]^

To mitigate this energetic penalty, the system may adopt one of two possible strategies: conformational adaptation or calescence into larger domains. In conformational adaptation, the polymer chains at the boundary between lipid and polymer domains may undergo elastic deformation.^[^ ^88^, ^91^, ^92^ ^]^ This deformation helps to reduce the line tension but comes at the cost of reduced entropy, as the number of possible conformations for the polymer chains decreases. This situation is somewhat analogous to the elastic deformation observed in lipid membranes with coexisting phases, where the membrane bends to accommodate different lipid compositions. For example, in a hybrid vesicle composed of a rigid polymer like polystyrene and a fluid lipid like dioleoylphosphatidylcholine (DOPC), the polymer may deform at the boundary with the lipid domain to minimize exposure to water, resulting in a more stable interface.

Alternatively, if the conformational adaptation of the polymer chains is insufficient to stabilize the structure, the system may allow the lipid or polymer domains to grow larger. This reduces the overall interface area between mismatched regions, thus minimizing the line tension and associated energy cost. In a system where the polymer chains are too rigid or too large to deform easily, such as in hybrid vesicles made with a block copolymer like polyethylene glycol–polylactic acid (PEG–PLA) and a lipid like dipalmitoylphosphatidylcholine (DPPC), the vesicle might form larger lipid domains surrounded by polymer regions to reduce boundary exposure.

The ability of a hybrid vesicle to adapt to various conditions is largely determined by the molecular characteristics of the polymer, including its molar mass, chain length, and the rigidity of its hydrophobic backbone. If the polymer chains are too rigid or if there is a significant size mismatch, the system may fail to form stable domains, resulting in a homogeneous mixture where the components are evenly distributed throughout the vesicle. A study that addressed the line tension in hybrid polymer/lipid vesicles found that the values are comparable to those observed in multiphase lipid vesicles. The authors emphasized that determining the line tension in polymer/lipid hybrid systems is crucial for better understanding the structure and properties of these hybrid membranes.^[^ ^93^ ^]^

**Figure 3:**
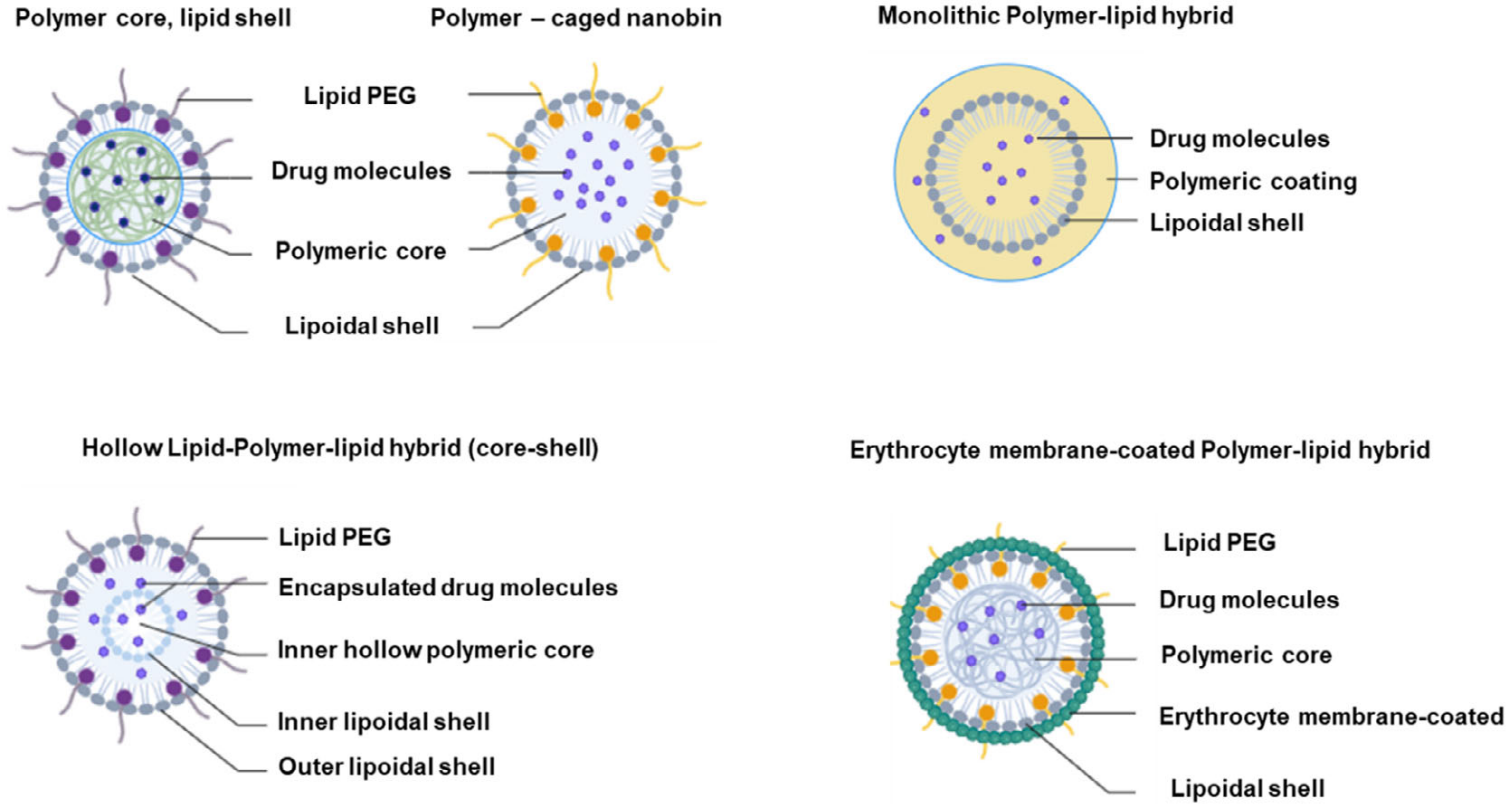
Hybrid structures combining polymers and lipids in nanocarrier design. This figure illustrates the concept of hybrid structures that combine polymers and lipids into a single entity, offering significant potential as versatile and efficient drug delivery carriers. Hybridization of polymers and lipids results in nanocarriers with enhanced properties, including improved stability, biocompatibility, and drug‐loading capacity. The figure depicts the structural composition of these hybrid nanocarriers, highlighting the integration of the polymer chains and lipid bilayers. Created with BioRender.com.

By integrating polymers and lipids into a single entity, hybrid structures present significant potential as highly effective drug delivery carriers^[^ ^94^, ^95^ ^]^ (**Figure** **3**). With precise engineering and formulation techniques, these hybrid structures can be tailored to optimize drug delivery for various therapeutic applications (**Table** **2**).

**Table 2:** Detailed overview of different types of hybrid nanocarriers, focusing on their stability, biocompatibility, and drug‐loading capacity.

Type	Stability	Biocompatibility	Drug‐Loading Capacity
Polymer core–lipid shell hybrid carriers	High stability: The degradation half life of the polymer core is typically days to weeks depending on the polymer used (e.g., PLGA has a half life of ≈714 days in vivo).	Good biocompatibility: Cell viability typically exceeds 85–95% in vitro, with low hemolysis rates (<5%).	20–40% w/w: The polymer core can encapsulate a large amount of hydrophobic drugs, with lipid shells providing additional stabilization.
Polymer‐caged nanobins (PCNs)	Moderate‐to‐high stability: The polymer cage improves the structural integrity, leading to degradation half lives ranging from 1 to 2 weeks.	Enhanced biocompatibility: In vitro studies show cell viability rates of 80—90%, with minimal immune activation.	10–30% w/w: The encapsulation of drugs within the liposomal core, combined with the polymer cage, stabilizes sensitive drugs but may limit loading capacity.
Monolithic LPNPs	High stability: Depending on the polymer–lipid composition, degradation can be slow with 2–4 weeks in vivo half lives.	Good‐to‐excellent biocompatibility: Cell viability is generally 90–98%, with very low cytotoxicity and negligible immune response.	30–50% w/w: LPNPs offer high drug‐loading efficiency for both hydrophobic and hydrophilic drugs, with controlled release profiles.
Lipid–bilayer‐coated nanocarriers	High stability: The lipid bilayer adds stability to the core, leading to 1–3 weeks of half lives depending on the core material.	Excellent biocompatibility: Cell viability typically exceeds 90–99% with low immunogenicity and hemolysis rates (<3%).	10–40% w/w: Depending on the core material, these nanocarriers can encapsulate various drugs, with the bilayer aiding in drug retention and stability.
Hollow‐core NPs	Moderate‐to‐high stability: Stability is enhanced by the shell material, with half lives ranging from 1 to 3 weeks depending on the shell composition.	Good biocompatibility: Typically 85–95% cell viability with low‐to‐moderate immune response, depending on the core and shell materials used.	30–60% w/w: The hollow core allows for very high drug‐loading capacity, particularly advantageous for large molecules or multidrug formulations.

### 3.1 Polymer Core–Lipid Shell Hybrid Carriers

Hybrid polymer core–lipid shell nanocarriers represent a significant advancement in drug delivery technology and have been extensively studied owing to their promising applications. These nanocarriers comprise a polymeric core surrounded by a lipid shell that combines the advantages of both materials. Initially used in biomedical and biotechnological devices, hybrid nanocarriers were created by mixing liposomes with polymeric nanocarriers, resulting in a composite structure with a polymeric core and a lipid coating. Typically, one or two lipid layers envelop the polymer backbone, with water or a buffer filling the gaps. These lipid shells are composed of either cationic or anionic phospholipids that facilitate electrostatic interactions between oppositely charged components. Three main layers were identified in these hybrids: a biodegradable hydrophobic polymer (such as PLGA), a lipid–PEG conjugate shell capable of incorporating hydrophilic drugs, and an intermediate lipid layer that acts as a barrier to prevent leakage or water penetration. This layered structure ensures robust formulation integrity.^[^ ^87^, ^96^ ^]^

Although the loading of water‐soluble ionic drugs presents challenges, hydrophobic drugs can be readily incorporated into hybrid nanocarriers. However, the complex formation of ionic drugs with counterion polymers may improve their loading efficiency. Additionally, the outer lipid shell enhances the integrity of the system. Researchers have demonstrated the versatility of these polymer–lipid hybrids by modifying SLNs to encapsulate positively charged hydrophilic drugs within an anionic polymer matrix. For example, salidroside, a water‐soluble antitumor compound, was successfully entrapped in a polymeric core–lipid shell (PLGA–PEG–PLGA) system, resulting in improved drug entrapment capacity, enhanced tumor cell uptake, and smaller particle size.^[^ ^94^ ^]^ Similarly, enoxaparin was encapsulated in alginate‐coated chitosan nanocarriers, where the cationic polymer chitosan acted as a charge stabilizer.

### 3.2 Polymer‐Caged Nanobins

Polymer‐caged nanobins represent a sophisticated approach for achieving desired properties in DDSs, such as enhanced drug release, improved stability, and minimized leakage.^[^ ^97^ ^]^ These particles were coated with additional polymer layers on the outside, offering opportunities for customization and functionality. The outer layers of polyacrylic acid can be used to introduce specific surface features. By attaching various linkers via carboxylic groups to these polymers, pH‐sensitive reactions can be achieved.^[^ ^98^, ^99^ ^]^ The preparation of liposomes with polymers involves the modification of their surface properties to attain optimal characteristics. Initially, the liposomes were created as base structures. Subsequently, polymeric coats, including cholesterol‐functionalized polyacrylic acid, were applied to the surfaces. This process involves the addition of carboxylate groups to the liposomal surfaces to enhance their functionality. The drug‐release profiles of these systems can be tailored to meet specific requirements. Another strategy involves using pH‐responsive crosslinked polymers to shield the liposome environment.^[^ ^100^ ^]^ This polymer coating offers significant advantages, including the protection of liposomes, increased durability, minimized drug degradation due to leakage, and enhanced drug release at targeted sites.

### 3.3 Monolithic LPNPs‐Mixed Lipid–Polymer

Monolithic lipid polymer nanoparticles (LPNPs)/mixed lipid polymers represent an innovative strategy for nanocarrier formulation, offering a unique combination of lipid and polymer components for drug delivery applications.^[^ ^101^ ^]^ These nanocarriers were designed with lipids dispersed within a polymer matrix, resembling colloidal carriers tailored for efficient drug distribution throughout the body. These nanocarriers, which are composed of a combination of copolymers with both hydrophilic and hydrophobic segments, as well as lipids, have amphiphilic features that are required for drug encapsulation and delivery. These hybrid nanocarriers, such as liposomes, which are composed of phospholipids that form vesicular structures resembling cell membranes, exploit the self‐assembling characteristics of phospholipids. However, unlike conventional liposomes, the structural configuration of monolithic LPNPs/mixed lipid–polymer NPs offers distinct advantages and challenges. While liposomes can be easily modified by PEG to enhance their stability and circulation time in the bloodstream, the incorporation of PEG‐lipid conjugates at high densities may compromise the structural integrity of phospholipids within these hybrid nanocarriers. This limitation arises from the tendency of phospholipids to form micelles rather than maintaining vesicular structures under such conditions. Despite this challenge, monolithic LPNPs‐mixed lipid–polymer NPs hold promise as versatile drug delivery vehicles.^[^ ^102^, ^103^ ^]^ By combining the benefits of lipids, such as biocompatibility and membrane‐mimicking properties, with those of polymers, such as controlled release and stability, they offer a platform for targeted and sustained drug delivery.

### 3.4 Lipid Bilayer‐Coated Nanocarriers

Lipid bilayer‐coated NPs represent an innovative approach for enhancing the stability and functionality of DDSs, particularly in cases where polyethylene glycol alone may not be sufficient for in vivo particle stabilization. These nanocarriers are coated with lipid bilayers derived from red blood cell membranes, offering unique advantages in terms of biocompatibility and prolonged drug release. The process of creating lipid bilayer‐coated nanocarriers typically involves the heat treatment of red blood cells (RBCs) to generate membrane‐derived particles. During this process, polymeric nanocarriers are encapsulated within lipid bilayers derived from erythrocyte membranes. This encapsulation process effectively targets the drug within the polymeric nanocarriers, providing lipid‐coated nanocarriers with enhanced biological surface strength and maximum in vivo stability. Encapsulation of polymeric nanocarriers within lipid bilayers mimics the complex surface chemistry of RBCs, offering a biomimetic environment for drug delivery. The drug payload is enclosed within vesicles formed by the erythrocyte membrane, enabling prolonged drug release from the lipid bilayer. The dense lipid barrier contributes to a slower release profile, ensuring sustained therapeutic efficacy. However, the presence of various blood type antigens in RBC membranes can pose challenges in terms of crossmatching during blood transfusion.^[^ ^104^, ^105^, ^106^ ^]^ Despite these considerations, lipid bilayer‐coated nanocarriers have significant potential as effective and biocompatible drug delivery vehicles, offering controlled release and enhanced stability for various therapeutic applications.

### 3.5 Hollow‐Core Nanoparticles/Lipid–Polymer–lipid Nanocarriers

These nanocarriers have concentric structures with an innermost hollow layer of lipids, a middle layer of polymers, and an outermost lipid–PEG layer. Fabricating these lipid–polymer–lipid nanocarriers typically involves a double‐emulsification solvent evaporation method.^[^ ^95^, ^107^ ^]^ During this process, lipids and polymers are emulsified to form concentric layers, resulting in polymer lipoplexes and properties similar to poly lactic*‐co*‐glycolic acid nanocarriers. The outer lipid–PEG‐conjugated layer enhanced in vivo stability, whereas the inner cationic lipid layer enabled the encapsulation of anionic drugs. This design allows the codelivery of multiple medications, making it suitable for applications such as multidrug‐resistant cancer therapy.

## 4 Overview of Biological Barriers and Overcomes Through Nanocarriers

**Figure 4:**
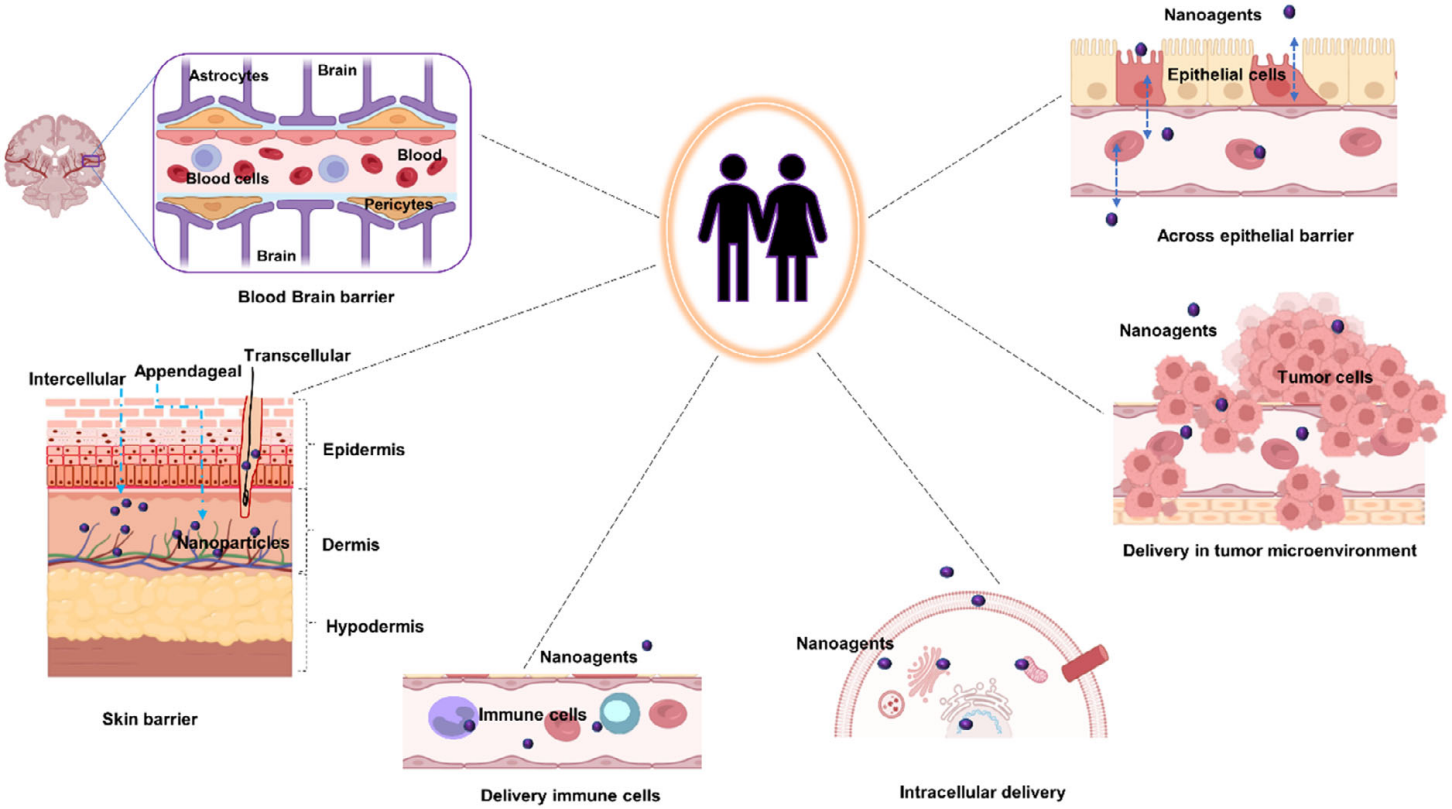
Biological barriers to effective drug delivery. This figure illustrates the major biological barriers that pose challenges to the effective delivery of therapeutics during drug development. These barriers include the BBB, skin layers, intercellular barrier, TME, and epithelial barrier. Each barrier presents unique challenges for drug delivery to target tissues, such as restricting the passage of molecules or altering drug distribution within the body. Created with BioRender.com.

The effective delivery of therapeutics to target tissues remains a major challenge in drug development. Biological barriers include the blood–brain barrier (BBB), skin layers, intercellular barrier, tumor microenvironment (TME), and epithelial barrier (**Figure** **4**). Nanocarriers offer a promising solution to overcome these barriers owing to their small size, high surface area‐to‐volume ratio, and customizable surface properties. Achieving successful biodistribution and drug delivery necessitates overcoming physiological and biological hurdles such as shear pressure, protein adsorption, and rapid clearance. These barriers undergo alterations in disease states, rendering them even more difficult to overcome using conventional one‐size‐fits‐all approaches. Changes in biological barriers are manifested at the systemic, microenvironmental, and cellular levels, exhibiting variability on a patient‐to‐patient basis. This variability makes it challenging to comprehensively identify and characterize these barriers. Nonetheless, understanding the biological barriers encountered by patients at a general level as well as on an individualized basis is crucial for developing optimal NP platforms. Achieving site‐specific drug delivery remains an elusive goal until the nanocarrier design effectively addresses the multitude of biological barriers encountered during drug administration. Although nanomedicine and nanodelivery systems represent burgeoning fields, overcoming these barriers and integrating unique design elements will pave the way for a new generation of nanotherapeutics.

### 4.1 Blood–Brain Barrier and Role of Carriers

**Figure 5:**
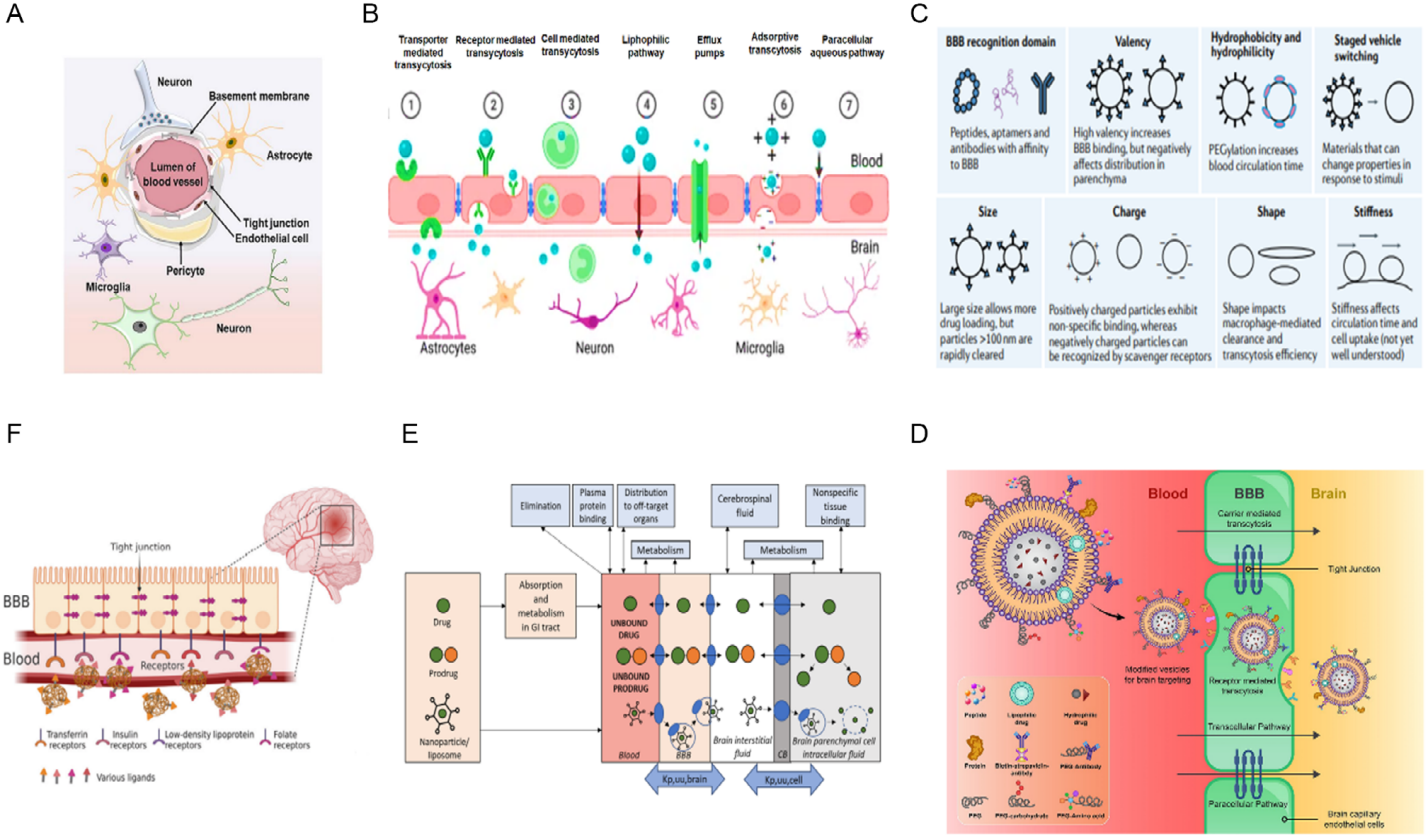
BBB regulation and targeted drug delivery across the barrier. A) Schematic representation of the BBB structure, showing brain capillary endothelial cells in association with astrocytes and pericytes.^[^ ^110^ ^]^ B) Diagram illustrating various mechanisms for crossing the BBB, such as passive diffusion, carrier‐mediated transport, receptor‐mediated transcytosis, and AMT.^[^ ^108^ ^]^ C) Influence of material properties on the adsorption, distribution, and clearance of DDSs following systemic administration, highlighting factors such as particle size, surface charge, and hydrophobicity.^[^ ^240^ ^]^ D) The various biomolecules and their conjugation to the vesicle as BBB‐targeted carriers, including antibodies, peptides, and small molecules The various biomolecules and their conjugation to the vesicle as BBB‐targeted carriers, including antibodies, peptides, and small molecules. Adapted with permission.^[^ ^241^ ^]^ Copyright 2022, Elsevier. E) Schematic depiction of the primary pharmacokinetic principles for transporter‐mediated drug delivery to the brain, including drug, prodrug, or nanocarrier transport across the BBB and brain parenchymal cellular barrier.^[^ ^242^ ^]^ F) Illustration of receptor‐mediated drug delivery utilizing ligand‐conjugated NPs targeted to the brain, showing the interaction between the ligand and specific receptors on the BBB.^[^ ^108^ ^]^ Figures are reproduced with permission.

The BBB is a highly specialized barrier that separates circulating blood from the extracellular fluid in the brain. Composed of endothelial cells, tight junctions, pericytes, and astrocytes, the BBB tightly regulates the passage of molecules into the central nervous system (CNS)^[^ ^49^, ^108^, ^109^ ^]^ (**Figure** **5**). The impermeability of the BBB poses a significant challenge in the treatment of neurological disorders because it restricts the delivery of therapeutic agents to the brain parenchyma.^[^ ^110^, ^111^ ^]^ The efficient delivery of nanocarriers to the CNS requires intricate mechanisms involving receptor‐mediated endocytosis by endothelial cells of the BBB, followed by exocytosis.^[^ ^49^ ^]^ The strategy for accessing brain tissue is receptor‐mediated transcytosis, which exploits receptors overexpressed in the BBB^[^ ^112^, ^113^ ^]^ (**Table** **3**). RMT triggers endocytosis via clathrin‐coated pits or caveolae, similar to adsorption‐mediated transcytosis (AMT). Upon internalization, nanoparticles navigate various intracellular pathways influenced by their size, charge, composition, and ligand conjugation. Commonly targeted receptors that facilitate RMT through the BBB include transferrin, lactoferrin, low‐density lipoprotein (LDL), and nicotinic acetylcholine. Moreover, insulin and insulin‐like growth factor receptors in the brain capillary endothelial cells offer intriguing prospects. This approach was explored in rhesus monkeys, where pegylated immune liposomes, conjugated with monoclonal antibodies against the human insulin receptor, demonstrated widespread β‐galactosidase expression in the primate brain.^[^ ^113^ ^]^

**Table 3:** Types of nanocarrier‐based DDSs targeting ApoE, transferrin, and lactoferrin for BBB.

Targeting Effector	Types	Size [nm]	Therapeutic cargo	In Vitro/In Vivo Results	References
Apolipoprotein E	Lipid nanocarriers; albumin nanocarriers	120–250	RSV	Enhanced brain targeting achieved through pulmonary administration in mice; increased permeability of RSV‐NPs observed across hCMEC/D3 monolayers; presence of ApoE‐NPs detected in brain capillary endothelial cells and neurons of mice	[116, 117]
Transferrin	Liposomes; lipid nanocarriers; polymeric nanocarrier; dendrimers	10–200	SQV, LOP, α‐M, DOX/PTX, pβ‐Gal	Tf‐liposomes improved brain delivery of α‐Mangostin in rats; higher LOP brain uptake and antinociceptive effects in the tail‐flick test in ICR (CD‐1) mice; strong antiglioma activity of DOX/PTX Tf‐NPs in mice	[269, 270, 271, 272, 273, 274]
Lactoferrin	Lipid nanocarriers; polymeric nanocarriers; dendrimers; liposomes	90–210	DOX, pβ‐Gal, DOX, NAP	Improved locomotor activity and reduced neuronal cell loss; increased bioavailability observed in mice; neuroprotection and memory improvements observed in AD mice model; enhanced permeation across BBB cell monolayer and inhibition of U87MG glioblastoma cell growth	[275, 276, 277, 278, 279, 280, 281]

The primary role of the LDL receptor is the clearance of LDL particles from the bloodstream through clathrin‐mediated endocytosis, a process crucial for regulating cholesterol levels and preventing atherosclerosis.^[^ ^114^, ^115^ ^]^ Interestingly, brain endothelial cells also express high LDL receptor levels, along with LDLR‐related protein 1 (LRP1) and very LDL receptors, making them potential targets for drug delivery across the BBB. Apolipoprotein E (ApoE), a soluble apolipoprotein that binds to LDL receptors, is a compelling target for facilitating BBB passage. By leveraging this interaction, researchers developed ApoE‐coated nanosystems to enhance drug delivery to the brain. Several studies have demonstrated the efficacy of ApoE‐coated nanocarriers in facilitating BBB traversal and improving the brain permeability of therapeutic agents.^[^ ^116^, ^117^ ^]^ Neves et al. utilized solid lipid nanocarriers functionalized with ApoE using biotin–avidin interactions to transport resveratrol, a bioactive compound with neurological benefits, across the BBB.^[^ ^118^ ^]^ Their findings revealed a significant increase in barrier transit for functionalized nanocarriers compared to that for nonfunctionalized nanocarriers. Similarly, Zensi et al. demonstrated the brain‐targeting capabilities of albumin nanocarriers bound to ApoE in intravenously injected mice. Coated NPs are specifically localized in the brain capillary endothelial cells and neurons, highlighting their potential for targeted drug delivery to the brain.^[^ ^119^ ^]^

The transferrin receptor (TfR), a membrane protein comprising two glycosylated subunits, binds to circulating transferrin and triggers endocytosis via clathrin‐coated pits. This is considered a promising approach for facilitating drug delivery across the BBB. TfR expression in brain endothelial cells is an alternative target for mediating BBB passage and drug delivery to the brain. Nanocarriers containing transferrin have emerged as a strategy to enhance the brain uptake of therapeutic agents. Numerous studies have demonstrated the effectiveness of transferrin‐functionalized nanocarriers in traversing the BBB and delivering drugs to the brain.^[^ ^120^, ^121^ ^]^ For example, cationic lipid nanocarriers incorporating transferrin exhibit successful transcytosis through brain microvascular endothelial cell monolayers in vitro, highlighting their potential for BBB penetration. Transferrin‐targeted nanocarriers have shown promise for brain cancer therapy.^[^ ^122^ ^]^ Liposomes conjugated with transferrin successfully targeted brain tumor cells in preclinical models, offering a potential strategy for precise drug delivery to tumor sites. Multifunctional nanocarriers combining magnetic, polymeric, and mesoporous silica components facilitate the delivery of chemotherapeutic drugs to brain cancer xenografts, resulting in significant tumor growth inhibition.

Lactoferrin and transferrin exhibit differences in their receptor‐binding properties that can be attributed to subtle structural variations in their lobes and interlobe linkers. Interestingly, lactoferrin demonstrates higher brain uptake than transferrin and other receptor‐targeting agents, offering potential advantages in enhancing drug delivery to the brain.^[^ ^123^ ^]^ Studies have demonstrated the use of lactoferrin‐functionalized nanocarriers in various therapeutic applications, including cancer, neurodegenerative diseases, and gene therapy. Nanocarriers coated with lactoferrin have been shown to enhance brain uptake and bioavailability in preclinical models, indicating their potential for targeted drug delivery to the brain. In the treatment of neurodegenerative diseases, such as AD, lactoferrin‐conjugated PEG‐PLGA NPs have shown promise and improved brain delivery.^[^ ^124^, ^125^ ^]^ Nanocarriers loaded with neuroprotective peptides or therapeutic molecules have demonstrated efficacy in rescuing neuronal function and improving memory in animal models of AD. The combination of LF‐mediated brain delivery with therapeutic interventions has yielded promising results in preclinical PD models. Nanocarriers loaded with therapeutic agents and functionalized with lactoferrin have been shown to rescue neuronal function and attenuate disease progression in animal models of PD.

### 4.2 Skin Barrier and Overcome by Nanocarriers

Nanoscale carrier systems have been extensively studied for their interactions with the skin and ability to enhance drug penetration. However, the increased production of engineered nanoscale materials necessitates a thorough safety evaluation. The success of drug delivery and risk assessment relies on the ability of these carriers to overcome the skin barrier and reach deeper tissue layers. Delivery through the skin can occur via intercellular, transcellular, or appendageal routes. Among these, hair follicles are particularly promising targets for NP carrier systems. The stratum corneum (SC) is the primary barrier to absorption. Despite their relatively small surface areas, skin appendages play a significant role and can potentially be targeted for directed delivery because of their depth.^[^ ^126^, ^127^ ^]^

Among various pathways involved in skin permeation, the transcellular route is the most direct and rapid. However, traversing this pathway poses challenges as the NPs encounter both lipophilic barriers, such as the cell membrane and lipid matrix, and hydrophobic barriers within the skin cells. The intercellular route has emerged as the primary pathway for nanocarrier penetration and involves diffusion between intercellular lipids. Successful navigation through this route requires careful consideration of the size and mechanical properties of the nanocarriers because flexibility is essential.^[^ ^127^, ^128^ ^]^ The follicular pathway is a commonly proposed method for highly rigid nanocarriers to penetrate the skin. This approach involves delivering medications directly through the hair follicles and glandular ducts. Nanocarriers that accumulate within the hair follicle canal exhibit depot activity, enabling sustained drug release. The size of the applied nanocarriers is crucial, as it affects the penetration depth and facilitates the selective targeting of specific compartments within the hair follicle. Toll et al. indicated that the use of smaller NPs leads to an increased penetration depth in terminal hair follicles. Similarly, studies by Roman et al. utilizing animal models demonstrated that ≈20 nm NPs penetrate deeper than polystyrene particles of ≈ 200 nm.^[^ ^129^ ^]^

Polymeric materials are promising and are mostly used as carrier agents for drug delivery because of their intrinsic biocompatibility and biodegradability.^[^ ^128^, ^130^ ^]^ Numerous studies on the penetration of drugs encapsulated in polymer‐based particles have highlighted the significant differences between conventional and nanoparticulate formulations. Liposomes comprise a closed bilayer of phospholipids and feature hydrophobic (lipid layer) and hydrophilic compartments (inner volume of the liposome). Their versatility in incorporating a wide variety of drugs, along with their inherent biocompatibility linked to natural phospholipids, makes them a major group of nanoparticulate carriers for cosmetic and therapeutic applications. Due to their larger size (≥50 nm) compared to skin openings, passive penetration of liposomes is hindered, necessitating a substantial driving force to propel them through the skin. Cevc and colleagues proposed a mechanism based on hydration gradient‐driven transport to elucidate the observed dermal penetration, wherein the differential activity of water on the two barrier sides facilitates aggregate migration. SLNs, composed of physiological solid lipids produced through high‐pressure homogenization, lead to enhanced drug permeation when applied to the skin. However, this increased drug absorption is attributed not to penetrating particles, but to the occlusive effect resulting from surface coverage.^[^ ^126^ ^]^ SLNs enhance the chemical stability of the encapsulated drugs by protecting them from hydrolysis and oxidation in a solid matrix. Additionally, SLNs contain physiological excipients that are generally recognized as safe, such as glyceryl behenate, glyceryl monostearate, glyceryl palmitostearate, trimyristin, tripalmitin, tristearin, or wax cetyl palmitate, making them acceptable from a cosmetic standpoint and well tolerated for topical application on the skin.^[^ ^131^, ^132^, ^133^ ^]^

Combining nanoemulsions with microneedles is a promising approach for delivering drugs that face challenges in diffusion across the skin barrier. Several examples highlight the effectiveness of this strategy, including the macroflux transdermal technology developed by Zosano (Mountain View, CA, United States) and the MicronJet developed by NanoPass Technologies (Haifa, Israel). These innovative technologies leverage the advantages of nanoemulsions, which enhance the solubility and bioavailability of drugs and the precise delivery capabilities of microneedles to facilitate medication administration through the skin. By overcoming the limitations of traditional drug delivery methods, such as poor skin permeability, these coupled systems offer a promising solution for improving the efficacy and efficiency of transdermal drug delivery.^[^ ^134^ ^]^ Nanoscale carrier systems have been extensively studied for their interaction with the skin and ability to enhance drug penetration. However, the increased production of engineered nanoscale materials necessitates a thorough safety evaluation. The success of drug delivery and risk assessment relies on the ability of these carriers to overcome the skin barrier and reach deeper tissue layers. Delivery through the skin can occur via intercellular, transcellular, or appendageal routes. Among these, hair follicles are particularly promising targets for NP carrier systems. The SC is the primary barrier to absorption. Despite their relatively small surface areas, skin appendages play a significant role and can potentially be targeted for directed delivery because of their depth.^[^ ^126^, ^127^ ^]^

Among various pathways involved in skin permeation, the transcellular route is the most direct and rapid. However, traversing this pathway poses challenges as the NPs encounter both lipophilic barriers, such as the cell membrane and lipid matrix, and hydrophobic barriers within the skin cells. The intercellular route has emerged as the primary pathway for nanocarrier penetration and involves diffusion between intercellular lipids. Successful navigation through this route requires careful consideration of the size and mechanical properties of the nanocarriers because flexibility is essential.^[^ ^127^, ^128^ ^]^ The follicular pathway is a commonly proposed method for highly rigid nanocarriers to penetrate the skin. This approach involves delivering medications directly through the hair follicles and glandular ducts. Nanocarriers that accumulate within the hair follicle canal exhibit depot activity, enabling sustained drug release. The size of the applied nanocarriers is crucial, as it affects the penetration depth and facilitates the selective targeting of specific compartments within the hair follicle. Toll et al. indicated that the use of smaller NPs leads to an increased penetration depth in terminal hair follicles. Similarly, studies by Roman et al. utilizing animal models demonstrated that ≈20 nm NPs penetrate deeper than polystyrene particles of ≈200 nm.^[^ ^129^ ^]^

Polymeric materials are promising and are mostly used as carrier agents for drug delivery because of their intrinsic biocompatibility and biodegradability.^[^ ^128^, ^130^ ^]^ Numerous studies on the penetration of drugs encapsulated in polymer‐based particles have highlighted the significant differences between conventional and nanoparticulate formulations. Liposomes comprise a closed bilayer of phospholipids and feature hydrophobic (lipid layer) and hydrophilic compartments (inner volume of the liposome). Their versatility in incorporating various drugs, along with their inherent biocompatibility linked to natural phospholipids, makes them a major group of nanoparticulate carriers for cosmetic and therapeutic applications. Due to their larger size (≥50 nm) compared to skin openings, passive penetration of liposomes is hindered, necessitating a substantial driving force to propel them through the skin. Cevc and co‐workers proposed a mechanism based on hydration gradient‐driven transport to elucidate the observed dermal penetration, wherein the differential activity of water on the two barrier sides facilitates aggregate migration. SLNs, composed of physiological solid lipids produced through high‐pressure homogenization, lead to enhanced drug permeation when applied to the skin. However, this increased drug absorption is attributed not to penetrating particles, but to the occlusive effect resulting from surface coverage.^[^ ^126^ ^]^ SLNs enhance the chemical stability of the encapsulated drugs by protecting them from hydrolysis and oxidation in a solid matrix. Additionally, SLNs contain physiological excipients that are generally recognized as safe, such as glyceryl behenate, glyceryl monostearate, glyceryl palmitostearate, trimyristin, tripalmitin, tristearin, or wax cetyl palmitate, making them acceptable from a cosmetic standpoint and well tolerated for topical application on the skin.^[^ ^131^, ^132^, ^133^ ^]^

Combining nanoemulsions with microneedles is a promising approach for delivering drugs that face challenges in diffusion across the skin barrier. Several examples highlight the effectiveness of this strategy, including the macroflux transdermal technology developed by Zosano (Mountain View, CA, United States) and the MicronJet developed by NanoPass Technologies (Haifa, Israel). These innovative technologies leverage the advantages of nanoemulsions, which enhance the solubility and bioavailability of drugs and the precise delivery capabilities of microneedles to facilitate medication administration through the skin. By overcoming the limitations of traditional drug delivery methods such as poor skin permeability, these coupled systems offer promising solutions for improving the efficacy and efficiency of transdermal drug delivery.^[^ ^134^ ^]^

### 4.3 Intercellular Barrier and Functional Role of Nanocarriers

Subcellular targeting is critical for achieving efficient and specific treatments for various diseases. This targeting is particularly crucial in conditions such as cancer, Alzheimer's disease, diabetes, infectious diseases, and autoimmune disorders, where the precise delivery of drugs to specific organelles within cells can significantly enhance therapeutic efficacy or alleviate symptoms.^[^ ^135^, ^136^ ^]^ However, the low pH and abundance of enzymes in the endosomes and lysosomes pose challenges. Thus, drug molecules that end up in these compartments may undergo degradation or be distributed nonspecifically, reducing their effectiveness.^[^ ^137^, ^138^ ^]^ The ability of nanomaterials to overcome these subcellular barriers has spurred the development of advanced drug delivery platforms. Various strategies for subcellular targeting often involve modulating the characteristics of nanomaterials such as size, charge, and surface composition. Hence, nanomaterials can be designed to interact with specific internalization pathways within cells, thereby avoiding entrapment within lysosomes and effectively reaching their target organelles. Nanomaterials can be engineered for specific pathways that lead to their direct delivery to targeted organelles, overcoming lysosomal degradation. This may involve modifying the surface properties to facilitate interactions with cellular receptors or intracellular transport machinery that drive them toward their target organelles.^[^ ^139^ ^]^

Nanomaterials offer various routes of administration, with oral delivery being the most convenient route. However, achieving efficient oral delivery poses significant challenges because nanomaterials must traverse several barriers along the GI tract. The GI tract serves as the primary barrier to successful nanomaterial absorption, particularly during drug degradation. Enzymes such as proteolytic enzymes in lysosomes, brush border peptidases in villi, and pancreatic proteases in the duodenal region can degrade drugs carried by nanocarriers.^[^ ^140^, ^141^ ^]^ Additionally, the gut bacterial flora contributes to mucin production, further complicating its absorption. Epithelial cells lining the GI tract form a physiological barrier due to the presence of tight junctions.^[^ ^142^ ^]^ These junctions restrict the paracellular transport of substances, including nanomaterials, across the epithelium.^[^ ^139^ ^]^ Overcoming these barriers is crucial to ensure the successful absorption and bioavailability of orally administered nanomaterials. Strategies to enhance oral delivery efficiency involve optimizing nanomaterial properties and formulation techniques to improve the stability, mucoadhesion, and permeability across epithelial barriers. The ability to manipulate the size, charge, and surface composition of nanomaterials plays a pivotal role in determining the cellular internalization pathways. Nanomaterials can effectively prevent lysosomal degradation and interact with specific organelles by adjusting these properties. This capability has spurred the development of innovative platforms aimed at enhancing the bioavailability of nanomaterials and optimizing their therapeutic efficacy.^[^ ^143^, ^144^, ^145^ ^]^

Several methods have been used to invade cells using nanocarriers. One strategy is to outfit nanocarriers with membrane‐lysing agents such as fusogenic lipids or cell‐penetrating peptides, which perforate the cell membrane and permit direct access to the cytosol.^[^ ^146^, ^147^ ^]^ Although this approach provides effective intracellular administration, intracellular component permeability increases the risk of cytotoxicity and immunogenicity. Alternatively, endocytosis, a basic cellular mechanism that carries molecules from the extracellular environment into cells, allows nanocarriers to enter the cells.^[^ ^147^, ^148^ ^]^

Targeting lysosomes with NPs is a promising strategy for enhancing the efficacy of cancer therapy while minimizing systemic toxicity. Lysosomes play a crucial role in the maintenance of cellular homeostasis and are integral to cellular waste management and recycling. Lysosomal function dysregulation has been implicated in various diseases, including cancer, making it an attractive target for therapeutic intervention. NPs offer an ideal platform for lysosomal targeting because of their unique physicochemical properties. Their small size, large surface area, and tunable surface chemistry enable efficient cellular uptake and intracellular trafficking. By engineering NPs to respond to lysosomal cues, such as low pH, drug release can be triggered selectively within lysosomes, enhancing therapeutic efficacy while reducing systemic toxicity. One strategy involves the incorporation of pH‐responsive moieties into NP formulations. When exposed to the acidic environment of lysosomes, these NPs undergo structural changes, leading to payload release. This pH‐triggered release mechanism ensures precise drug delivery to the lysosomes, where therapeutic agents can exert their effects more effectively. Using fluorescent BODIPY dyes for pH sensitivity, NPs can be conjugated to facilitate the real‐time monitoring of lysosomal targeting and drug release. By incorporating BODIPY dyes into NP formulations, researchers can track the intracellular fate of the NPs and validate lysosomal accumulation.^[^ ^149^, ^150^, ^151^, ^152^ ^]^ In cancer therapy, lysosomal targeting using nanocarriers is promising for acid‐activated tumor photodynamic therapy (PDT) and photoacoustic imaging. PDT involves the administration of photosensitizing agents that generate reactive oxygen species upon exposure to light, leading to localized cell death. By delivering photosensitizers specifically to lysosomes, nanocarriers can enhance the selectivity and efficacy of PDT against cancer cells, while minimizing damage to healthy tissues.^[^ ^153^, ^154^, ^155^ ^]^

Targeting the Golgi apparatus, a pivotal organelle in cellular trafficking and processing, is a promising strategy for therapeutic intervention and bioimaging.^[^ ^156^, ^157^ ^]^ Various nanocarriers have been engineered to precisely target the Golgi apparatus, each with a distinct targeting approach and carrying agent tailored for specific applications. Pdots, characterized by their exceptional optical properties, have emerged as valuable tools for long‐term near‐infrared fluorescent bioimaging. By incorporating trans‐Golgi network peptides into polymer dot formulations, these nanocarriers can actively target the Golgi apparatus. This targeted approach ensures the precise visualization of Golgi dynamics over extended periods, providing insights into intracellular processes with high spatiotemporal resolution. Polymer nanocarriers, however, offer a versatile platform for drug delivery, particularly in the context of treating tumor metastasis. Engineered to passively target the Golgi apparatus, polymer nanocarriers release therapeutic payloads upon accumulation in the tumor tissues. Celecoxib and brefeldin A, potent anticancer agents, are commonly encapsulated within polymer nanocarriers, effectively inhibiting tumor progression and metastasis while minimizing systemic side effects. Micelles are self‐assembling nanostructures composed of amphiphilic molecules that exhibit remarkable potential in antifibrotic therapies and the management of liver fibrosis. By actively targeting the Golgi apparatus through surface modification with chondroitin sulfate, micelles enable the precise delivery of therapeutic agents to cells, which are key players in fibrotic pathogenesis. Doxorubicin and retinoic acid encapsulated within micelles exert potent antifibrotic effects, mitigate fibrosis progression, and promote tissue regeneration.^[^ ^158^ ^]^

Polymer nanocarriers designed with triphenylphosphonium (TPP) for mitochondrial targeting effectively transport therapeutic drugs to mitochondria‐rich cells. The carrying agents used in this method are curcumin, 2,4‐dinitrophenol, lonidamine, and α‐tocopheryl succinate, each with unique therapeutic benefits. Curcumin, which is recognized for its strong antioxidant and antiinflammatory effects, is encapsulated in polymer nanocarriers to precisely target the mitochondria.^[^ ^159^, ^160^ ^]^ Using TPP's targeting capabilities of TPP, curcumin‐loaded NPs concentrate selectively within mitochondria‐rich cancer cells, exhibiting antiproliferative and proapoptotic effects. This focused method has the potential to improve the efficacy of curcumin‐based cancer treatments while reducing off‐target effects. Targeted delivery of these engineered nanoagents to mitochondria has the potential to treat mitochondrial dysfunction, which is associated with Alzheimer's disease. Indeed, mitochondria‐targeted therapy administered using polymer nanocarriers may reduce oxidative stress, alleviate neuronal damage, and improve cognitive performance in patients.^[^ ^161^, ^162^, ^163^ ^]^

The delivery of anticancer drugs to cancer cell nuclei using polymer nanocarriers is a promising strategy for improving cancer treatment outcomes. The targeting approach involves the utilization of fluorescein‐labeled nanocarriers with specific morphologies to ensure effective delivery to the nuclei. The carrying agent encapsulated within these nanocarriers is doxorubicin, a potent anticancer drug, which was encapsulated in these nanocarriers. By delivering doxorubicin directly to the nuclei of cancer cells, this approach enhances the effectiveness of the drug in inhibiting tumor growth and promoting cancer cell death. However, the morphology of nanocarriers can be optimized to enhance the nuclear targeting efficiency and ensure maximum therapeutic benefit.^[^ ^148^, ^164^, ^165^ ^]^

### 4.4 Tumor Microenvironment (TME) Barrier and Precise Drug Delivery

TMEs pose numerous obstacles to the effective delivery of therapeutic agents, thereby impeding treatment efficacy. The rapid tumor cell proliferation outpaces the capacity of neighboring blood vessels to supply oxygen and nutrients, resulting in a shortage of oxygen, known as hypoxia. Hypoxia triggers a cellular response primarily mediated by hypoxia‐inducible factors (HIF), including HIF1, HIF2, and HIF3.^[^ ^166^, ^167^ ^]^ These transcription factors regulate genes involved in various cellular processes such as glucose biosynthesis, angiogenesis, cell proliferation, migration, and immune response. In response to hypoxia, tumor cells undergo metabolic reprogramming, shifting from oxidative phosphorylation to aerobic glycolysis, a phenomenon known as the Warburg effect.^[^ ^168^ ^]^ Despite the presence of oxygen, this metabolic switch leads to increased lactate secretion into the extracellular environment, thereby contributing to acidification. The heightened proliferation and glycolytic metabolism of tumor cells result in reactive oxygen species (ROS) generation, which damages cellular components, including DNA, leading to genomic instability. Additionally, ROS stimulate antioxidant responses in tumor cells, further complicating the TME and influencing tumor progression. Immune cells within the TME exhibit contrasting functions in tumor growth.^[^ ^169^, ^170^ ^]^ While B and regulatory T cells contribute to an immunosuppressive microenvironment, natural killer cells and natural killer T cells stimulate antitumor immunity. However, the increased expression of granulocyte‐macrophage colony‐stimulating factor and vascular endothelial growth factor (VEGF) leads to myeloid‐derived suppressive cell formation in the bone marrow, which inhibits cytotoxic T cells and NK cells, thus correlating with poor prognosis.^[^ ^171^, ^172^ ^]^ The interplay between different components of the TME, including enhanced desmoplasia and hypoxia, facilitates the epithelial‐to‐mesenchymal transition of tumor cells, leading to cancer stem cell formation and metastatic spread. Matrix metalloproteinases play a critical role in epithelial mesenchymal transition by degrading extracellular matrix (ECM) components and promoting cancer stem cell development.^[^ ^173^ ^]^

**Figure 6:**
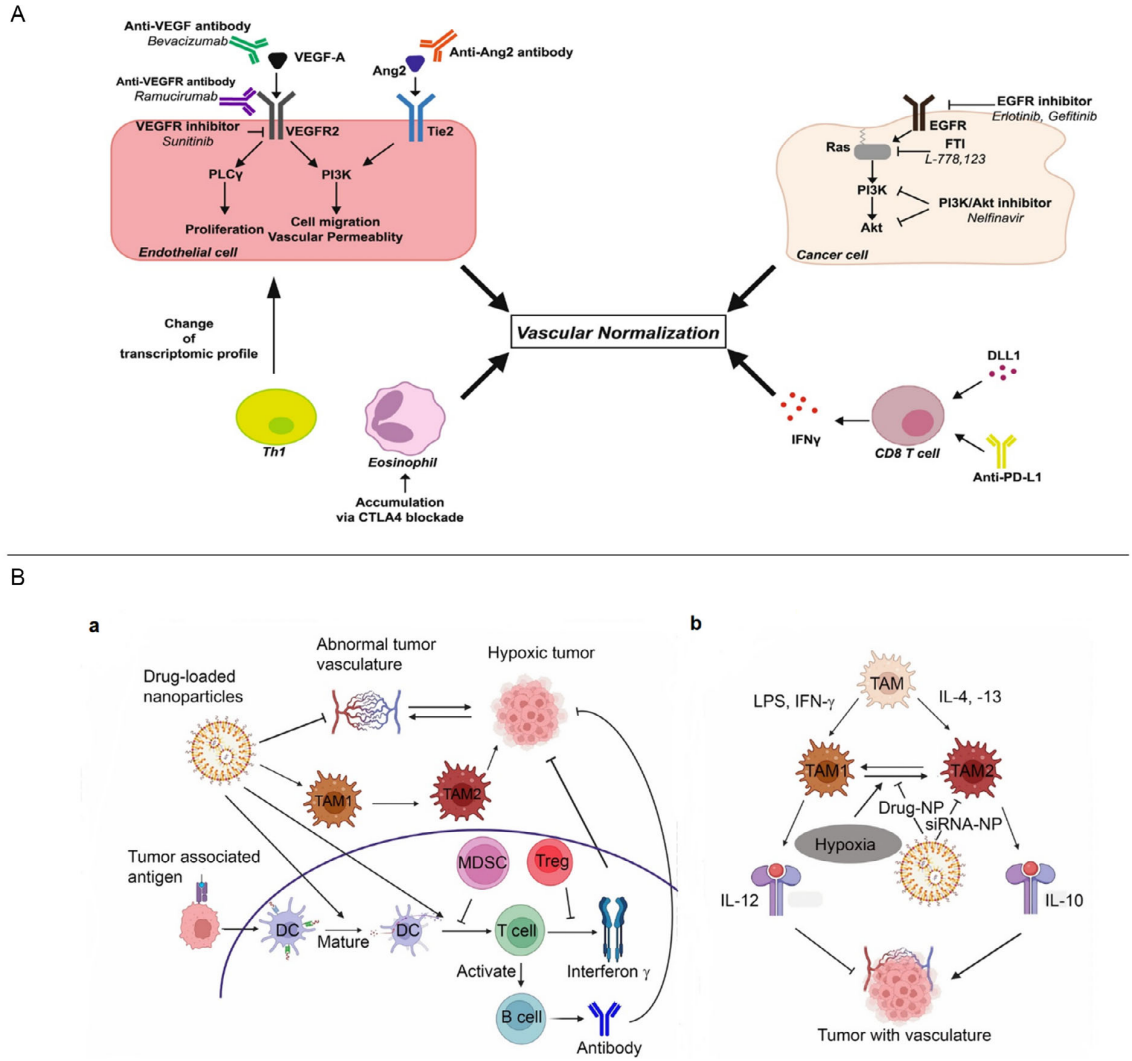
Vascular normalization mechanisms, associated signaling pathways, and the role of drug‐loaded NPs. A) Vascular normalization can be achieved through various strategies, including targeting VEGF signaling, Ang–Tie signaling, phospholipase C (PLC), phosphoinositide 3‐kinase (PI3K), angiopoietin 2 (Ang2), and epidermal growth factor receptor (EGFR) pathways in endothelial cells, as well as disrupting oncogenic signaling in cancer cells. Immune checkpoint blockade also induces vascular normalization by activating CD8^+^ or CD4^+^ T cells and promoting eosinophil accumulation. Key abbreviations: AAT (antiangiogenic therapy), IFP (interstitial fluid pressure), CAF (cancer‐associated fibroblast), ECM, VEGF (VEGFR (VEGF receptor).^[^ ^174^ ^]^ B) (a) Drug‐loaded NPs can suppress hypoxic tumor growth by targeting and reprogramming abnormal tumor vasculature. They also can convert M2 macrophages into M1 phenotypes, which reactivates the immune response, further inhibiting tumor growth. Additionally, these NPs can activate mature DCs, leading to the activation of B cells via T cells, and the production of antibodies that target and eliminate tumor cells. By stimulating these pathways, NPs can counteract the inhibitory effects of myeloid‐derived suppressor cells on mature DCs and the suppressive effects of regulatory T cells (Tregs) on interferon‐gamma (INF‐γ), ultimately resulting in tumor cell death. (b) TAMs can inhibit tumor vasculature through M1 macrophages and interleukin‐12 (IL‐12). However, hypoxic conditions can induce the conversion of TAM1 into TAM2, which promotes tumor vasculature through interleukin‐10 (IL‐10). Drug‐loaded NPs can prevent this TAM1 to TAM2 conversion, thereby reducing tumor growth. Additionally, siRNA‐loaded NPs can specifically suppress TAM2 activity.^[^ ^243^ ^]^ Figures are Reproduced with permission.

The TME orchestrates the formation of new blood vessels driven by the production of VEGF‐A by various TME components. VEGF‐A attaches to endothelial cells via the VEGF receptors, thereby stimulating angiogenesis. However, this process often results in abnormal blood vessels with damaged or undefined basal cells, leading to leaky vasculature and irregular structures surrounding the tumor. Dysfunctional blood vessels impair the delivery of nutrients and oxygen to the TME, exacerbating hypoxia and hindering the distribution of chemotherapeutic agents within the tumor. Moreover, the unstable composition of blood vessels contributes to the abnormal production of cytokines involved in the inflammatory and coagulation processes within the TME. Interestingly, the disorganized vasculature of the TME offers an opportunity for nanomedicines to selectively target tumor cells. Nanomedicines can exploit the leaky vasculature to preferentially accumulate within the tumor through a phenomenon known as the enhanced permeability and retention effect. Additionally, the TME promotes the lymphatic vessels development through the secretion of VEGF‐D and VEGF‐C by cancer, stromal, and immune cells, a process termed tumor‐associated lymphangiogenesis. However, lymphatic vessel development within the TME is associated with poor prognosis, as it facilitates metastatic spread to distant organs (**Figure** **6**A).^[^ ^173^, ^174^ ^]^

The TME also exhibits significant heterogeneity in immune cell composition across different tumor types and stages of tumor development. This variability is driven by dynamic shifts and modifications occurring within the TME, resulting in the production of multiple activation factors such as chemokines and cytokines. TME involves the differentiation of monocytes into two major subsets of macrophages, namely M1‐type and M2‐type macrophages. Tumor‐associated macrophages (TAMs) typically exhibit the M2 phenotype and contribute to tumor progression by promoting metastasis, angiogenesis, and invasion.^[^ ^175^, ^176^ ^]^

**Figure 7:**
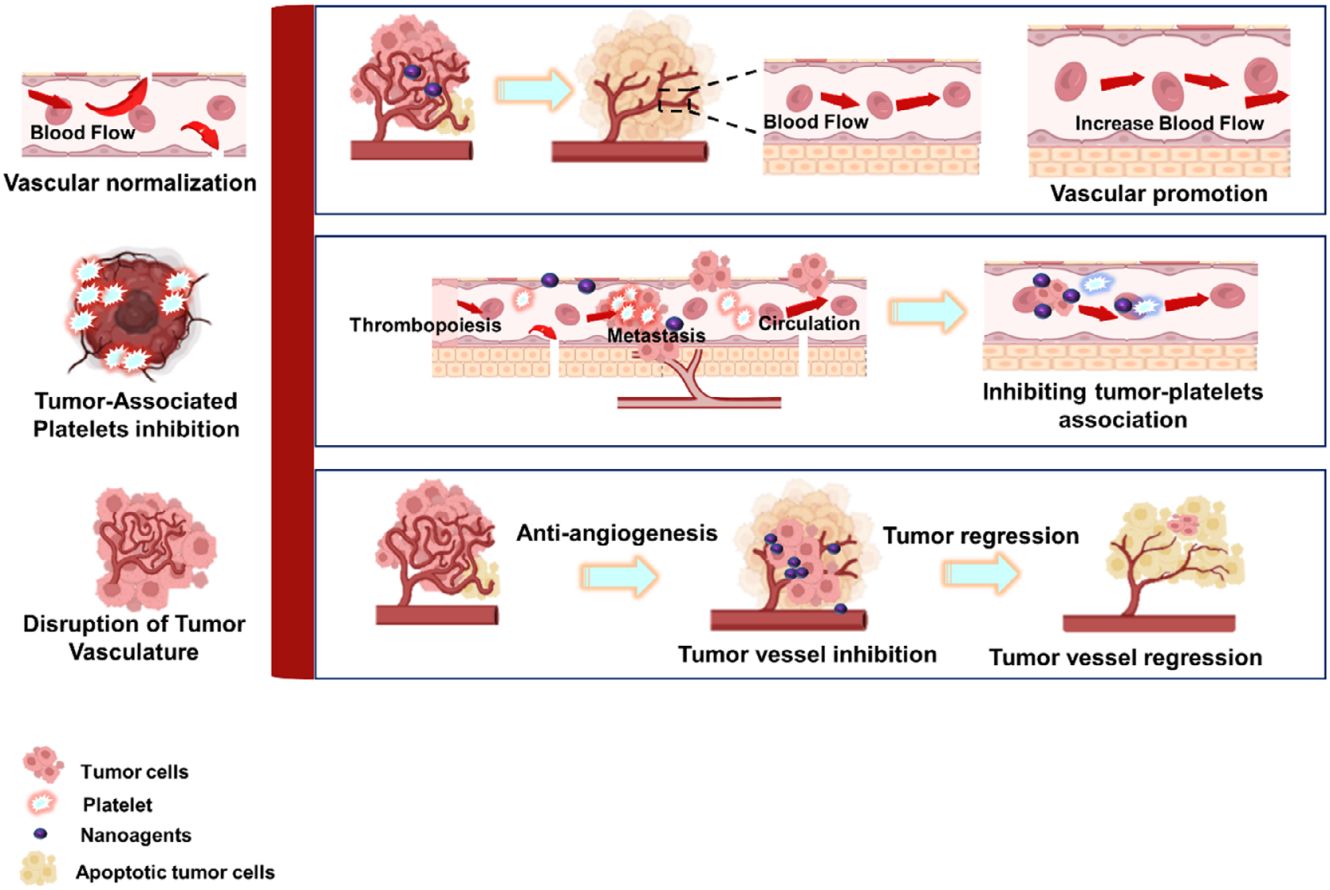
Strategies for overcoming abnormal vasculature barriers in the TME. This figure illustrates the three primary strategies developed to address the abnormal vasculature barriers encountered within the TME and facilitate the administration of nanoagents. Normalization and promotion: This involves interventions to normalize the tumor vasculature to improve blood flow and vessel integrity. Promoting the restoration of a more regular vascular network enhances the delivery of nanoagents to tumor tissues. Permeabilization or disruption: This strategy focuses on temporarily permeabilizing or disrupting the abnormal tumor vasculature to facilitate nanoagent penetration into tumor tissues. By creating transient openings in the vascular barrier, this approach allows for improved delivery of therapeutic agents to the TME. Tumor‐associated platelets inhibition: This strategy targets tumor‐associated platelets, which contribute to tumor growth and metastasis by promoting angiogenesis and thrombosis. By inhibiting the activity of these platelets, this approach aims to prevent abnormal vasculature formation and enhance the efficacy of nanoagent delivery to tumors. Created with BioRender.com.

Nanotechnology holds great promise in transforming cancer therapeutics by facilitating precise drug delivery, minimizing side effects, and improving patient outcomes. Nanosized carriers offer a platform for improving anticancer drug pharmacokinetics by encapsulating them within or conjugating them to the surface of NPs. The selective delivery of nanotherapeutic platforms relies primarily on passive targeting mechanisms, such as enhanced permeability and retention effects. To date, in the TME, three strategies–normalization and promotion, permeabilization or disruption, and tumor‐associated platelet inhibition–have been developed to overcome the abnormal vasculature barriers and administration of nanoagents (**Figure** **7**) (Figure 6Ba,b).^[^ ^177^, ^178^, ^179^ ^]^

**Figure 8:**
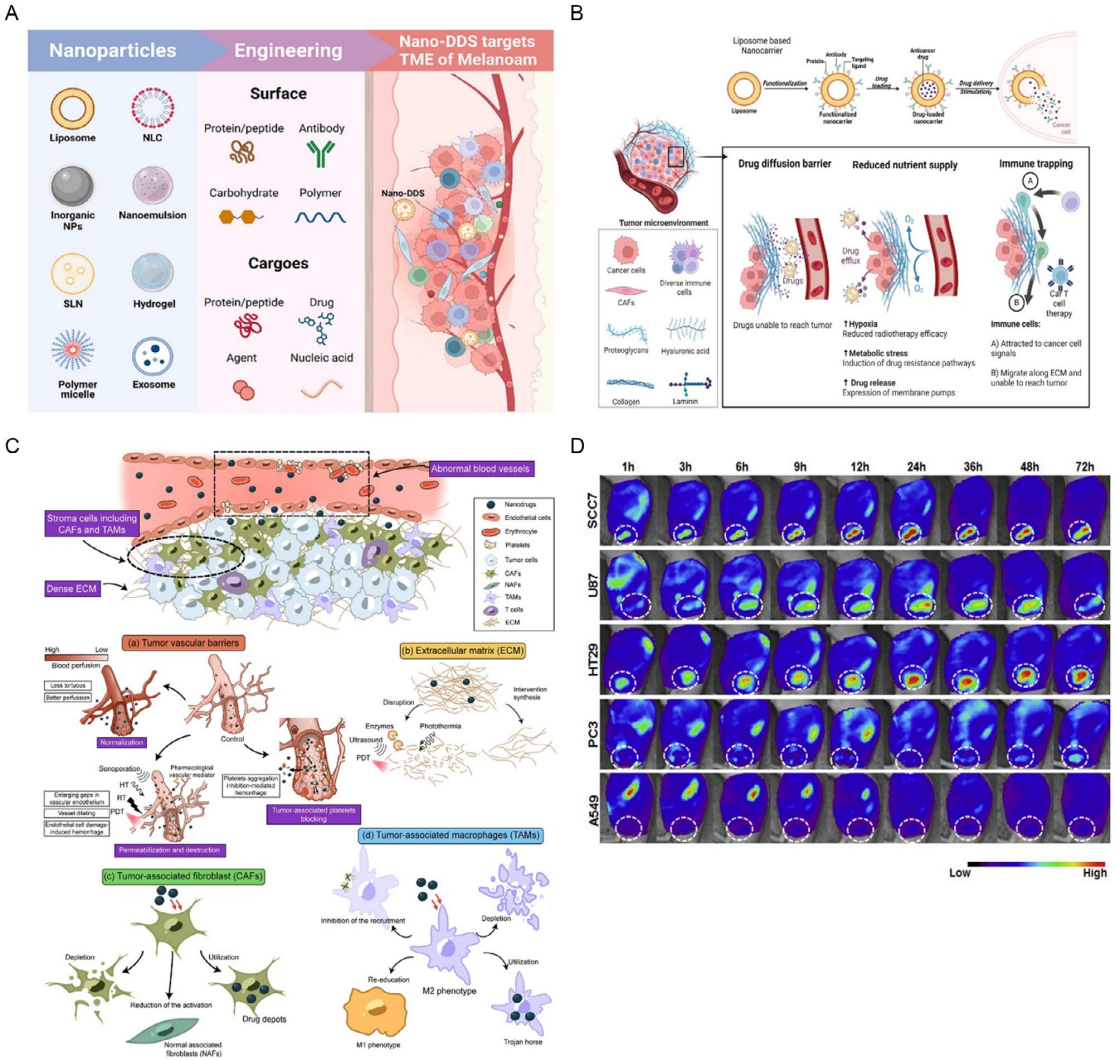
Nanocarriers for targeted DDS to the TME. A) Nano‐DDSs can selectively target TME of melanoma through modifications such as protein/peptide conjugation, antibody‐based targeting, carbohydrate surface decorating, or polymer coating. These carriers can then release their payloads into the TME, exerting antitumor effects. Adapted with permission. ^[^ ^244^ ^]^ Copyright 2021, Elsevier. B) Nano carrier‐based smart DDS overcomes the barriers in TME and releases drugs in cancer cells. Solid tumors induce high expression of ECM molecules (collagens, proteoglycans, hyaluronic acid, and laminins), which become complex and disordered, altering the characteristics of the TME. The ECM acts as a physical barrier, reducing the delivery of therapeutics, nutrients, and immune cells to solid tumors, leading to poorer prognosis. Created with BioRender.com.^[^ ^245^, ^246^ ^]^ C) The figure illustrates the biological barriers within the TME that affect nanodrug delivery, along with strategies for TME remodeling to enhance tumor penetration. (a) Techniques such as vasculature normalization, permeabilization, disruption, and blocking tumor‐associated platelets are employed to overcome barriers to transvascular transport. (b) Strategies for disrupting the ECM or inhibiting its synthesis are also depicted. Additionally, the regulation of (c) CAFs and (d) TAMs through depletion, reeducation, or utilization is shown to facilitate deeper infiltration of nanodrugs within the tumor stroma. Adapted with permission.^[^ ^177^ ^]^ Copyright 2017, Elsevier. Distribution of chitosan NPs (CNPs) in mice bearing transplanted tumors. Time‐dependent, noninvasive in vivo near‐infrared fluorescence (NIRF) imaging was performed on tumor‐bearing mice following the injection of NIRF dye‐labeled CNPs (200 μg μL^−1^ head). The distribution of CNPs across these various tumor models reflects the diverse characteristics of the TMEs associated with clinical tumors.^[^ ^247^ ^]^ Figures are Reproduced with permission.

TME defects, including compromised lymphatic drainage and increased permeability of the tumor vasculature, facilitate the accumulation of NPs, typically less than 200 nm in size, within the tumor tissue. Innovative NP‐based delivery systems are being explored to overcome physiological barriers and achieve targeted access to specific tumors (**Figure** **8**). Techniques such as mechanical particle deformation and synergistic approaches for delivering multiple chemotherapeutic agents such as paclitaxel and gemcitabine using mesoporous silica nanoconstructs have been investigated to enhance treatment efficacy.^[^ ^180^ ^]^ In a study investigating nucleic acid‐based therapy, PEG‐modified NPs and transferrin‐modified NPs were assessed for their effectiveness. These NPs were loaded with nucleic acids and evaluated in human prostate cancer and chronic myelogenous leukemia cell lines. The findings revealed that targeted NPs exhibited enhanced efficiency compared to untargeted particles, particularly when transfected into K562 leukemia cells.^[^ ^181^ ^]^

Another strategy involves the utilization of NPs to deliver immunostimulatory or immunomodulatory molecules, either alone or in combination with traditional chemotherapy or radiotherapy. These NPs can also act as adjuvants to enhance the efficacy of other immunotherapies. Another promising application of nanocarriers in cancer immunotherapy is their ability to capture antigens released from tumors following radiotherapy. By encapsulating these antigens, nanocarriers can enhance immune recognition and responses to cancer cells. Nanocarrier‐based vaccines are also being developed to elicit robust T‐cell responses. These vaccines employ various strategies, including the codelivery of antigens and adjuvants, dendritic cell (DC) activation by multiple antigens, and sustained antigen release over time. Such vaccines have the potential to enhance the immune response of the body to cancer cells. Furthermore, nanotechnology enables in situ vaccination approaches where artificial antigenpresenting cells or immune depots are placed near tumors to stimulate local immune responses.^[^ ^182^, ^183^, ^184^, ^185^ ^]^ These strategies aim to harness the ability of the immune system to recognize and eliminate cancer cells, ultimately leading to improved treatment outcomes.

### 4.5 Epithelial Barrier and Formulation of Nanocarrier

The epithelial barrier plays a crucial role in controlling drug absorption and in preventing the invasion of pathogenic bacteria into the host. Comprising epithelial cells, tight junctions, and a mucus layer, this barrier regulates nutrient absorption and interactions between the luminal contents and the underlying immune system. Goblet cells produce mucus that shields epithelial cells from bacterial interactions and physical damage caused by ingested food.^[^ ^186^ ^]^ Despite its vital role in intestinal protection, the mucus layer presents an initial challenge for nanocarriers. Many nanomaterials are trapped in the mucus gel layer,^[^ ^187^ ^]^ which hinders their ability to reach the intestinal epithelial cell layer. Once a nanocarrier penetrates the mucus layer, the primary obstacle is to cross the epithelial cells.^[^ ^188^, ^189^, ^190^ ^]^

Nanocarriers must traverse the epithelial cell layer to overcome the intestinal barrier. The GI tract presents several barriers to drug absorption, including the gastric barrier in the stomach and mucus and epithelial barriers. Strategies such as decorating nanocarriers with PEG chains, combining anionic and cationic charges, and utilizing self‐nanoemulsifying DDSs have proven to be effective in penetrating the mucosal barrier. Another approach involves decorating NPs with proteolytic enzymes, such as papain, to cleave mucoglycoprotein substructures, which have been shown to increase mucosal penetration.^[^ ^191^, ^192^, ^193^, ^194^, ^195^, ^196^, ^197^ ^]^ Many studies have reported endocytosis in human colorectal adenocarcinoma cells because the intestine represents the initial barrier to oral drug delivery via nanocarriers. Generally, positively charged NPs exhibit better uptake than negatively charged NPs, as demonstrated by 50 and 100 nm PS‐NP in Caco‐2 cells.^[^ ^198^, ^199^ ^]^ In contrast to epithelial cells, caveolae are the most prevalent endocytic structures in endothelial cells that line blood vessels. Consequently, numerous studies have examined caveolin‐mediated endocytosis of nanomaterials. For example, 20–100 nm BSA‐coated polymeric NPs in bovine lung microvascular endothelial cells are predominantly internalized through this pathway.^[^ ^192^, ^200^, ^201^, ^202^, ^203^ ^]^

## 5 Clinical Achievements of Nanocarriers‐Based DDSs

The nanopharmaceutical market has witnessed significant commercial success, with initial product offerings markedly enhancing the therapeutic effectiveness of numerous small‐molecule drugs.^[^ ^204^, ^205^, ^206^ ^]^ According to BCC Research, the global pharmaceutical market was valued at USD 209 billion in 2014. However, the landscape has evolved, and the COVID‐19 pandemic has significantly impacted market dynamics. In 2022, the global nanopharmaceutical market size is estimated to be USD 35 330 million, reflecting the effects of the pandemic. Nevertheless, the market is forecasted to undergo substantial readjustment, reaching USD 73 550 million by 2028, with a CAGR of 13.0% during the review period. This revision considers economic changes caused by the health crisis. Specifically, liposomes, which account for a significant portion of the global nanopharmaceutical market in 2021, are projected to reach USD million by 2028, growing at a revised percentage CAGR post‐COVID‐19 period. Similarly, the Cancer and Tumor segments experienced an altered percentage CAGR throughout the forecast period. Geographically, the market is predominantly concentrated in the USA (35% of the global market share), Asia (25%), and Europe (25%). Globally, over 200 companies are actively developing nanopharmaceuticals, approximately three‐quarters of which are start‐ups or small and medium enterprises (**Table** **4**). This diversity of players underscores the dynamic and competitive nature of the nanopharmaceutical industry.^[^ ^19^, ^26^, ^207^, ^208^, ^209^, ^210^ ^]^

**Table 4:** Commercially available and approved nanotherapeutic drugs in the USA, EU, and EMA.

Nanocarrier Typea), b)	Company	Product	Drug	Dosage form	Indication	Approval
Lipid‐based nanocarriers	Johnson & Johnson	Doxil	Doxorubicin (adriamycin)	Suspension (iv)	Metastatic ovarian cancer, HIV‐associated Kaposi's sarcoma (KS)	FDA (1995), EMA (1996)
	Mylan Pharmaceuticals Pvt Ltd	AmBisome	Amphotericin B	Suspension (iv)	Fungal infections, cryptococcal meningitis, and visceral	USA (1997)
	Endo Pharmaceuticals, Inc, Chadds Ford, PA	DepoDur	Morphine	Suspension (epidural)	Pain relief	USA (2004)
	The Pacira	Exparel	Bupivacaine	Suspension for injection (im)	Postsurgical analgesia	USA (2011)
	Merrimack	MM‐398	Irinotecan/ONIVYDE	Suspension (iv)	Metastatic adenocarcinoma of the pancreas with 5‐fluorouracil and leucovorin	USA (2015)
	Xellia Pharmaceuticals ApS, Copenhagen, Denmark	Amphotec	Amphotericin B	Suspension (iv)	Fungal infections, cryptococcal meningitis, and visceral leishmaniasis	USA (1996)
	Sun Pharma Global FZE	Lipodox	Doxorubicin hydrochloride	Intravenous (IV) infusion	Metastatic ovarian cancer, HIV‐associated KS	FDA (2013)
	Galen Ltd.	DaunoXome	Daunorubicin	Suspension (iv)	Cancers and HIV‐associated KS	FDA, EMA (1996)
	Merrimack Pharmaceuticals	Onivyde	Irinotecan	Intravenous (IV) infusion	Metastatic pancreatic cancer	FDA (2015)
	Pacira Pharmaceuticals	DepoCyt	Cytarabine	Suspension (intrathecal)	Lymphomatous meningitis	EMA (2002), FDA (2007)
	Teva Pharmaceutical Industries Ltd.	Myocet	Doxorubicin hydrochloride	Suspension (iv)	Breast cancer	EMA (2000)
	Janssen Pharmaceuticals	Caelyx	Doxorubicin	Suspension (iv)	Breast cancer, ovarian cancer, HIV‐associated KS	EMA (1996)
	Takeda France SAS	Mepact	Mifamurtide	Suspension (iv)	Osteogenic sarcoma	EMA (2009)
	Talon Therapeutics	Marqibo	Vincristine	Suspension	Philadelphia chromosome‐negative chronic myelogenous leukemia in adult patients	FDA (2012)
	Lipusu	Paclitaxel	Paclitaxel	Powder for suspension for infusion	Breast cancer, nonsmall‐cell lung cancer (NSCLC)	FDA (2016)
	Alnylam	Onpattro	Patisiran	Suspension (iv)	Hereditary transthyretin (TTR) mediated amyloidosis	FDA & EMA (2018)
	NeXstar Pharmaceuticals	AmBisome	Amphotericin B	Suspension (iv)	Antifungal drug	EMA (1990), FDA (1997)
	Jazz Pharmaceutics	Vyxeos	Daunorubicin and cytarabine	Suspension (iv)	Acute myeloid leukemia	FDA (2017), EMA (2018)
	Defiante Farmaceutica	Abelcet	Amphotericin B	Suspension (iv)	Antifungal drug	FDA (1995)
	SkyePharma	DepoDur	Liposomal morphine sulfate	Suspension	Postoperative analgesia	FDA (2004), EMA (2006)
	Chiesi	Curosurf	Poractant alfa	Suspension	Respiratory distress syndrome (RDS)	FDA (1999)
	Bayer Pharma	Zevalin	^90^Y‐ibritumomab tiuxetan	Solution (iv)	Lymphoma	FDA (2002), EMA (2004)
	Crucell Berna Biotech	Inflexal	Inactivated influenza virus vaccine	–	Prevents influenza infection	EMA (1997)
	Pfizer Pharmaceuticals	Pfizer‐BioNTech Vaccine	mRNA vaccine	–	Prevents COVID‐19 infection	FDA (2020)
	ModernaTX Inc.	Moderna COVID‐19 Vaccine	mRNA vaccine	–	Prevents COVID‐19 infection	FDA (2020)
	QLT Phototherapeutics	Visudyne	Photosensitizer (PS), Benzoporphyrin	–	Choroidal neovascularization caused by wet age‐related macular degeneration	FDA & EMA (2000)
	UCB Inc.	Cimzia	Certolizumab pegol	Subcutaneous injection	Rheumatoid arthritis, Crohn's disease, Psoriatic arthritis, and ankylosing spondylitis	FDA (2008), EMA (2009)
Polymer‐based nanocarriers	UCB	Apealea	Paclitaxel	Powder for suspension for infusion	Ovarian cancer, peritoneal cancer, fallopian tube cancer	EMA (2018)
	Enzon Pharmaceuticals Inc.	Adagen	Adenosine deaminase (ADA)	–	Adenosine deaminase (ADA)‐severe combined immunodeficiency disorder	FDA (1990)
	Amgen, Inc.	Neulasta	Filgrastim	Solution (sc)	Febrile neutropenia, consequent infections arising due to lack of neutrophils	FDA (2002)
	Enzon Pharmaceuticals Inc.	Oncaspar	Pegaspargase	Intravenous infusion or an intramuscular injection.	Acute lymphoblastic leukemia, chronic myelogenous leukemia	FDA (1994), EMA (2016)
	Lupin Ltd.	Genexol‐PM	Paclitaxel	Powder for suspension for infusion	Breast cancer	FDA (2007)
	Genentech USA, Inc	Pegasys	Pegylated interferon alfa‐2a	Suspension for injection	Hepatitis C, hepatitis B	FDA, EMA (2002)
	Fresenius Kabi	Diprivan	Propofol	Emulsion (iv)	A sedative‐hypnotic agent used in surgery to induce relaxation before and during general anesthesia	FDA (1989), EMA (2001)
	Pfizer Pharmaceuticals	Somavert	Pegvisomant (PEG‐HGH antagonist)a	Powder and solvent for solution for injection (sc)	Acromegaly	EMA (2002), FDA (2003)
	Pfizer Pharmaceuticals	Macugen	Pegatinib sodium	–	Choroidal neovascularization caused by wet age‐related macular degeneration	FDA (2004)
	Vifor, (Roche Pharma)	Mircera	Epoetin β (EPO)	Solution (iv/sc)	Anemia	EMA (2007), FDA (2018)
	Merk & Co. Inc.	PegIntron	Peginterferon alpha‐2b	Powder and solvent for solution for injection (sc)	Hepatitis C	EMA (2000), FDA (2001)
	Savient Pharmaceuticals	Krystexxa	Pegloticase	Intravenous infusion	Refractory chronic gout	FDA (2010)
	Biogene	Plegridy	Recombinant IFN‐β	Solution	Relapsing‐remitting multiple sclerosis (RRMS) in adult patients	FDA (2014)
	Baxalta US Inc.	Adynovate	Coagulation factor VIII	Intravenous (IV) injection	Hemophilia A	FDA (2015)
	Teva Pharmaceutical Industries Ltd.	Copaxone/FOGA	Glatiramer acetate	–	Multiple sclerosis (MS)	FDA (1996), EMA (2016)
	Tolmar Pharmaceuticals Inc.	Eligard	Leuprolide acetate	–	Prostate cancer	FDA (2002)
	Genzyme Ltd, Oxford, UK)/(Genzyme Ireland Ltd, Co, Waterford City, IE	Renagel/Renvela	Sevelamer carbonate	Tablet	Hyperphosphatemia caused by chronic kidney disease (CKD)	FDA (2000)
	Genzyme	Renagel/Renvela	Sevelamer HCL	Tablet	Hyperphosphatemia caused by CKD	EMA (2007)
	Allergan	Restasis	Cyclosporine	Emulsion	Chronic dry eye	FDA (2003)
	NovoNordisk	Rebinyn	Rebinyn Coagulation Factor IX	Bolus injection	Hemophilia B	FDA (2017)
	Novavax, Inc.	Estrasorb	Estradiol	Suspension	Moderate vasomotor symptoms due to menopause	FDA (2003)
	Flexion Therapeutics	Zilretta	Triamcinolone acetonide	Injectable suspension	Knee osteoarthritis	FDA (2017)
Nanoemulsion	Fresenius Kabi	Diprivan	Propofol	Suspension (iv)	General anesthesia	USA (1989)
	NOVARTIS PHARMACEUTICALS CANADA INC	Durezol	Difluprednate	Emulsion	Eye inflammation and uveitis	USA (2008)
	Allergan, Inc.,	Restasis	Cyclosporine A	Emulsion	Dry eye syndrome	USA (2003)
	Aesica Queenborough Ltd, Queenborough, UK	Ritonavir	Norvir	HIV infections	Soft capsules	FDA (1996)
Dendrimer‐based nanocarriers	Starpharma	VivaGel BV	Astodrimer sodium	–	Anti‐infective for prevention of recurrent bacterial vaginosis (BV)	FDA (2015)

a) Abbreviations: EMA, European Medicines Agency; IV, intravenous; FDA, Food and Drug Administration;

b) Sources: The data in this modified table were adapted from Alam,^[^ ^282^ ^]^ Ragelle et al.^[^ ^34^ ^]^ and Hafner et al.^[^ ^19^ ^]^ and reused with permission.

The anticipated nanopharmaceutical market growth is driven by the emergence of a new generation of nanocarrier‐based systems. These advancements include the delivery of biotherapeutics, particularly nucleic acids, polymeric formulations, targeted nanocarriers, and metal‐based nanocarriers that act as therapeutic agents under external stimuli. Additionally, existing delivery technologies, such as nanocarrier‐based albumin‐bound technology, are being repurposed for other classes of drugs. For example, there is an ongoing investigation into the use of this technology for rapamycin delivery. Moreover, the development of nanocarrier‐based DDSs is becoming increasingly prevalent and is expected to substantially contribute to the growth of the nanopharmaceutical market.

The primary advantages of first‐generation nanopharmaceuticals over their parent drugs are twofold: 1) improvement of the pharmacokinetic profile, leading to reduced toxicity; and 2) enhancement of pharmaceutical efficacy or dosing through drug reformulation.^[^ ^211^ ^]^ One notable example of a first‐generation nanopharmaceutical is Doxil, which became the first nanoscale drug carrier to receive FDA approval in the United States, just 5 years after its initial development. Clinical studies have demonstrated that Doxil exhibits superior therapeutic efficacy in treating ovarian cancers and AIDS‐related Kaposi's sarcoma compared to standard therapies.^[^ ^212^, ^213^, ^214^, ^215^ ^]^ It also demonstrated equivalent efficacy in treating multiple myeloma and metastatic breast cancer. However, despite its overall better tolerability, Doxil infusion has been associated with skin toxicity, specifically Palmar Plantar erythrodysesthesia or hand–foot syndrome. This adverse effect was attributed to the slow extravasation of PEGylated liposomes into the skin due to their prolonged circulation time. Following the approval of Doxil, several other liposomal and lipid‐based delivery systems have been developed and gained approval, including lipid nanocarriers and nanoemulsions. Moreover, the pharmaceutical industry has shifted its focus beyond small‐molecule compounds to the development and manufacture of various biotherapeutics, including recombinant proteins, monoclonal antibodies, antibody fragments, and nucleic acids.

## 6 Challenges Associated with Nanocarriers‐Based DDSs

Nanocarriers offer several benefits as DDSs, including precise drug delivery, surmounting biological barriers, and reducing adverse effects associated with traditional drug delivery methods. However, despite their potential advantages, NPs present certain obstacles and constraints, notably concerning their potential toxicity and intricate manufacturing processes. One notable concern arising from the increased use of NPs is the risk of nano‐toxicity.^[^ ^216^, ^217^, ^218^ ^]^ Elevated intracellular ROS levels have been associated with the accumulation of diverse nanocarriers, resulting in oxidative stress and adverse effects on various organ systems, including the respiratory, skin, liver, kidney, reproductive, embryonic, central nervous, and immune systems. These adverse effects are frequently attributed to factors such as nanocarrier dimensions, morphology, surface characteristics, method of administration, and interactions with biological barriers.

To overcome these challenges and enhance the performance of nanocarrier‐based DDSs, numerous areas of research and development warrant prioritization. Enhancing nanocarrier stability in biological settings is crucial for ensuring effective drug delivery. Strategies to bolster stability may involve surface modifications, formulation enhancements, and utilization of stabilizing agents. Additionally, achieving an efficient distribution of bioactive compounds to target tissues or cells is essential for optimizing therapeutic outcomes. Research endeavors should concentrate on enhancing drug absorption capabilities, attaining site‐specific targeting, and augmenting drug transport to desired locations within the body.^[^ ^219^, ^220^ ^]^ The development of nanocarrier systems capable of controlling drug release kinetics can aid in optimizing drug delivery and minimizing off‐target effects. This may entail the design of stimuli‐responsive nanocarriers or the incorporation of release‐modulating components. Overcoming biological barriers, such as the BBB or mucosal barrier, is pivotal for successful drug delivery. Strategies aimed at enhancing NP penetration and retention at target sites are critical for enhancing therapeutic outcomes.

Implementing advanced safety measures for nanocarrier characterization, identification, and toxicity assessment is indispensable for ensuring patient safety. This may necessitate the establishment of standardized protocols, robust quality control systems, and a comprehensive preclinical evaluation of nanocarrier formulations. The commercial development of nanocarrier‐based DDSs presents distinctive challenges in terms of manufacturing, characterization, and regulatory approval. Scaling up nanocarrier production to meet clinical demands while upholding batch‐to‐batch reproducibility and quality control standards necessitates meticulous optimization and coordination among the research, development, and manufacturing teams. Furthermore, ensuring adherence to regulatory requirements and addressing safety concerns are pivotal steps in the translation of nanocarrier‐based DDSs from laboratory to clinical settings.

## 7 Future Prospectives of Artificial Intelligence in Biomedicine Research

The integration of AI systems has a profound impact on medicine and medical science. This impact includes the use of machine learning (ML), deep learning, and quantitative structure–activity relationship modeling in nanoscience, providing a comprehensive predictive assessment of nanomaterials, chemicals, and toxicological effects on biological systems.^[^ ^221^, ^222^, ^223^ ^]^ These technologies can analyze complex medical data, such as imaging scans, genetic sequences, and patient records, to assist clinicians in making accurate diagnoses, predicting treatment outcomes, and identifying optimal treatment strategies. Clinical decision‐support tools enable clinicians to swiftly provide access to highly relevant information, enabling informed decisions regarding patient treatment, medications, and other care needs. Additionally, AI tools analyze various medical images, such as CT scans, X‐rays, and magnetic resonance imaging, aiding radiologists in achieving precise diagnoses by detecting lesions or other markers.^[^ ^224^, ^225^, ^226^ ^]^ AI is at the forefront of driving biomedical research by enabling data‐driven insights, expediting the drug discovery process (**Table** **5**), and fostering personalized healthcare approaches. Its applications span various domains, including clinical decision support and medical image analysis, in healthcare settings.

**Table 5:** AI model tools used for drug discovery, the advantages, and limitations.

Aspect	Details	Example	Advantages	Limitations
Molecular property prediction	AI models predict molecular properties like solubility, toxicity, and binding affinity.	DeepChem	Supports various deep learning models, large‐scale datasets, and integration with TensorFlow/PyTorch	Potential overfitting, high computational requirements for large datasets
Structure‐based drug design	AI models analyze 3D structures of proteins and ligands to predict interactions and design drugs.	Schrödinger's Maestro (FEP+)	High accuracy in predicting binding affinities, integrates with virtual screening workflows	Expensive licensing, requires specialized knowledge to operate effectively
Virtual screening	High‐throughput screening using AI to identify potential drug candidates from large libraries.	Atomwise (AtomNet)	Scalable for large datasets, uses CNNs for structure analysis	Requires high‐quality structural data, potential bias if trained on limited datasets
Target identification	AI identifies and validates novel therapeutic targets using omics data and ML.	Insilico Medicine's PandaOmics	Integrates multiomics data, prioritizes targets with AI, user‐friendly interface	Limited by the quality and diversity of input data, potential for false positives
Chemical synthesis planning	AI predicts synthetic pathways for drug candidates, optimizing chemical synthesis routes.	Molecule.one	Real‐time synthesis planning, high accuracy in pathway prediction	Challenges with complex molecules, reliance on existing chemical reaction databases
Lead optimization	AI models optimize drug leads by predicting modifications that enhance efficacy and reduce toxicity.	Exscientia's Centaur Chemist	Combines human expertise with AI, multiobjective optimization for drug properties	Requires iterative feedback, potential human bias in the loop
Data integration and analysis	AI integrates and analyzes heterogeneous biological data to discover drug candidates.	BioSymetrics’ Augusta	Scalable data integration, automated pipelines for ML	Data preprocessing challenges, potential for overfitting if data is not diverse
Natural language processing (NLP)	NLP models process scientific literature and unstructured data to identify drug targets and mechanisms.	IBM Watson for Drug Discovery	Processes large volumes of unstructured data, integrates with research workflows	High cost, requires significant training data, potential for misinterpretation of complex texts
Protein–ligand interaction modeling	AI visualizes and analyzes interactions between proteins and ligands for drug design.	Cresset's Flare	High‐quality 3D visualization, advanced modeling tools	Complex software with a steep learning curve, potential for high computational demands
Multiomics data integration	AI integrates data from various omics platforms (genomics, proteomics) for comprehensive drug discovery.	BenevolentAI	Advanced data integration, deep learning models for accurate predictions	Limited by the diversity and quality of omics data, high computational costs
Gene editing and RNA‐based therapeutics	AI models design and optimize CRISPR‐based gene editing and RNA interference therapies.	DeepChem (with CRISPR libraries)	Personalized medicine, gene‐specific therapy options	Ethical concerns, potential off‐target effects, high technical complexity

### 7.1 Convergence of AI with Nanoscience and Modeling of Nanocarriers for Drug Delivery

**Figure 9:**
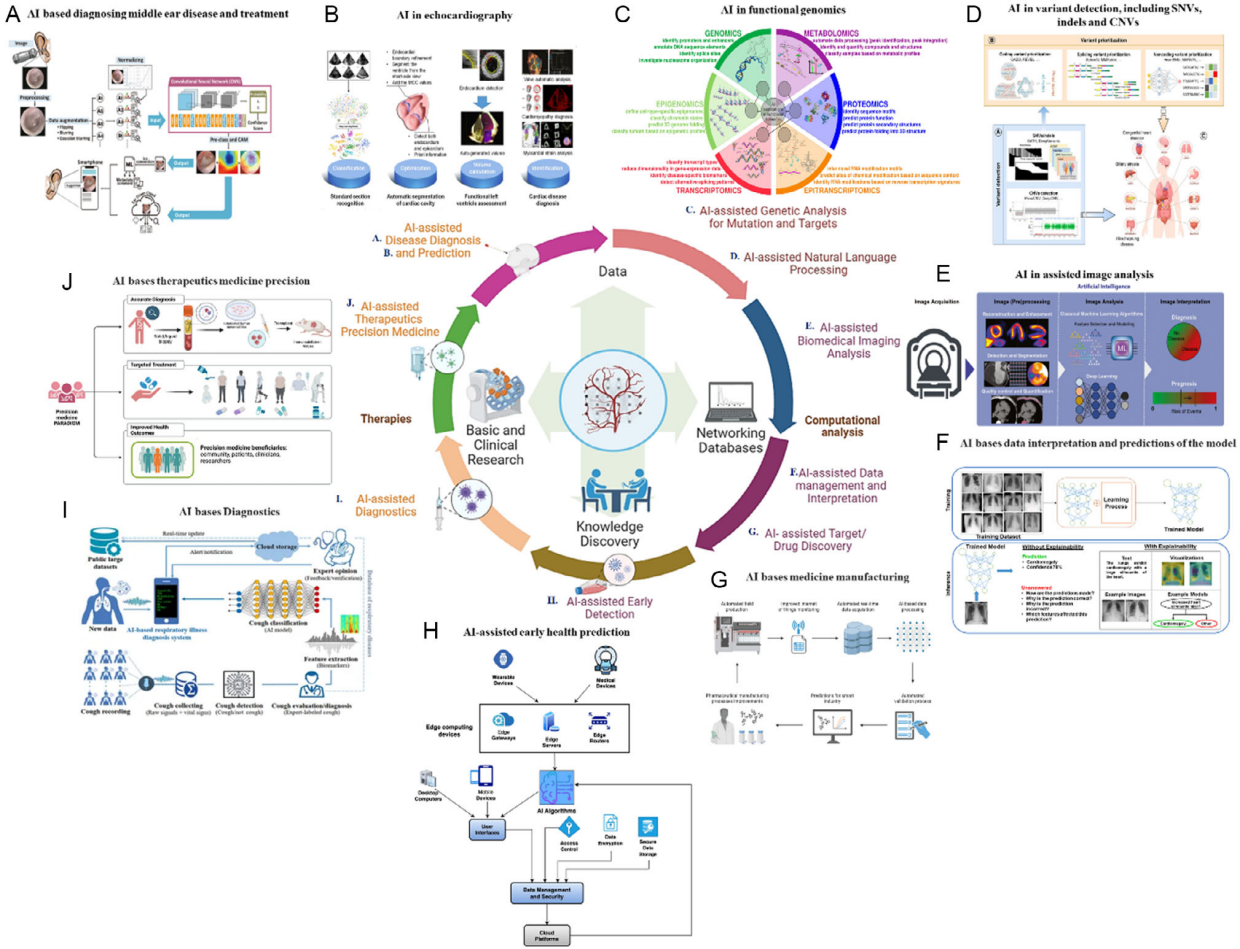
Role of AI in biomedical research. This figure highlights the pivotal role of AI in driving biomedical research and revolutionizing healthcare. AI significantly advances biomedical research by unlocking new insights, accelerating discovery processes, and facilitating personalized healthcare approaches. Key applications include AI‐based disease diagnostics and prediction: A) Developing a smartphone‐based computing program for diagnosing middle ear disease and providing medical suggestions. Adapted with permission.^[^ ^248^ ^]^ Copyright 2022, Elsevier. B) AI in echocardiography.^[^ ^249^ ^]^ C) AI‐assisted genetic analysis for mutation and target identification, particularly in functional genomics AI‐assisted genetic analysis for mutation and target identification, particularly in functional genomics.^[^ ^250^ ^]^ D) AI models for variant detection and prioritization of disease‐causing variants in different genomic regions.^[^ ^251^ ^]^ E) AI‐assisted biomedical imaging and processing, enhancing tasks such as preprocessing, image analysis, and interpretation, integrated with ML.^[^ ^252^ ^]^ F) AI‐based data interception and model prediction.^[^ ^253^ ^]^ G) AI‐assisted targeted drug delivery, Created with BioRender.com.^[^ ^231^ ^]^ H) AI‐based early heath prediction.^[^ ^254^ ^]^ I) AI‐assisted diagnostics, including AI‐based cough diagnosis systems that encompass every stage with data exchange capabilities for researchers and medical doctors.^[^ ^255^ ^]^ J) AI‐assisted therapeutics in precision medicine, particularly in evaluating cancer treatments.^[^ ^256^ ^]^ Figures are reproduced with permission. Created with BioRender.com.

AI has emerged as a cornerstone in the field of drug delivery modeling, offering profound insights into biological systems and revolutionizing the optimization of nanosystem designs^[^ ^227^, ^228^ ^]^ (**Figure** **9**A–J). In the context of drug delivery, the intricacies of the human body are simplified into distinct compartments delineated by biological barriers. These barriers, which are permeable through active or passive mechanisms, are critical considerations in drug transport. Passive diffusion, governed by the molecular properties of drugs, undergoes dynamic alterations upon exposure to physiological environments, enabling the predictive modeling of drug distribution complexes. In contrast, active diffusion involves energy‐activated transporter systems to facilitate drug transport across biological membranes. This intricate interplay between membrane transporters and drug molecules lends itself to computational modeling, which enables the analysis of various parameters.

Artificial neural networks (ANNs) have been harnessed to unravel the quantitative relationships between different formulations, processing variables, and the ensuing in vitro and in vivo characteristics. In nanotechnology, ANNs have been employed to characterize the structural properties of nanomaterials; estimate the effective dielectric constant of thin films; model the nanoprecipitation of insoluble drugs such as prednisolone; and characterize physiochemical properties such as water uptake, viscosity, and glass transition state of amorphous polymers.^[^ ^229^, ^230^ ^]^ Through meticulous modeling, predictive algorithms are formulated, enabling the accurate estimation of formulation properties based on input variables, such as material attributes and process parameters. This predictive capability facilitates the optimization of formulation properties tailored for specific applications, ensuring the efficacy and precision of drug delivery. Moreover, ANNs are invaluable tools for predicting the fundamental properties of polymers, including moisture content, viscosity, glass transition temperatures, and water‐uptake profiles. Such predictive models offer invaluable insights during the preformulation stage, guiding the development process toward optimal outcomes. The integration of AI modeling extends beyond formulation optimization to encompass the diverse facets of drug delivery. AI‐driven methodologies streamline various aspects of DDS development by predicting dissolution profiles to evaluate the transportability of drugs and identify suitable polymer composites.^[^ ^231^ ^]^

Accurately modeling nanoscale circumstances is a fundamental challenge in nanotechnology. Nanoscale data is typically interpreted through numerical simulations, as real optical images cannot be obtained at such high resolutions.^[^ ^232^, ^233^ ^]^ However, these simulations often involve complex processes that require the integration of multiple parameters, which can make the modeling process overwhelming. AI can be highly beneficial in this context, as it simplifies the modeling process, enabling more effective and straightforward interpretation of nanoscale data. Using sophisticated algorithms, AI can generate more accurate simulations, reduce the need for manual parameter adjustments, and enhance our overall understanding of nanoscale images.^[^ ^234^, ^235^ ^]^

**Figure 10:**
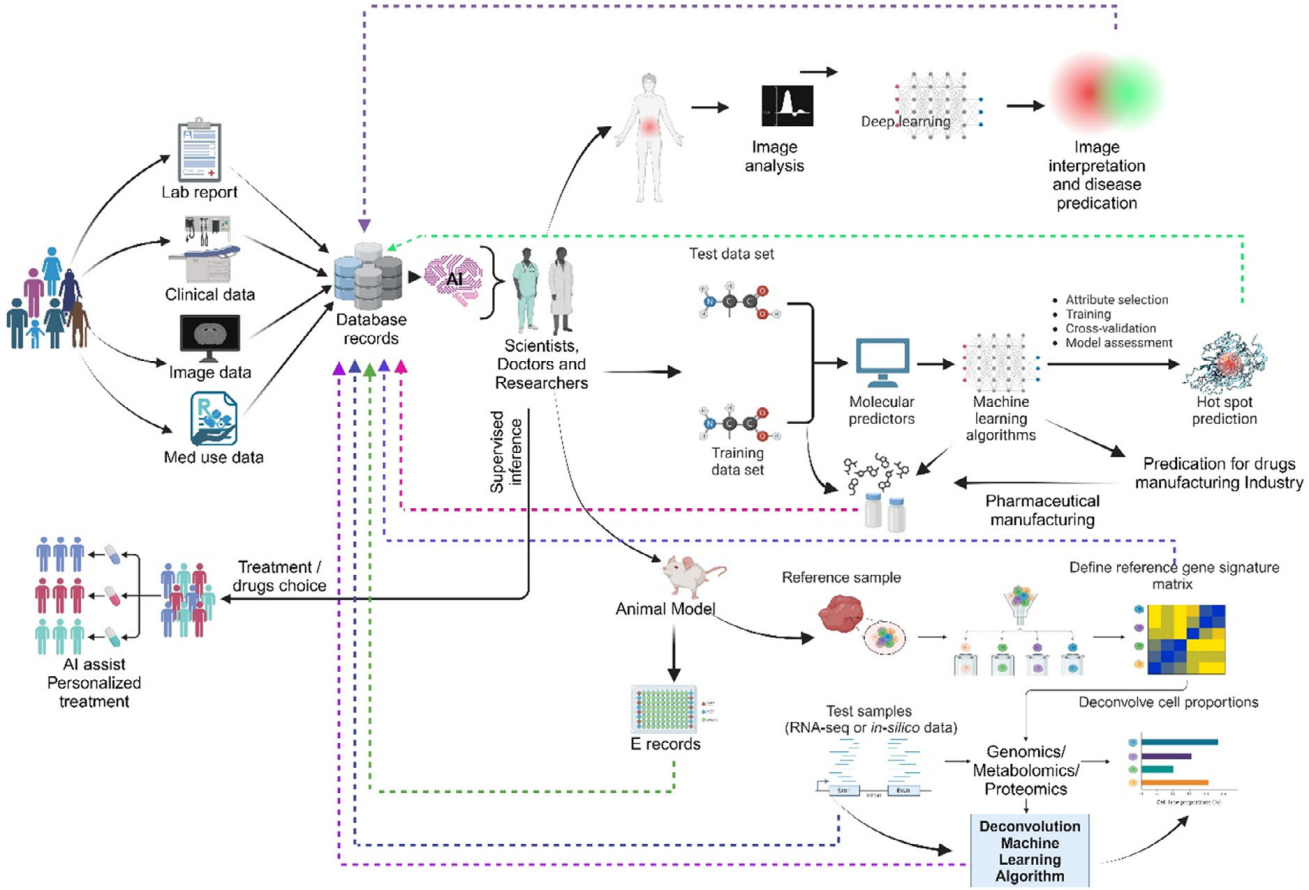
The diagram illustrates the role of AI integrated across various stages of biomedical research, highlighting its impact and challenges. AI‐driven workflows are increasingly explored in digital pathology, drug discovery, and development, showcasing significant potential in personalized care, clinical decision support systems, early disease detection, disease monitoring, and dynamic drug dosing. However, the successful application of AI in these areas also faces challenges, such as the need for high‐quality large datasets, managing the overwhelming influx of data (data deluge), and addressing data drift, which can affect the accuracy and reliability of AI models. These challenges may slow progress and delay the clinical implementation of AI‐driven solutions in healthcare. Created with BioRender.com.

The interaction of formulation, process factors, and controlled release in DDSs is inherently complex and nonlinear, making optimization challenging for achieving successful therapeutic effects. Neural network designs are particularly well‐suited for addressing these issues. These architectures consist of multiple interconnected layers, each containing nodes that enable prediction, classification, or recognition based on input data. AI approaches have been developed to predict the performance of drug combinations by analyzing pharmacological synergies, leading to more precise and effective therapies. AI‐based optimization also has significant applications in combination drug delivery, where different types of NPs are employed to enhance drug localization at tumor sites, resulting in improved treatment outcomes^[^ ^228^, ^236^ ^]^ (**Figure** **10**).

Furthermore, by integrating diverse data sources, AI has shown great potential in understanding complex biological interactions, such as those involving cellular membranes. Inputs like drug interactions, phenotypic data, chemical interactions, and genomic information are critical for optimizing drug delivery and enhancing pharmacokinetic analyses. AI can be leveraged to make more accurate predictions and optimize nanocarrier delivery systems, leading to better‐targeted therapies and improved patient outcomes (**Table** **6**).

**Table 6:** The list of tools and platforms are widely used for the AI‐driven design of nanocarriers in drug delivery.

Aspect	Details	Example	Advantages	Limitations	Tools/Website
Material selection	AI models predict the best materials (e.g., polymers, lipids, metals) for constructing nanocarriers based on desired properties such as biocompatibility, stability, and drug release profiles.	AI‐driven material discovery tools	Accelerates discovery of new materials, optimizes biocompatibility and stability, and reduces cost	Relies on the quality of input data and algorithms, the potential for overlooking unconventional materials	Materials Studio, Citrine Informatics
Structural optimization	AI optimizes the size, shape, and surface properties of nanocarriers to enhance their delivery efficiency, targeting capabilities, and circulation time.	Machine learning for structural prediction	Enhances targeting specificity, improves circulation time, and optimizes drug release kinetics	Complex modeling required, potential for overfitting if trained on limited data	Schrödinger's Maestro, NanoHub.org
Drug loading and release profile	AI models predict how to load drugs into nanocarriers efficiently and how they will be released over time, ensuring a controlled and sustained release.	Deep learning for drug encapsulation	Optimizes drug loading capacity, ensures controlled release, and improves therapeutic efficacy	Requires accurate models and extensive training data, potential for variability in real‐world conditions	DeepChem, MATLAB
Targeting ligand design	AI designs and predicts the effectiveness of targeting ligands (e.g., antibodies, peptides) that can be attached to nanocarriers for site‐specific drug delivery.	AI‐based ligand screening tools	Enhances targeting accuracy, reduces off‐target effects, and improves therapeutic outcomes	Complexity in ligand–receptor interactions, potential challenges in scaling up for clinical use	Rosetta, ChEMBL, AutoDock
Toxicity prediction	AI models predict the potential toxicity of nanocarriers, including cytotoxicity, immunogenicity, and long‐term safety, enabling safer design choices.	Toxicity prediction models	Reduces the risk of adverse effects, accelerates safety assessment, and minimizes animal testing	Dependence on existing toxicity data, potential for false positives/negatives in predictions	ProTox‐II, Tox21, ADMET Predictor
Pharmacokinetics and pharmacodynamics (PK/PD) modeling	AI simulates the PK/PD profiles of nanocarriers to predict how they will distribute, metabolize, and excrete in the body, optimizing their therapeutic efficacy.	AI‐enhanced PK/PD simulations	Predicts in vivo behavior more accurately, optimizes dosing regimens, and reduces trial and error	Requires detailed biological data, potential limitations in translating models to human biology	Simcyp Simulator, PK‐Sim, GastroPlus
Manufacturing process optimization	AI optimizes the manufacturing process of nanocarriers, ensuring scalability, consistency, and cost‐effectiveness.	AI‐driven process optimization tools	Enhances reproducibility, reduces production costs, and shortens time to market	Challenges in adapting to different manufacturing scales, potential for integration issues	BIOVIA Process, Mathematica
Personalized medicine	AI can be used to design nanocarriers tailored to individual patient profiles, considering factors like genetic makeup, disease state, and metabolism.	AI‐powered personalized nanomedicine	Tailors treatment to individual patients improves efficacy and reduces adverse effects	Requires extensive patient data, ethical and privacy concerns, high complexity in implementation	GNS Healthcare, IBM Watson for Health
Simulation of nanocarrier behavior	AI models simulate the interaction of nanocarriers with biological systems, predicting outcomes like cellular uptake, immune response, and therapeutic efficacy.	Molecular dynamics and AI‐based simulations	Provides insights into nanocarrier behavior in complex environments, reduces the need for in vivo testing	Computationally intensive, may require significant computational resources for accurate simulations	GROMACS, AMBER, NanoSim
Virtual screening for nanocarrier design	AI‐driven virtual screening can identify promising nanocarrier designs by rapidly evaluating thousands of potential candidates.	Virtual screening platforms	Speeds up the design process, identifies novel nanocarrier architectures, and reduces experimental costs	May overlook unconventional designs, depending on the quality of the screening algorithm	AutoDock Vina, Schrödinger's Glid

## 8 Conclusion and Future Perspectives

In conclusion, nanocarrier drugs have revolutionized healthcare and pharmaceutical systems, offering novel solutions to the longstanding challenges in drug delivery. The integration of advanced nanotechnologies into drug delivery highlights their profound impact on modern medicine. This review explores a multitude of nanocarrier designs specifically tailored for therapeutic delivery, intending to overcome the diverse biological barriers encountered in different patient populations and diseases. Targeted delivery, characterized by the precise placement of medicines in the tissue of interest, is a pivotal drug delivery aspect. Mechanisms focused on medication targeting should ideally be capable of regulating the fate of the drugs upon their entry into the body. Nanocarriers, with their unique properties, have shown remarkable potential in facilitating enhanced delivery of poorly soluble drugs, thereby reshaping the therapeutic landscape for various hard‐to‐treat diseases.

The ability of nanocarriers to improve drug delivery efficiency has already begun to have a profound impact on the treatment of various ailments. By overcoming barriers, such as limited solubility, rapid clearance, and off‐target effects, nanocarrier‐based therapies hold promise for significantly improving the quality of life of a broad spectrum of patients. Some studies have discussed the multiple factors involved in the generation of nano–bio interfaces. These factors, such as slight differences in size, can modify the surface structure and functionality of the nanomaterials, leading to the reconformation of biomolecules. However, the development of a nano–bio interface can also be advantageous, as it can reduce the biotoxicity of nanomaterials and increase their suitability for biological applications.^[^ ^237^ ^]^ We anticipate that additional advances in DDSs and nanotechnology will further enhance and improve therapeutic interventions.^[^ ^208^ ^]^ These advancements have the potential to not only enhance the efficacy and safety of treatments but also enable more precise targeting of specific tissues or cells, minimize side effects, and optimize therapeutic outcomes.

The widespread adoption of nanocarrier drugs has revolutionized clinical practice and patient care. Maintaining ethical standards in nanocarrier research, especially when involving human or animal studies, is essential for ensuring the safety, efficacy, and social acceptability of nanotechnology in medicine. Risk–benefit analysis is a critical component of ethical considerations, providing a structured evaluation of the potential benefits of the research against the risks to participants or test subjects. Regulatory compliance is another vital aspect, with ethical reports often including detailed assessments of how the research aligns with regulatory guidelines. Ethical boards, such as Institutional Review Boards (IRBs) for human studies or Institutional Animal Care and Use Committees (IACUCs) for animal studies, play a crucial role by providing an independent evaluation of the research's ethical soundness.

As advancements in nanotechnology continue, the convergence of AI with nanotechnology holds promise for further refinement of targeted delivery mechanisms. The synergy between AI and nanotechnology has the potential to catalyze the development of highly personalized and precise DDSs. In this envisioned healthcare landscape, tailored treatments will become the norm, with therapies precisely optimized to target specific tissues or cells, while minimizing off‐target effects. By harnessing the power of AI‐driven algorithms to analyze vast amounts of patient data, healthcare providers can tailor drug delivery strategies for individual patient profiles, thereby ensuring the highest level of therapeutic efficacy and safety.

Ultimately, the ongoing development and refinement of nanocarrier‐based therapies represents a paradigm shift in medicine, offering unprecedented opportunities to improve patient health and well‐being on a global scale. Given the transformative potential of nanotechnology and AI, we can use them in a new precision medicine era, where each patient receives personalized treatments tailored to their unique physiological characteristics and medical needs. By leveraging the capabilities of nanocarriers and scientific progress, we can look forward to a future in which personalized medicine becomes increasingly tailored and effective, ultimately leading to improved patient care and outcomes.

## Conflict of Interest

The authors declare no conflict of interest.

## Author Contributions

**Minhye Kim** wrote the partial script. **Myeongyeon Shin and Yaping Zhao** took care of analysis and Investigation. **Mrinmoy Ghosh** took care of writing, original draft, review, and editing the manuscript. **Young‐Ok Son** took care of supervision, dunding acquisition, and project administration. All authors were involved in writing the manuscript and provided their final approval for the submitted and published versions.

## References

[1] T. Schlick , S. Portillo‐Ledesma , Nat. Comput. Sci. 2021, 1, 321. 34423314 10.1038/s43588-021-00060-9 PMC8378674

[2] R. Yue , A. Dutta , npj Syst. Biol. Appl. 2022, 8, 37. 36192551 10.1038/s41540-022-00247-4 PMC9528884

[3] J. Majumder , O. Taratula , T. Minko , Adv. Drug Delivery Rev. 2019, 144, 57. 10.1016/j.addr.2019.07.010 PMC6748653 31400350

[4] U. Ruman , S. Fakurazi , M. J. Masarudin , M. Z. Hussein , Int. J. Nanomed. 2020, 15, 1437. 10.2147/IJN.S236927 PMC7060777 32184597

[5] S.‐J. Kim , N. Puranik , D. Yadav , J.‐O. Jin , P. C. W. Lee , Int. J. Nanomed. 2023, 18, 2659. 10.2147/IJN.S406415 PMC10202211 37223276

[6] S. R. Prasad , T. S. S. Kumar , A. Jayakrishnan , Biomed. Mater. 2021, 16, 044107. 10.1088/1748-605X/abf7d5 33853043

[7] F. Din , W. Aman , I. Ullah , O. S. Qureshi , O. Mustapha , S. Shafique , A. Zeb , Int. J. Nanomed. 2017, 12, 7291. 10.2147/IJN.S146315 PMC5634382 29042776

[8] K. Ulbrich , K. Holá , V. Šubr , A. Bakandritsos , J. Tuček , R. Zbořil , Chem. Rev. 2016, 116, 5338. 27109701 10.1021/acs.chemrev.5b00589

[9] T. C. Ezike , U. S. Okpala , U. L. Onoja , C. P. Nwike , E. C. Ezeako , O. J. Okpara , C. C. Okoroafor , S. C. Eze , O. L. Kalu , E. C. Odoh , U. G. Nwadike , J. O. Ogbodo , B. U. Umeh , E. C. Ossai , B. C. Nwanguma , Heliyon 2023, 9, e17488. 37416680 10.1016/j.heliyon.2023.e17488 PMC10320272

[10] N. Wang , L. Chen , W. Huang , Z. Gao , M. Jin , Nanomaterials 2024, 14, 557. 38607092 10.3390/nano14070557 PMC11013305

[11] R. Van Der Meel , E. Sulheim , Y. Shi , F. Kiessling , W. J. M. Mulder , T. Lammers , Nat. Nanotechnol. 2019, 14, 1007. 31695150 10.1038/s41565-019-0567-y PMC7227032

[12] B. Clares , M. A. Ruiz , V. Gallardo , J. L. Arias , Curr. Med. Chem. 2012, 19, 3203. 22612704 10.2174/092986712800784676

[13] I. S. Alferiev , K. Zhang , Z. Folchman‐Wagner , R. F. Adamo , D. T. Guerrero , I. Fishbein , D. Soberman , R. J. Levy , M. Chorny , Pharmaceutics 2024, 16, 188. 38399249 10.3390/pharmaceutics16020188 PMC10892638

[14] A. Shah , S. Aftab , J. Nisar , M. N. Ashiq , F. J. Iftikhar , J. Drug Delivery Sci. Technol. 2021, 62, 102426.

[15] A. A. Yetisgin , S. Cetinel , M. Zuvin , A. Kosar , O. Kutlu , Molecules 2020, 25, 2193. 32397080 10.3390/molecules25092193 PMC7248934

[16] F. Ulucan‐Karnak , G. Camci‐Unal , B. Karacaoglu , M. Ö. Seydibeyoğlu , in A Handbook of Artificial Intelligence in Drug Delivery (Ed: A. Philip , A. Shahiwala , M. Rashid , M. Faiyazuddin ), Academic Press, Cambridge, MA 2023, pp. 371–394.

[17] J. K. Patra , G. Das , L. F. Fraceto , E. V. R. Campos , M. P. Rodriguez‐Torres , L. S. Acosta‐Torres , L. A. Diaz‐Torres , R. Grillo , M. K. Swamy , S. Sharma , S. Habtemariam , H.‐S. Shin , J. Nanobiotechnol. 2018, 16, 71. 10.1186/s12951-018-0392-8 PMC6145203 30231877

[18] S. Ashique , N. K. Sandhu , V. Chawla , P. A. Chawla , Curr. Drug Delivery 2021, 18, 1435. 10.2174/1567201818666210609161301 34151759

[19] A. Hafner , J. Lovrić , G. P. Lakoš , I. Pepić , Int. J. Nanomed. 2014, 9, 1005. 10.2147/IJN.S55359 PMC3933707 24600222

[20] D. J. Irvine , E. L. Dane , Nat. Rev. Immunol. 2020, 20, 321. 32005979 10.1038/s41577-019-0269-6 PMC7536618

[21] S. Priya , U. Batra , S. R.N., S. Sharma , A. Chaurasiya , G. Singhvi , Int. J. Biol. Macromol. 2022, 218, 209. 35872310 10.1016/j.ijbiomac.2022.07.118

[22] S. Priya , V. M. Desai , G. Singhvi , ACS Omega 2023, 8, 74. 36643539 10.1021/acsomega.2c05976 PMC9835629

[23] A. Chowdhury , S. Kunjiappan , T. Panneerselvam , B. Somasundaram , C. Bhattacharjee , Int. Nano Lett. 2017, 7, 91. 10.1007/s11626-017-0136-3 28342023

[24] D. Taylor , in Pharmaceuticals in the Environment (Eds: R. E. Hester , R. M. Harrison ), The Royal Society of Chemistry, Piccadilly, London 2015.

[25] C. H. Sabine Gressler , A. Pavlicek , C. Zafiu , E.‐K. Ehmoser , F. Part , B. Giese , Nanocarrier. Part I: Overview and Categorization of Nanocarriers, German Environment Agency, Vienna 2024.

[26] F. Farjadian , A. Ghasemi , O. Gohari , A. Roointan , M. Karimi , M. R. Hamblin , Nanomedicine 2019, 14, 93. 30451076 10.2217/nnm-2018-0120 PMC6391637

[27] S. Barthold , N. Kunschke , X. Murgia , B. Loretz , C. S. Carvalho‐Wodarz , C.‐M. Lehr , J. Aerosol Med. Pulm. Drug Delivery 2023, 36, 144. 10.1089/jamp.2023.29089.sb 37310368

[28] S. Bisso , J.‐C. Leroux , Int. J. Pharm. 2020, 578, 119098. 32018018 10.1016/j.ijpharm.2020.119098

[29] B. Wang , K. Kostarelos , B. J. Nelson , L. Zhang , Adv. Mater. 2021, 33, 2002047. 10.1002/adma.202002047 33617105

[30] M. Ruiz , M. Gantner , A. Talevi , Recent Pat. Anti‐Cancer Drug Discovery 2013, 9, 99. 10.2174/1574891x113089990038 23578193

[31] V. R. L. Constantino , M. P. Figueiredo , V. R. Magri , D. Eulálio , V. R. R. Cunha , A. C. S. Alcântara , G. F. Perotti , Pharmaceutics 2023, 15, 413. 36839735 10.3390/pharmaceutics15020413 PMC9961265

[32] S. Aggarwal , A. Chakravarty , S. Ikram , Int. J. Biol. Macromol. 2021, 167, 962. 33186644 10.1016/j.ijbiomac.2020.11.052

[33] S. Jacob , F. Kather , M. Morsy , S. Boddu , M. Attimarad , J. Shah , P. Shinu , A. Nair , Nanomaterials 2024, 14, 672. 38668166 10.3390/nano14080672 PMC11054677

[34] H. Ragelle , F. Danhier , V. Préat , R. Langer , D. G. Anderson , Expert Opin. Drug Delivery 2017, 14, 851. 10.1080/17425247.2016.1244187 27730820

[35] A. V. Samrot , T. C. Sean , T. Kudaiyappan , U. Bisyarah , A. Mirarmandi , E. Faradjeva , A. Abubakar , H. H. Ali , J. L. A. Angalene , S. Suresh Kumar , Int. J. Biol. Macromol. 2020, 165, 3088. 33098896 10.1016/j.ijbiomac.2020.10.104

[36] N. Gupta , C. Sarkar , S. Saha , in Biodegradable Polymers and their Emerging Applications (Eds: S. Saha , C. Sarkar ), Springer Nature Singapore, Singapore 2023, pp. 149–168.

[37] A. Zielińska , F. Carreiró , A. M. Oliveira , A. Neves , B. Pires , D. N. Venkatesh , A. Durazzo , M. Lucarini , P. Eder , A. M. Silva , A. Santini , E. B. Souto , Molecules 2020, 25, 3731. 32824172 10.3390/molecules25163731 PMC7464532

[38] M. Haider , K. Z. Zaki , M. R. El Hamshary , Z. Hussain , G. Orive , H. O. Ibrahim , J. Adv. Res. 2022, 39, 237. 35777911 10.1016/j.jare.2021.11.008 PMC9263757

[39] S. Li , R. Xing , R. Chang , Q. Zou , X. Yan , Curr. Opin. Colloid Interface Sci. 2018, 35, 17.

[40] F.‐D. Zhu , Y.‐J. Hu , L. Yu , X.‐G. Zhou , J.‐M. Wu , Y. Tang , D.‐L. Qin , Q.‐Z. Fan , A.‐G. Wu , Front. Pharmacol. 2021, 12, 683935. 34122112 10.3389/fphar.2021.683935 PMC8187807

[41] F. Lai , E. Pini , G. Angioni , M. L. Manca , J. Perricci , C. Sinico , A. M. Fadda , Eur. J. Pharm. Biopharm. 2011, 79, 552. 21820052 10.1016/j.ejpb.2011.07.005

[42] E. G. Fuller , G. M. Scheutz , A. Jimenez , P. Lewis , S. Savliwala , S. Liu , B. S. Sumerlin , C. Rinaldi , Int. J. Pharm. 2019, 572, 118796. 31678389 10.1016/j.ijpharm.2019.118796

[43] S. Su , P. M. Kang , Pharmaceutics 2020, 12, 837. 32882875 10.3390/pharmaceutics12090837 PMC7559885

[44] B. Eftekharzadeh , J. G. Daigle , L. E. Kapinos , A. Coyne , J. Schiantarelli , Y. Carlomagno , C. Cook , S. J. Miller , S. Dujardin , A. S. Amaral , J. C. Grima , R. E. Bennett , K. Tepper , M. Deture , C. R. Vanderburg , B. T. Corjuc , S. L. Devos , J. A. Gonzalez , J. Chew , S. Vidensky , F. H. Gage , J. Mertens , J. Troncoso , E. Mandelkow , X. Salvatella , R. Y. H. Lim , L. Petrucelli , S. Wegmann , J. D. Rothstein , B. T. Hyman , Neuron 2018, 99, 925. 30189209 10.1016/j.neuron.2018.07.039 PMC6240334

[45] A. Nakamura , N. Kaneko , V. L. Villemagne , T. Kato , J. Doecke , V. Doré , C. Fowler , Q.‐X. Li , R. Martins , C. Rowe , T. Tomita , K. Matsuzaki , K. Ishii , K. Ishii , Y. Arahata , S. Iwamoto , K. Ito , K. Tanaka , C. L. Masters , K. Yanagisawa , Nature 2018, 554, 249. 29420472 10.1038/nature25456

[46] V. Pavot , M. Berthet , J. Rességuier , S. Legaz , N. Handké , S. C. Gilbert , S. Paul , B. Verrier , Nanomedicine 2014, 9, 2703. 25529572 10.2217/nnm.14.156

[47] S. Fan , Y. Zheng , X. Liu , W. Fang , X. Chen , W. Liao , X. Jing , M. Lei , E. Tao , Q. Ma , X. Zhang , R. Guo , J. Liu , Drug Delivery 2018, 25, 1091. 30107760 10.1080/10717544.2018.1461955 PMC6116673

[48] N. Nehal , B. Nabi , S. Rehman , A. Pathak , A. Iqubal , S. A. Khan , M. S. Yar , S. Parvez , S. Baboota , J. Ali , Int. J. Biol. Macromol. 2021, 167, 605. 33278450 10.1016/j.ijbiomac.2020.11.207

[49] M. Agrawal , M. Ajazuddin , D. K. Tripathi , S. Saraf , S. Saraf , S. G. Antimisiaris , S. Mourtas , M. Hammarlund‐Udenaes , A. Alexander , J. Controlled Release 2017, 260, 61. 10.1016/j.jconrel.2017.05.019 28549949

[50] T. Li , D. Cipolla , T. Rades , B. J. Boyd , J. Controlled Release 2018, 288, 96. 10.1016/j.jconrel.2018.09.001 30184465

[51] M. T. Manzari , Y. Shamay , H. Kiguchi , N. Rosen , M. Scaltriti , D. A. Heller , Nat. Rev. Mater. 2021, 6, 351. 34950512 10.1038/s41578-020-00269-6 PMC8691416

[52] S. S. Shazwani , A. Marlina , M. Misran , ACS Omega 2024, 9, 17379. 38645372 10.1021/acsomega.4c00091 PMC11024946

[53] T. Nii , F. Ishii , Int. J. Pharm. 2005, 298, 198. 15951143 10.1016/j.ijpharm.2005.04.029

[54] S. B. Kulkarni , G. V. Betageri , M. Singh , J. Microencapsulation 1995, 12, 229. 7650588 10.3109/02652049509010292

[55] Ç. Melis , S. Ali Demir , B. Seyda , in Application of Nanotechnology in Drug Delivery (Ed: S. Ali Demir , IntechOpen, Rijeka 2014, Ch. 1.

[56] M. P. Nikolova , E. M. Kumar , M. S. Chavali , Pharmaceutics 2022, 14, 2195. 36297630 10.3390/pharmaceutics14102195 PMC9608678

[57] Y. Kang , H. Jung , J. Oh , D. Song , CNS Neurosci. Ther. 2016, 22, 817. 27350533 10.1111/cns.12580 PMC6492798

[58] A. A. Yaroslavov , A. V. Sybachin , O. V. Zaborova , A. B. Zezin , Y. Talmon , M. Ballauff , F. M. Menger , Adv. Colloid Interface Sci. 2015, 226, 54. 26372095 10.1016/j.cis.2015.08.011

[59] M. Jumaa , B. W. Müller , Eur. J. Pharm. Sci. 2000, 9, 285. 10594386 10.1016/s0928-0987(99)00071-8

[60] U. Scheffel , B. A. Rhodes , T. K. Natarajan , H. N. Wagner Jr , J. Nucl. Med. 1972, 13, 498. 5033902

[61] F. A. Cupaioli , F. A. Zucca , D. Boraschi , L. Zecca , Prog. Neurobiol. 2014, 119–120, 20. 10.1016/j.pneurobio.2014.05.002 24820405

[62] S. Mukherjee , S. Ray , R. S. Thakur , Indian J. Pharm. Sci. 2009, 71, 349. 20502539 10.4103/0250-474X.57282 PMC2865805

[63] S. Banerjee , J. Pillai , Expert Opin. Drug Metab. Toxicol. 2019, 15, 499. 31104522 10.1080/17425255.2019.1621289

[64] A. Raza , F. B. Sime , P. J. Cabot , F. Maqbool , J. A. Roberts , J. R. Falconer , Drug Discovery Today 2019, 24, 858. 30654055 10.1016/j.drudis.2019.01.004

[65] A. K. Sachdeva , S. Misra , I. Pal Kaur , K. Chopra , Eur. J. Pharmacol. 2015, 747, 132. 25449035 10.1016/j.ejphar.2014.11.014

[66] P. Mittal , A. Saharan , R. Verma , F. M. A. Altalbawy , M. A. Alfaidi , G. E.‐S. Batiha , W. Akter , R. K. Gautam , M. S. Uddin , M. S. Rahman , M. H. Baig , BioMed Res. Int. 2021, 2021, 11. 10.1155/2021/8844030 PMC7902124 33644232

[67] A. S. Chauhan , Molecules 2018, 23, 938.29670005

[68] M. Kaurav , S. Ruhi , H. A. Al‐Goshae , A. K. Jeppu , D. Ramachandran , R. K. Sahu , A. K. Sarkar , J. Khan , A. M. Ashif Ikbal , Front. Pharmacol. 2023, 14, 1159131. 37006997 10.3389/fphar.2023.1159131 PMC10060650

[69] E. Gillies , J. Frechet , Drug Discovery Today 2005, 10, 35. 15676297 10.1016/S1359-6446(04)03276-3

[70] A. K. Patri , I. J. Majoros , J. R. Baker , Curr. Opin. Chem. Biol. 2002, 6, 466. 12133722 10.1016/s1367-5931(02)00347-2

[71] W. Liyanage , T. Wu , S. Kannan , R. M. Kannan , ACS Appl. Mater. Interfaces 2022, 14, 46290. 36214413 10.1021/acsami.2c13129

[72] S. Mignani , X. Shi , J. Rodrigues , H. Tomás , J.‐P. Majoral , Drug Discovery Today 2022, 27, 1251. 34999213 10.1016/j.drudis.2022.01.001

[73] A. S. Khatik , S. Kurdhane , S. Batheji , U. Gupta , in Molecular Pharmaceutics and Nano Drug Delivery (Eds: U. Gupta , A. K. Goyal ), Academic Press, Cambridge, MA 2024, pp. 237–267.

[74] J. Wang , B. Li , L. Qiu , X. Qiao , H. Yang , J. Biol. Eng. 2022, 16, 18. 35879774 10.1186/s13036-022-00298-5 PMC9317453

[75] R. M. Kannan , I. Pitha , K. S. Parikh , Adv. Drug Delivery Rev. 2023, 200, 115005. 10.1016/j.addr.2023.115005 37419213

[76] N. Rades , K. Licha , R. Haag , Polymers 2018, 10, 595. 30966629 10.3390/polym10060595 PMC6403730

[77] J. Dernedde , A. Rausch , M. Weinhart , S. Enders , R. Tauber , K. Licha , M. Schirner , U. Zügel , A. Von Bonin , R. Haag , Proc. Natl. Acad. Sci. U.S.A. 2010, 107, 19679. 21041668 10.1073/pnas.1003103107 PMC2993387

[78] F. Reisbeck , S. Wedepohl , M. Dimde , A.‐C. Schmitt , J. Dernedde , M. Álvaro‐Benito , C. Freund , R. Haag , J. Mater. Chem. B 2021, 10, 96. 34881771 10.1039/d1tb02144c

[79] D. Maysinger , J. Ji , A. Moquin , S. Hossain , M. A. Hancock , I. Zhang , P. K. Y. Chang , M. Rigby , M. Anthonisen , P. Grütter , J. Breitner , R. A. Mckinney , S. Reimann , R. Haag , G. Multhaup , ACS Chem. Neurosci. 2018, 9, 260. 29078046 10.1021/acschemneuro.7b00301

[80] A. Elzayat , I. Adam‐Cervera , O. Álvarez‐Bermúdez , R. Muñoz‐Espí , Colloids Surf., B 2021, 203, 111764. 10.1016/j.colsurfb.2021.111764 33892282

[81] S. Yadav , S. K. Gandham , R. Panicucci , M. M. Amiji , Nanomedicine 2016, 12, 987. 26767514 10.1016/j.nano.2015.12.374 PMC4837036

[82] V. P. Torchilin , Pharm. Res. 2006, 24, 1. 17109211 10.1007/s11095-006-9132-0

[83] S. Perumal , R. Atchudan , W. Lee , Polymers 2022, 14, 2510. 35746086 10.3390/polym14122510 PMC9230755

[84] Y. Zhang , Y. Huang , S. Li , AAPS PharmSciTech 2014, 15, 862. 24700296 10.1208/s12249-014-0113-z PMC4113619

[85] M. Yokoyama , J. Controlled Release 1998, 50, 79. 10.1016/s0168-3659(97)00115-6 9685875

[86] M. Rizwanullah , M. Alam , M. Harshita , S. R. Mir , M. M. A. Rizvi , S. Amin , Curr. Pharm. Des. 2020, 26, 1206. 31951163 10.2174/1381612826666200116150426

[87] L. Zhang , J. M. Chan , F. X. Gu , J.‐W. Rhee , A. Z. Wang , A. F. Radovic‐Moreno , F. Alexis , R. Langer , O. C. Farokhzad , ACS Nano 2008, 2, 1696. 19206374 10.1021/nn800275r PMC4477795

[88] J.‐F. Le Meins , C. Schatz , S. Lecommandoux , O. Sandre , Mater. Today 2013, 16, 397.

[89] A. Olubummo , M. Schulz , B.‐D. Lechner , P. Scholtysek , K. Bacia , A. Blume , J. Kressler , W. H. Binder , ACS Nano 2012, 6, 8713. 22950802 10.1021/nn3023602

[90] M. Chemin , P.‐M. Brun , S. Lecommandoux , O. Sandre , J.‐F. Le Meins , Soft Matter 2012, 8, 2867.

[91] P. I. Kuzmin , S. A. Akimov , Y. A. Chizmadzhev , J. Zimmerberg , F. S. Cohen , Biophys. J. 2005, 88, 1120. 15542550 10.1529/biophysj.104.048223 PMC1305117

[92] R. M. Perera , S. Gupta , T. Li , C. J. Van Leeuwen , M. Bleuel , K. Hong , G. J. Schneider , ACS Appl. Polym. Mater. 2022, 4, 8858.

[93] M. Fauquignon , E. Ibarboure , J.‐F. Le Meins , Biophys. J. 2022, 121, 61. 34890579 10.1016/j.bpj.2021.12.005 PMC8758416

[94] C. Salvador‐Morales , L. Zhang , R. Langer , O. C. Farokhzad , Biomaterials 2009, 30, 2231. 19167749 10.1016/j.biomaterials.2009.01.005 PMC2699891

[95] D. Sivadasan , M. H. Sultan , O. Madkhali , Y. Almoshari , N. Thangavel , Pharmaceutics 2021, 13, 1291. 34452251 10.3390/pharmaceutics13081291 PMC8399620

[96] J. M. Chan , L. Zhang , K. P. Yuet , G. Liao , J.‐W. Rhee , R. Langer , O. C. Farokhzad , Biomaterials 2009, 30, 1627. 19111339 10.1016/j.biomaterials.2008.12.013

[97] S.‐M. Lee , H. Chen , T. V. O’Halloran , S. T. Nguyen , J. Am. Chem. Soc. 2009, 131, 9311. 19527027 10.1021/ja9017336 PMC3650134

[98] O. V. Gerasimov , J. A. Boomer , M. M. Qualls , D. H. Thompson , Adv. Drug Delivery Rev. 1999, 38, 317. 10.1016/s0169-409x(99)00035-6 10837763

[99] V. A. Slepushkin , S. Simões , P. Dazin , M. S. Newman , L. S. Guo , M. C. P. De Lima , N. Düzgüneş , J. Biol. Chem. 1997, 272, 2382. 8999949 10.1074/jbc.272.4.2382

[100] S. Lee , Y. Song , B. J. Hong , K. W. Macrenaris , D. J. Mastarone , T. V. O'halloran , T. J. Meade , S. T. Nguyen , Angew. Chem. Int. Ed. Engl. 2010, 49, 9960. 21082634 10.1002/anie.201004867 PMC3096927

[101] V. Dave , K. Tak , A. Sohgaura , A. Gupta , V. Sadhu , K. R. Reddy , J. Microbiol. Methods 2019, 160, 130. 30898602 10.1016/j.mimet.2019.03.017

[102] J. Gao , C. Lü , X. Lü , Y. Du , J. Mater. Chem. 2007, 17, 4591.

[103] C. I. Lin , A. K. Joseph , C. K. Chang , Y. D. Lee , Biosens. Bioelectron. 2004, 20, 127. 15142585 10.1016/j.bios.2003.10.017

[104] C.‐M. J. Hu , L. Zhang , S. Aryal , C. Cheung , R. H. Fang , L. Zhang , Proc. Natl. Acad. Sci. U.S.A. 2011, 108, 10980. 21690347 10.1073/pnas.1106634108 PMC3131364

[105] J. Hu , G. Zhang , S. Liu , Chem. Soc. Rev. 2012, 41, 5933. 22695880 10.1039/c2cs35103j

[106] E. Lallana , A. Sousa‐Herves , F. Fernandez‐Trillo , R. Riguera , E. Fernandez‐Megia , Pharm. Res. 2012, 29, 1. 21913032 10.1007/s11095-011-0568-5

[107] J. Shi , Z. Xiao , A. R. Votruba , C. Vilos , O. C. Farokhzad , Angew. Chem. Int. Ed. Engl. 2011, 50, 7027. 21698724 10.1002/anie.201101554 PMC3515655

[108] D. Wu , Q. Chen , X. Chen , F. Han , Z. Chen , Y. Wang , Signal Transduction Targeted Ther. 2023, 8, 217. 10.1038/s41392-023-01481-w PMC10212980 37231000

[109] B. Jafari , M. M. Pourseif , J. Barar , M. A. Rafi , Y. Omidi , Expert. Opin. Drug Delivery 2019, 16, 583. 10.1080/17425247.2019.1614911 31107110

[110] L. Duan , X. Li , R. Ji , Z. Hao , M. Kong , X. Wen , F. Guan , S. Ma , Polymers 2023, 15, 2196. 37177342 10.3390/polym15092196 PMC10181407

[111] S. R. Hwang , K. Kim , Arch. Pharm. Res. 2014, 37, 24. 24170511 10.1007/s12272-013-0272-6

[112] M. J. Mitchell , M. M. Billingsley , R. M. Haley , M. E. Wechsler , N. A. Peppas , R. Langer , Nat. Rev. Drug Discovery 2021, 20, 101. 33277608 10.1038/s41573-020-0090-8 PMC7717100

[113] R. G. R. Pinheiro , A. J. Coutinho , M. Pinheiro , A. R. Neves , Int. J. Mol. Sci. 2021, 22, 11654.34769082

[114] M. Demeule , J. Currie , Y. Bertrand , C. Ché , T. Nguyen , A. Régina , R. Gabathuler , J. Castaigne , R. Béliveau , J. Neurochem. 2008, 106, 1534. 18489712 10.1111/j.1471-4159.2008.05492.x

[115] A. Nykjaer , T. E. Willnow , Trends Cell Biol. 2002, 12, 273. 12074887 10.1016/s0962-8924(02)02282-1

[116] I. Singh , R. Swami , M. K. Jeengar , W. Khan , R. Sistla , Chem. Phys. Lipids 2015, 188, 1. 25819559 10.1016/j.chemphyslip.2015.03.003

[117] H. J. Byeon , L. Q. Thao , S. Lee , S. Y. Min , E. S. Lee , B. S. Shin , H.‐G. Choi , Y. S. Youn , J. Controlled Release 2016, 225, 301. 10.1016/j.jconrel.2016.01.046 26826308

[118] A. R. Neves , J. F. Queiroz , S. Reis , J. Nanobiotechnol. 2016, 14, 27. 10.1186/s12951-016-0177-x PMC4826547 27061902

[119] A. Zensi , D. Begley , C. Pontikis , C. Legros , L. Mihoreanu , S. Wagner , C. Büchel , H. Von Briesen , J. Kreuter , J. Controlled Release 2009, 137, 78. 10.1016/j.jconrel.2009.03.002 19285109

[120] T. Moos , E. H. Morgan , Cell. Mol. Neurobiol. 2000, 20, 77. 10690503 10.1023/A:1006948027674 PMC11537550

[121] J. A. Loureiro , B. Gomes , G. Fricker , M. A. N. Coelho , S. Rocha , M. C. Pereira , Colloids Surf., B 2016, 145, 8. 10.1016/j.colsurfb.2016.04.041 27131092

[122] Y.‐C. Kuo , L.‐J. Wang , J. Taiwan Inst. Chem. Eng. 2014, 45, 755.

[123] B. Ji , J. Maeda , M. Higuchi , K. Inoue , H. Akita , H. Harashima , T. Suhara , Life Sci. 2006, 78, 851. 16165165 10.1016/j.lfs.2005.05.085

[124] K. Hu , Y. Shi , W. Jiang , J. Han , S. Huang , X. Jiang , Int. J. Pharm. 2011, 415, 273. 21651967 10.1016/j.ijpharm.2011.05.062

[125] K. Hu , J. Li , Y. Shen , W. Lu , X. Gao , Q. Zhang , X. Jiang , J. Controlled Release 2009, 134, 55. 10.1016/j.jconrel.2008.10.016 19038299

[126] M. Schneider , F. Stracke , S. Hansen , U. F. Schaefer , Dermatoendocrinol 2009, 1, 197. 20592791 10.4161/derm.1.4.9501 PMC2835875

[127] L. Liu , W. Zhao , Q. Ma , Y. Gao , W. Wang , X. Zhang , Y. Dong , T. Zhang , Y. Liang , S. Han , J. Cao , X. Wang , W. Sun , H. Ma , Y. Sun , Nanoscale Adv. 2023, 5, 1527. 36926556 10.1039/d2na00530a PMC10012846

[128] T. L. De Brum , L. A. Fiel , R. V. Contri , S. S. Guterres , A. R. Pohlmann , J. Nanosci. Nanotechnol. 2015, 15, 773. 26328441 10.1166/jnn.2015.9185

[129] R. Alvarez‐Román , A. Naik , Y. N. Kalia , R. H. Guy , H. Fessi , J. Controlled Release 2004, 99, 53. 10.1016/j.jconrel.2004.06.015 15342180

[130] W. Zhang , J. Gao , Q. Zhu , M. Zhang , X. Ding , X. Wang , X. Hou , W. Fan , B. Ding , X. Wu , Int. J. Pharm. 2010, 402, 205. 20932886 10.1016/j.ijpharm.2010.09.037

[131] M. Sguizzato , E. Esposito , R. Cortesi , Int. J. Mol. Sci. 2021, 22, 8319. 34361084 10.3390/ijms22158319 PMC8348303

[132] E. B. Souto , I. Baldim , W. P. Oliveira , R. Rao , N. Yadav , F. M. Gama , S. Mahant , Expert Opin. Drug Delivery 2020, 17, 357. 10.1080/17425247.2020.1727883 32064958

[133] M. Uner , Pharmazie 2006, 61, 375. 16724531

[134] S. K. Zingales , J. H. Ferguson , in A Handbook of Artificial Intelligence in Drug Delivery (Ed: A. Philip , A. Shahiwala , M. Rashid , M. Faiyazuddin ), Academic Press 2023, pp. 213–239.

[135] N. M. Sakhrani , H. Padh , Drug Des. Dev. Ther. 2013, 2013, 585. 10.2147/DDDT.S45614 PMC3718765 23898223

[136] A. R. Maity , D. Stepensky , Int. J. Pharm. 2015, 496, 268. 26516100 10.1016/j.ijpharm.2015.10.053

[137] X. Ma , N. Gong , L. Zhong , J. Sun , X.‐J. Liang , Biomaterials 2016, 97, 10. 27155363 10.1016/j.biomaterials.2016.04.026

[138] E. Blanco , H. Shen , M. Ferrari , Nat. Biotechnol. 2015, 33, 941. 26348965 10.1038/nbt.3330 PMC4978509

[139] C. Azevedo , M. H. Macedo , B. Sarmento , Drug Discovery Today 2018, 23, 944. 28919437 10.1016/j.drudis.2017.08.011 PMC7108348

[140] M. A. Lopes , B. A. Abrahim , L. M. Cabral , C. R. Rodrigues , R. M. F. Seiça , F. J. De Baptista Veiga , A. J. Ribeiro , Nanomed. Nanotechnol. Biol. Med. 2014, 10, 1139. 10.1016/j.nano.2014.02.014 24632248

[141] V. K. Pawar , J. G. Meher , Y. Singh , M. Chaurasia , B. Surendar Reddy , M. K. Chourasia , J. Controlled Release 2014, 196, 168. 10.1016/j.jconrel.2014.09.031 25305562

[142] C.‐M. Lehr , J. Controlled Release 2000, 65, 19. 10.1016/s0168-3659(99)00228-x 10699266

[143] X. Qin , C. Yu , J. Wei , L. Li , C. Zhang , Q. Wu , J. Liu , S. Q. Yao , W. Huang , Adv. Mater. 2019, 31, 1902791. 10.1002/adma.201902791 31496027

[144] G. Wei , Y. Wang , G. Yang , Y. Wang , R. Ju , Theranostics 2021, 11, 6370. 33995663 10.7150/thno.57828 PMC8120226

[145] X. He , S. Xiong , Y. Sun , M. Zhong , N. Xiao , Z. Zhou , T. Wang , Y. Tang , J. Xie , Pharmaceutics 2023, 15, 1610. 37376059 10.3390/pharmaceutics15061610 PMC10301687

[146] M. Martínez‐Negro , N. Sánchez‐Arribas , A. Guerrero‐Martínez , M. L. Moyá , C. Tros De Ilarduya , F. Mendicuti , E. Aicart , E. Junquera , Pharmaceutics 2019, 11, 632. 31783620 10.3390/pharmaceutics11120632 PMC6956073

[147] H. Derakhshankhah , S. Jafari , Biomed. Pharm. 2018, 108, 1090. 10.1016/j.biopha.2018.09.097 30372809

[148] J. Liu , H. Cabral , P. Mi , Adv. Drug Delivery Rev. 2024, 207, 115239. 10.1016/j.addr.2024.115239 38437916

[149] B. Zhao , L. Liao , Y. Zhu , Z. Hu , F. Wu , J. Lumin. 2023, 263, 120099.

[150] S. Das , S. Dey , S. Patra , A. Bera , T. Ghosh , B. Prasad , K. D. Sayala , K. Maji , A. Bedi , S. Debnath , Biomolecules 2023, 13, 1723. 38136594 10.3390/biom13121723 PMC10741882

[151] A. Hoji , T. Muhammad , M. Wubulikasimu , M. Imerhasan , H. Li , Z. Aimaiti , X. Peng , Eur. Polym. J. 2020, 141, 110058.

[152] X. Liu , S. Yu , Y. Zhang , J. Fluoresc. 2024, 10.1007/s10895-023-03562-z. 38170426

[153] W. Hu , H. Ma , B. Hou , H. Zhao , Y. Ji , R. Jiang , X. Hu , X. Lu , L. Zhang , Y. Tang , Q. Fan , W. Huang , ACS Appl. Mater. Interfaces 2016, 8, 12039. 27123534 10.1021/acsami.6b02721

[154] X.‐T. Yu , S.‐Y. Sui , Y.‐X. He , C.‐H. Yu , Q. Peng , Biomater. Adv. 2022, 135, 212725. 35929205 10.1016/j.bioadv.2022.212725

[155] Z. Shen , Q. Ma , X. Zhou , G. Zhang , G. Hao , Y. Sun , J. Cao , NPG Asia Mater. 2021, 13, 39.

[156] M. Martins , J. Vieira , C. Pereira‐Leite , N. Saraiva , A. S. Fernandes , Biology 2023, 13, 1. 38275722 10.3390/biology13010001 PMC10813373

[157] R. S. Li , C. Wen , C. Z. Huang , N. Li , TrAC Trends Anal. Chem. 2022, 156, 116714.

[158] J. Luo , P. Zhang , T. Zhao , M. Jia , P. Yin , W. Li , Z.‐R. Zhang , Y. Fu , T. Gong , ACS Nano 2019, 13, 3910. 30938986 10.1021/acsnano.8b06924

[159] G. Grancharov , V. Gancheva , M. Kyulavska , D. Momekova , G. Momekov , P. Petrov , Polymer 2016, 84, 27.

[160] A. Topete , S. Barbosa , P. Taboada , J. Appl. Polym. Sci. 2015, 132, 42650.

[161] S. Marrache , S. Dhar , Proc. Natl. Acad. Sci. 2012, 109, 16288. 22991470 10.1073/pnas.1210096109 PMC3479596

[162] C. Ganji , V. Muppala , M. Khan , G. P. Nagaraju , B. Farran , Drug Discovery Today 2023, 28, 103469. 36529353 10.1016/j.drudis.2022.103469

[163] X. Zhang , Y. Sun , R. Yang , B. Liu , Y. Liu , J. Yang , W. Liu , Biomaterials 2022, 287, 121656. 35792386 10.1016/j.biomaterials.2022.121656

[164] E. Hinde , K. Thammasiraphop , H. T. T. Duong , J. Yeow , B. Karagoz , C. Boyer , J. J. Gooding , K. Gaus , Nat. Nanotechnol. 2017, 12, 81. 27618255 10.1038/nnano.2016.160

[165] S. Khizar , N. Alrushaid , F. Alam Khan , N. Zine , N. Jaffrezic‐Renault , A. Errachid , A. Elaissari , Int. J. Pharm. 2023, 632, 122570. 36587775 10.1016/j.ijpharm.2022.122570

[166] A. M. Graham , J. S. Presnell , S. Bertrand , PLoS One 2017, 12, 0179545. 10.1371/journal.pone.0179545 PMC5470732 28614393

[167] L. Schito , S. Rey , M. Konopleva , Oncogene 2017, 36, 5331. 28534514 10.1038/onc.2017.119

[168] M. V. Gwangwa , A. M. Joubert , M. H. Visagie , Cell Mol. Biol. Lett. 2018, 23, 20. 29760743 10.1186/s11658-018-0088-y PMC5935986

[169] K. Tei , N. Kawakami‐Kimura , O. Taguchi , K. Kumamoto , S. Higashiyama , N. Taniguchi , K. Toda , R. Kawata , Y. Hisa , R. Kannagi , Cancer Res. 2002, 62, 6289. 12414659

[170] T. A. Partanen , J. Arola , A. Saaristo , L. Jussila , A. Ora , M. Miettinen , S. A. Stacker , M. G. Achen , K. Alitalo , FASEB J. 2000, 14, 2087. 11023993 10.1096/fj.99-1049com

[171] A. E. M. Dirkx , M. G. A. Egbrink , K. Castermans , D. W. J. Schaft , V. L. J. L. Thijssen , R. P. M. Dings , L. Kwee , K. H. Mayo , J. Wagstaff , J. C. A. B. Steege , A. W. Griffioen , FASEB J. 2006, 20, 621. 16581970 10.1096/fj.05-4493com

[172] D. Klein , Front. Oncol. 2018, 8, 367. 30250827 10.3389/fonc.2018.00367 PMC6139307

[173] H. Lan , W. Zhang , K. Jin , Y. Liu , Z. Wang , Drug Delivery 2020, 27, 1248. 32865029 10.1080/10717544.2020.1809559 PMC7470050

[174] Y. Choi , K. Jung , Exp. Mol. Med. 2023, 55, 2308. 37907742 10.1038/s12276-023-01114-w PMC10689787

[175] S. Schülke , Front. Immunol. 2018, 9, 455. 29616018 10.3389/fimmu.2018.00455 PMC5867300

[176] J. E. Lim , E. Chung , Y. Son , Sci. Rep. 2017, 7, 9417. 28842601 10.1038/s41598-017-09639-7 PMC5573373

[177] Y. Liu , J. Zhou , Q. Li , L. Li , Y. Jia , F. Geng , J. Zhou , T. Yin , Adv. Drug Delivery Rev. 2021, 172, 80. 10.1016/j.addr.2021.02.019 33705874

[178] S. Ding , X. Dong , X. Song , Cancer Cell Intern. 2023, 23, 91. 10.1186/s12935-023-02927-5 PMC10176761 37170255

[179] G. Lupo , N. Caporarello , M. Olivieri , M. Cristaldi , C. Motta , V. Bramanti , R. Avola , M. Salmeri , F. Nicoletti , C. D. Anfuso , Front. Pharmacol. 2016, 7, 519. 28111549 10.3389/fphar.2016.00519 PMC5216034

[180] H. Meng , M. Wang , H. Liu , X. Liu , A. Situ , B. Wu , Z. Ji , C. H. Chang , A. E. Nel , ACS Nano 2016, 10, 6416. 27199284 10.1021/acsnano.6b03110 PMC4928141

[181] D. Le Broc‐Ryckewaert , R. Carpentier , E. Lipka , S. Daher , C. Vaccher , D. Betbeder , C. Furman , Int. J. Pharm. 2013, 454, 712. 23707251 10.1016/j.ijpharm.2013.05.018

[182] J. Liu , M. Li , Z. Luo , L. Dai , X. Guo , K. Cai , Nano Today 2017, 15, 56.

[183] T. Cui , Z. Yan , H. Qin , Y. Sun , J. Ren , X. Qu , Small 2019, 15, 1903323. 10.1002/smll.201903323 31468717

[184] T. Ji , Y. Zhao , Y. Ding , J. Wang , R. Zhao , J. Lang , H. Qin , X. Liu , J. Shi , N. Tao , Z. Qin , G. Nie , Y. Zhao , Angew. Chem., Int. Ed. 2016, 55, 1050. 10.1002/anie.201506262 PMC4736689 26283097

[185] K. Jung , T. Heishi , O. F. Khan , P. S. Kowalski , J. Incio , N. N. Rahbari , E. Chung , J. W. Clark , C. G. Willett , A. D. Luster , S. H. Yun , R. Langer , D. G. Anderson , T. P. Padera , R. K. Jain , D. Fukumura , J. Clin. Invest. 2017, 127, 3039. 28691930 10.1172/JCI93182 PMC5531423

[186] Y. S. Kim , S. B. Ho , Curr. Gastroenterol. Rep. 2010, 12, 319. 20703838 10.1007/s11894-010-0131-2 PMC2933006

[187] S. S. Olmsted , J. L. Padgett , A. I. Yudin , K. J. Whaley , T. R. Moench , R. A. Cone , Biophys. J. 2001, 81, 1930. 11566767 10.1016/S0006-3495(01)75844-4 PMC1301668

[188] J. Reinholz , K. Landfester , V. Mailänder , Drug Delivery 2018, 25, 1694. 30394120 10.1080/10717544.2018.1501119 PMC6225504

[189] S. Li , H. Zhang , K. Chen , M. Jin , S. H. Vu , S. Jung , N. He , Z. Zheng , M.‐S. Lee , Drug Delivery 2022, 29, 1142. 35384787 10.1080/10717544.2022.2058646 PMC9004504

[190] L. Liu , W. Yao , Y. Rao , X. Lu , J. Gao , Drug Delivery 2017, 24, 569. 28195032 10.1080/10717544.2017.1279238 PMC8241197

[191] S. Lai , K. Hida , S. Man , C. Chen , C. Machamer , T. Schroer , J. Hanes , Biomaterials 2007, 28, 2876. 17363053 10.1016/j.biomaterials.2007.02.021

[192] J. A. Wang , T. F. Meyer , T. Rudel , Int. J. Med. Microbiol. 2008, 298, 209. 17683982 10.1016/j.ijmm.2007.05.004

[193] S. K. Lai , Y.‐Y. Wang , J. Hanes , Adv. Drug Delivery Rev. 2009, 61, 158. 10.1016/j.addr.2008.11.002 PMC2667119 19133304

[194] I. Pereira De Sousa , C. Steiner , M. Schmutzler , M. D. Wilcox , G. J. Veldhuis , J. P. Pearson , C. W. Huck , W. Salvenmoser , A. Bernkop‐Schnürch , Eur. J. Pharm. Biopharm. 2015, 97, 273. 25576256 10.1016/j.ejpb.2014.12.024

[195] F. Hintzen , G. Perera , S. Hauptstein , C. Müller , F. Laffleur , A. Bernkop‐Schnürch , Int. J. Pharm. 2014, 472, 20. 24879935 10.1016/j.ijpharm.2014.05.047

[196] S. Dünnhaupt , O. Kammona , C. Waldner , C. Kiparissides , A. Bernkop‐Schnürch , Eur. J. Pharm. Biopharm. 2015, 96, 447. 25712487 10.1016/j.ejpb.2015.01.022

[197] C. Müller , K. Leithner , S. Hauptstein , F. Hintzen , W. Salvenmoser , A. Bernkop-Schnürch , J. Nanopart. Res. 2013, 15, 1353.

[198] A. M. Bannunah , D. Vllasaliu , J. Lord , S. Stolnik , Mol. Pharm. 2014, 11, 4363. 25327847 10.1021/mp500439c

[199] B. He , P. Lin , Z. Jia , W. Du , W. Qu , L. Yuan , W. Dai , H. Zhang , X. Wang , J. Wang , X. Zhang , Q. Zhang , Biomaterials 2013, 34, 6082. 23694903 10.1016/j.biomaterials.2013.04.053

[200] M. S. Ehrenberg , A. E. Friedman , J. N. Finkelstein , G. Oberdörster , J. L. Mcgrath , Biomaterials 2009, 30, 603. 19012960 10.1016/j.biomaterials.2008.09.050

[201] D. Yang , X. Lü , Y. Hong , T. Xi , D. Zhang , Biomaterials 2013, 34, 5747. 23660250 10.1016/j.biomaterials.2013.04.028

[202] D. Dutta , S. K. Sundaram , J. G. Teeguarden , B. J. Riley , L. S. Fifield , J. M. Jacobs , S. R. Addleman , G. A. Kaysen , B. M. Moudgil , T. J. Weber , Toxicol. Sci. 2007, 100, 303. 17709331 10.1093/toxsci/kfm217

[203] G. Apodaca , Traffic 2001, 2, 149. 11260520 10.1034/j.1600-0854.2001.020301.x

[204] A. C. Anselmo , S. Mitragotri , Bioeng. Transl. Med. 2019, 4, e10143. 31572799 10.1002/btm2.10143 PMC6764803

[205] A. V. Singh , P. Bhardwaj , A. K. Upadhyay , A. Pagani , J. Upadhyay , J. Bhadra , V. Tisato , M. Thakur , D. Gemmati , R. Mishra , P. Zamboni , Explor. BioMat‐X 2024, 1, 124.

[206] A. M. Vargason , A. C. Anselmo , S. Mitragotri , Nat. Biomed. Eng. 2021, 5, 951. 33795852 10.1038/s41551-021-00698-w

[207] A. C. Eifler , C. S. Thaxton , Methods Mol. Biol. 2011, 726, 325. 21424459 10.1007/978-1-61779-052-2_21

[208] M. L. Etheridge , S. A. Campbell , A. G. Erdman , C. L. Haynes , S. M. Wolf , J. Mccullough , Nanomedicine 2013, 9, 1. 22684017 10.1016/j.nano.2012.05.013 PMC4467093

[209] G. Pillai , Nanomedicines for Cancer Therapy : An Update of FDA Approved and those Under Various Stages of Development, Symbiosis, Louisville, KY 2014.

[210] A. A. Halwani , Pharmaceutics 2022, 14, 106.35057002

[211] R. Korsmeyer , Regener. Biomater. 2016, 3, 143. 10.1093/rb/rbw011 PMC4817320 27047683

[212] Y. Barenholz , J. Controlled Release 2012, 160, 117. 10.1016/j.jconrel.2012.03.020 22484195

[213] DOXIL Approved by FDA , AIDS Patient Care 1995, 9, 306. 11361446

[214] FDA approves KS drug, Food and Drug Administration, Florida Department of Health and Rehabilitation Services , AIDS Alert 1996, 11, 11. 11363227

[215] U. Kanwal , N. Irfan Bukhari , M. Ovais , N. Abass , K. Hussain , A. Raza , J. Drug Targets 2018, 26, 296. 10.1080/1061186X.2017.1380655 28906159

[216] G. K. Rout , H.‐S. Shin , S. Gouda , S. Sahoo , G. Das , L. F. Fraceto , J. K. Patra , Artif. Cells Nanomed. Biotechnol. 2018, 46, 1053. 29879850 10.1080/21691401.2018.1478843

[217] S. Svenson , D. Tomalia , Adv. Drug Delivery Rev. 2005, 57, 2106. 10.1016/j.addr.2005.09.018 16305813

[218] E. Abbasi , S. F. Aval , A. Akbarzadeh , M. Milani , H. T. Nasrabadi , S. W. Joo , Y. Hanifehpour , K. Nejati‐Koshki , R. Pashaei‐Asl , Nanoscale Res. Lett. 2014, 9, 247. 24994950 10.1186/1556-276X-9-247 PMC4074873

[219] N. Jain , R. Jain , N. Thakur , B. Gupta , D. Jain , J. Banveer , S. Jain , Asian J. Pharm. Clin. Res. 2010, 3, 159.

[220] R. Singh , J. W. Lillard Jr. , Exp. Mol. Pathol. 2009, 86, 215. 19186176 10.1016/j.yexmp.2008.12.004 PMC3249419

[221] D. Reker , Y. Rybakova , A. R. Kirtane , R. Cao , J. W. Yang , N. Navamajiti , A. Gardner , R. M. Zhang , T. Esfandiary , J. L’Heureux , T. Von Erlach , E. M. Smekalova , D. Leboeuf , K. Hess , A. Lopes , J. Rogner , J. Collins , S. M. Tamang , K. Ishida , P. Chamberlain , D. Yun , A. Lytton‐Jean , C. K. Soule , J. H. Cheah , A. M. Hayward , R. Langer , G. Traverso , Nat. Nanotechnol. 2021, 16, 725. 33767382 10.1038/s41565-021-00870-y PMC8197729

[222] A. V. Singh , M. Varma , M. Rai , S. Pratap Singh , G. Bansod , P. Laux , A. Luch , Adv. Intell. Syst. 2024, 6, 2300366.

[223] A. V. Singh , A. Shelar , M. Rai , P. Laux , M. Thakur , I. Dosnkyi , G. Santomauro , A. K. Singh , A. Luch , R. Patil , J. Bill , J. Agric. Food Chem. 2024, 72, 2835. 38315814 10.1021/acs.jafc.3c06466

[224] R. Najjar , Diagnostics 2023, 13, 2760. 37685300 10.3390/diagnostics13172760 PMC10487271

[225] X. Tang , BJR Open 2020, 2, 20190031. 33178962 10.1259/bjro.20190031 PMC7594889

[226] P. Papadimitroulas , L. Brocki , N. Christopher Chung , W. Marchadour , F. Vermet , L. Gaubert , V. Eleftheriadis , D. Plachouris , D. Visvikis , G. C. Kagadis , M. Hatt , Phys. Med. 2021, 83, 108. 33765601 10.1016/j.ejmp.2021.03.009

[227] L. K. Vora , A. D. Gholap , K. Jetha , R. R. S. Thakur , H. K. Solanki , V. P. Chavda , Pharmaceutics 2023, 15, 1916. 37514102 10.3390/pharmaceutics15071916 PMC10385763

[228] D. Ho , P. Wang , T. Kee , Nanoscale Horiz. 2019, 4, 365. 32254089 10.1039/c8nh00233a

[229] R. Dhudum , A. Ganeshpurkar , A. Pawar , Drugs Drug Candidates 2024, 3, 148.

[230] Y. Sun , Y. Peng , Y. Chen , A. J. Shukla , Adv. Drug Delivery Rev. 2003, 55, 1201. 10.1016/s0169-409x(03)00119-4 12954199

[231] S. Chaudhary , P. Muthudoss , T. Madheswaran , A. Paudel , V. Gaikwad , in A Handbook of Artificial Intelligence in Drug Delivery (Ed: A. Philip , A. Shahiwala , M. Rashid , M. Faiyazuddin ), Academic Press, Cambridge, MA 2023, pp. 395–442.

[232] F. Behgounia , B. Zohuri , Res. J. Biol. Sci. 2020, 6, 1.

[233] K. P. Das , J. Chandra., Front. Med. Technol. 2023, 4, 1067144. 36688144 10.3389/fmedt.2022.1067144 PMC9853978

[234] T. M. Ludden , Clin. Pharmacokinetics 1991, 20, 429. 10.2165/00003088-199120060-00001 2044328

[235] J. Taskinen , J. Yliruusi , Adv. Drug Delivery Rev. 2003, 55, 1163. 10.1016/s0169-409x(03)00117-0 12954197

[236] I. F. Tsigelny , Briefings Bioinf. 2019, 20, 1434. 10.1093/bib/bby004 29438494

[237] P. Guo , Y. Wang , H. Cui , X. Yao , G. Guan , M. Han , Small Sci. 2024, 4, 2300227. 10.1002/smsc.202300227 PMC11935218 40212998

[238] L. Messager , J. Gaitzsch , L. Chierico , G. Battaglia , Curr. Opin. Pharmacol. 2014, 18, 104. 25306248 10.1016/j.coph.2014.09.017

[239] H. Sawczyc , S. Heit , A. Watts , Eur. Biophys. J. 2023, 52, 39. 36786921 10.1007/s00249-023-01632-5 PMC10039845

[240] E. Nance , S. H. Pun , R. Saigal , D. L. Sellers , Nat. Rev. Mater. 2022, 7, 314. 38464996 10.1038/s41578-021-00394-w PMC10923597

[241] A. Poustforoosh , M. H. Nematollahi , H. Hashemipour , A. Pardakhty , J. Controlled Release 2022, 343, 777. 10.1016/j.jconrel.2022.02.015 35183653

[242] E. Puris , G. Fricker , M. Gynther , Pharm. Res. 2022, 39, 1415. 35359241 10.1007/s11095-022-03241-x PMC9246765

[243] G. S. R. Raju , E. Pavitra , G. L. Varaprasad , S. S. Bandaru , G. P. Nagaraju , B. Farran , Y. S. Huh , Y.‐K. Han , J. Nanobiotechnol. 2022, 20, 274. 10.1186/s12951-022-01476-9 PMC9195263 35701781

[244] M. Xu , S. Li , Cancer Lett. 2023, 574, 216397. 37730105 10.1016/j.canlet.2023.216397

[245] C. Belli , D. Trapani , G. Viale , P. D'amico , B. A. Duso , P. Della Vigna , F. Orsi , G. Curigliano , Cancer Treat. Rev. 2018, 65, 22. 29502037 10.1016/j.ctrv.2018.02.004

[246] E. Henke , R. Nandigama , S. Ergün , Front. Mol. Biosci. 2020, 6, 160. 32118030 10.3389/fmolb.2019.00160 PMC7025524

[247] J. Y. Yhee , S. Jeon , H. Y. Yoon , M. K. Shim , H. Ko , J. Min , J. H. Na , H. Chang , H. Han , J.‐H. Kim , M. Suh , H. Lee , J. H. Park , K. Kim , I. C. Kwon , J. Controlled Release 2017, 267, 223. 10.1016/j.jconrel.2017.09.015 28917532

[248] Y.‐C. Chen , Y.‐C. Chu , C.‐Y. Huang , Y.‐T. Lee , W.‐Y. Lee , C.‐Y. Hsu , A. C. Yang , W.‐H. Liao , Y.‐F. Cheng , eClinicalMedicine 2022, 51, 101543. 35856040 10.1016/j.eclinm.2022.101543 PMC9287624

[249] J. Zhou , M. Du , S. Chang , Z. Chen , Cardiovasc. Ultrasound 2021, 19, 29. 34416899 10.1186/s12947-021-00261-2 PMC8379752

[250] C. Caudai , A. Galizia , F. Geraci , L. Le Pera , V. Morea , E. Salerno , A. Via , T. Colombo , Comput. Struct. Biotechnol. J. 2021, 19, 5762. 34765093 10.1016/j.csbj.2021.10.009 PMC8566780

[251] Q. Lin , P. K.‐H. Tam , C. S.‐M. Tang , Front. Pediatr. 2023, 11, 1203289. 37593442 10.3389/fped.2023.1203289 PMC10429173

[252] R. H. J. A. Slart , M. C. Williams , L. E. Juarez‐Orozco , C. Rischpler , M. R. Dweck , A. W. J. M. Glaudemans , A. Gimelli , P. Georgoulias , O. Gheysens , O. Gaemperli , G. Habib , R. Hustinx , B. Cosyns , H. J. Verberne , F. Hyafil , P. A. Erba , M. Lubberink , P. Slomka , I. Išgum , D. Visvikis , M. Kolossváry , A. Saraste , Eur. J. Nucl. Med. Mol. Imaging 2021, 48, 1399. 33864509 10.1007/s00259-021-05341-z PMC8113178

[253] S. Nazir , D. M. Dickson , M. U. Akram , Comput. Biol. Med. 2023, 156, 106668. 36863192 10.1016/j.compbiomed.2023.106668

[254] E. Badidi , Future Internet 2023, 15, 370.

[255] A. N. Belkacem , S. Ouhbi , A. Lakas , E. Benkhelifa , C. Chen , Front. Med. 2021, 8, 585578. 10.3389/fmed.2021.585578 PMC8044874 33869239

[256] C. Carini , A. A. Seyhan , J. Transl. Med. 2024, 22, 411. 38702711 10.1186/s12967-024-05067-0 PMC11069149

[257] V. B. Patravale , S. D. Mandawgade , Int. J. Cosmet. Sci. 2008, 30, 19. 18377627 10.1111/j.1468-2494.2008.00416.x

[258] A. Rehman , Q. Tong , S. M. Jafari , E. Assadpour , Q. Shehzad , R. M. Aadil , M. W. Iqbal , M. M. A. Rashed , B. S. Mushtaq , W. Ashraf , Adv. Colloid Interface Sci. 2020, 275, 102048. 31757387 10.1016/j.cis.2019.102048

[259] C. M. Paleos , D. Tsiourvas , Z. Sideratou , A. Pantos , J. Controlled Release 2013, 170, 141. 10.1016/j.jconrel.2013.05.011 23707326

[260] S. Hua , Front. Pharmacol. 2015, 6, 219. 26483690 10.3389/fphar.2015.00219 PMC4588690

[261] W. Yang , B. Wang , G. Lei , G. Chen , D. Liu , Front. Bioeng. Biotechnol. 2022, 10, 974646. 36051593 10.3389/fbioe.2022.974646 PMC9424858

[262] H. Zhang , J. Bussmann , F. H. Huhnke , J. Devoldere , A. Minnaert , W. Jiskoot , F. Serwane , J. Spatz , M. Röding , S. C. De Smedt , K. Braeckmans , K. Remaut , Adv. Sci. 2022, 9, 2102072. 10.1002/advs.202102072 PMC8811815 34913603

[263] W. Baschong , C. Artmann , D. Hueglin , J. Roeding , J. Cosmet. Sci. 2001, 52, 155. 11413495

[264] A. Pandita , P. Sharma , ISRN Pharm. 2013, 2013, 348186. 24106615 10.1155/2013/348186 PMC3782844

[265] C. Biolabs , Photosomes‐Based Drug Delivery Service, https://www.creative‐biolabs.com/lipid‐based‐delivery/photosomes‐based‐drug‐delivery‐service.htm (accessed: 2023).

[266] A. Zou , Y. Li , Y. Chen , A. Angelova , V. M. Garamus , N. Li , M. Drechsler , B. Angelov , Y. Gong , Colloids Surf., B 2017, 153, 310. 10.1016/j.colsurfb.2017.02.031 28285062

[267] C. Biolabs , Ultrasomes‐Based Drug Delivery Service, https://www.creative‐biolabs.com/lipid‐based‐delivery/ultrasomes‐based‐drug‐delivery‐service.htm?gclid=Cj0KCQiA35urBhDCARIsAOU7QwmikzqMHOVmaEqGYF2tIUEeaCqiAQzDkfHm9DL13mEyvEbmbSV3uFgaAt24EALw_wcB (accessed: 2023).

[268] K. Asadi , A. Gholami , Int. J. Biol. Macromol. 2021, 182, 648. 33862071 10.1016/j.ijbiomac.2021.04.005 PMC8049750

[269] L. Ordóñez‐Gutiérrez , F. Re , E. Bereczki , E. Ioja , M. Gregori , A. J. Andersen , M. Antón , S. M. Moghimi , J.‐J. Pei , M. Masserini , F. Wandosell , Nanomedicine 2015, 11, 421. 25461285 10.1016/j.nano.2014.09.015

[270] M. Taylor , S. Moore , S. Mourtas , A. Niarakis , F. Re , C. Zona , B. L. Ferla , F. Nicotra , M. Masserini , S. G. Antimisiaris , M. Gregori , D. Allsop , Nanomedicine 2011, 7, 541. 21722618 10.1016/j.nano.2011.06.015

[271] M. Gobbi , F. Re , M. Canovi , M. Beeg , M. Gregori , S. Sesana , S. Sonnino , D. Brogioli , C. Musicanti , P. Gasco , M. Salmona , M. E. Masserini , Biomaterials 2010, 31, 6519. 20553982 10.1016/j.biomaterials.2010.04.044

[272] P. Wang , H. Kouyoumdjian , D. C. Zhu , X. Huang , Carbohydr. Res. 2015, 405, 110. 25498198 10.1016/j.carres.2014.07.020 PMC4314519

[273] M. Lindgren , M. Hällbrink , A. Prochiantz , Ü. Langel , Trends Pharmacol. Sci. 2000, 21, 99. 10689363 10.1016/s0165-6147(00)01447-4

[274] G. Drin , C. Rousselle , J.‐M. Scherrmann , A. R. Rees , J. Temsamani , AAPS PharmSci 2015, 4, 26. 10.1208/ps040426 PMC2751315 12645998

[275] K. Zeller , S. Rahner‐Welsch , W. Kuschinsky , J. Cereb. Blood Flow Metab. 1997, 17, 204. 9040500 10.1097/00004647-199702000-00010

[276] T. Nishioka , Y. Oda , Y. Seino , T. Yamamoto , N. Inagaki , H. Yano , H. Imura , R. Shigemoto , H. Kikuchi , Cancer Res. 1992, 52, 3972. 1617673

[277] A. Béduneau , P. Saulnier , J.‐P. Benoit , Biomaterials 2007, 28, 4947. 17716726 10.1016/j.biomaterials.2007.06.011

[278] D. D. Allen , W. J. Geldenhuys , Life Sci. 2006, 78, 1029. 16126231 10.1016/j.lfs.2005.06.004

[279] H. Xia , X. Gao , G. Gu , Z. Liu , Q. Hu , Y. Tu , Q. Song , L. Yao , Z. Pang , X. Jiang , J. Chen , H. Chen , Int. J. Pharm. 2012, 436, 840. 22841849 10.1016/j.ijpharm.2012.07.029

[280] T. Kanazawa , K. Morisaki , S. Suzuki , Y. Takashima , Mol. Pharm. 2014, 11, 1471. 24708261 10.1021/mp400644e

[281] S. Console , C. Marty , C. García‐Echeverría , R. Schwendener , K. Ballmer‐Hofer , J. Biol. Chem. 2003, 278, 35109. 12837762 10.1074/jbc.M301726200

[282] M. K. Alam , Adv. NanoBiomed Res. 2023, 3, 2300041.

